# Circadian system and aging: where both times interact

**DOI:** 10.3389/fragi.2025.1646794

**Published:** 2025-10-17

**Authors:** Claudia García Cobarro, Lara Ignez Soares, Yevheniy Kutsenko, Antonia Tomas-Loba

**Affiliations:** Department of Physiology, Circadian Rhythm and Cancer Laboratory, Biomedical Research Institute of Murcia Pascual Parrilla–IMIB, Murcia University, Murcia, Spain

**Keywords:** circadian system, circadian aging, chrono-exposome, chronodisruption, long-lived species, chronotherapy, circadian plasticity, geroscience

## Abstract

Time shapes life both through its steady progression, as seen in aging, and through its eternal return, reflected in biological rhythms. These two temporal forces have sculpted organisms from their evolutionary beginnings, intertwining the processes of circadian regulation and senescence into the emerging concept of circadian aging. From the earliest prokaryotic lifeforms, the ability to sense and anticipate environmental cycles conferred evolutionary advantages, leading to the emergence of endogenous circadian clocks that regulate nearly every aspect of physiology. The mammalian circadian system is far more complex than a single master clock, comprising multiple tissue-specific oscillators entrained by diverse zeitgebers such as light, food, and activity. Importantly, circadian function deteriorates with age, contributing to hallmarks of aging including metabolic dysfunction, cognitive decline, immunosenescence, and disrupted sleep. Yet species with negligible senescence, such as naked mole-rats, tend to retain robust circadian rhythms throughout life, suggesting that temporal homeostasis may serve as both a marker and a modulator of healthy aging. This review explores the dynamic interplay between circadian time and chronological time, highlighting their shared regulatory pathways. We examine how circadian rhythms change naturally with age and in pathological conditions, the molecular crosstalk between clock genes and aging-related pathways and emerging evidence that circadian interventions can restore rhythmicity and promote healthspan. By unraveling the mechanisms of *circadian aging*, we aim to illuminate novel chrono-geroprotective strategies to enhance resilience and improve quality of life across the lifespan.

## 1 Introduction

Time is a fundamental variable in life. Everything unfolds along a timeline, making biological processes either linear and irreversible, as in the case of aging, or repetitive and cyclical, as seen in virtually all biological functions regulated by the circadian system. Since the earliest stages of life on Earth, these two dimensions of time have coexisted in a finely tuned homeostasis, giving rise to what we now recognize as circadian aging.

Since the earliest prokaryotic life, the ability to sense external time has provided a biological advantage. Anticipating the day/night cycle by activating appropriate molecular pathways or behaviors improved adaptation and protection against the exposome, the full range of environmental factors that affect human health. As a result, an inner mechanism has appeared that regulates nearly all aspects of our biology, including behavior (sleepiness, hunger, and other physiological perceptions), hormone secretion, gene expression, molecular localization, metabolism, epigenetic marks, the immune system, cell proliferation, or, even more, the efficacy of therapy administration, according to time. This is the circadian system: a complex network that orchestrates that everything occurs cyclically, rhythmically, at the proper time to preserve our homeostasis.

**FIGURE 1:**
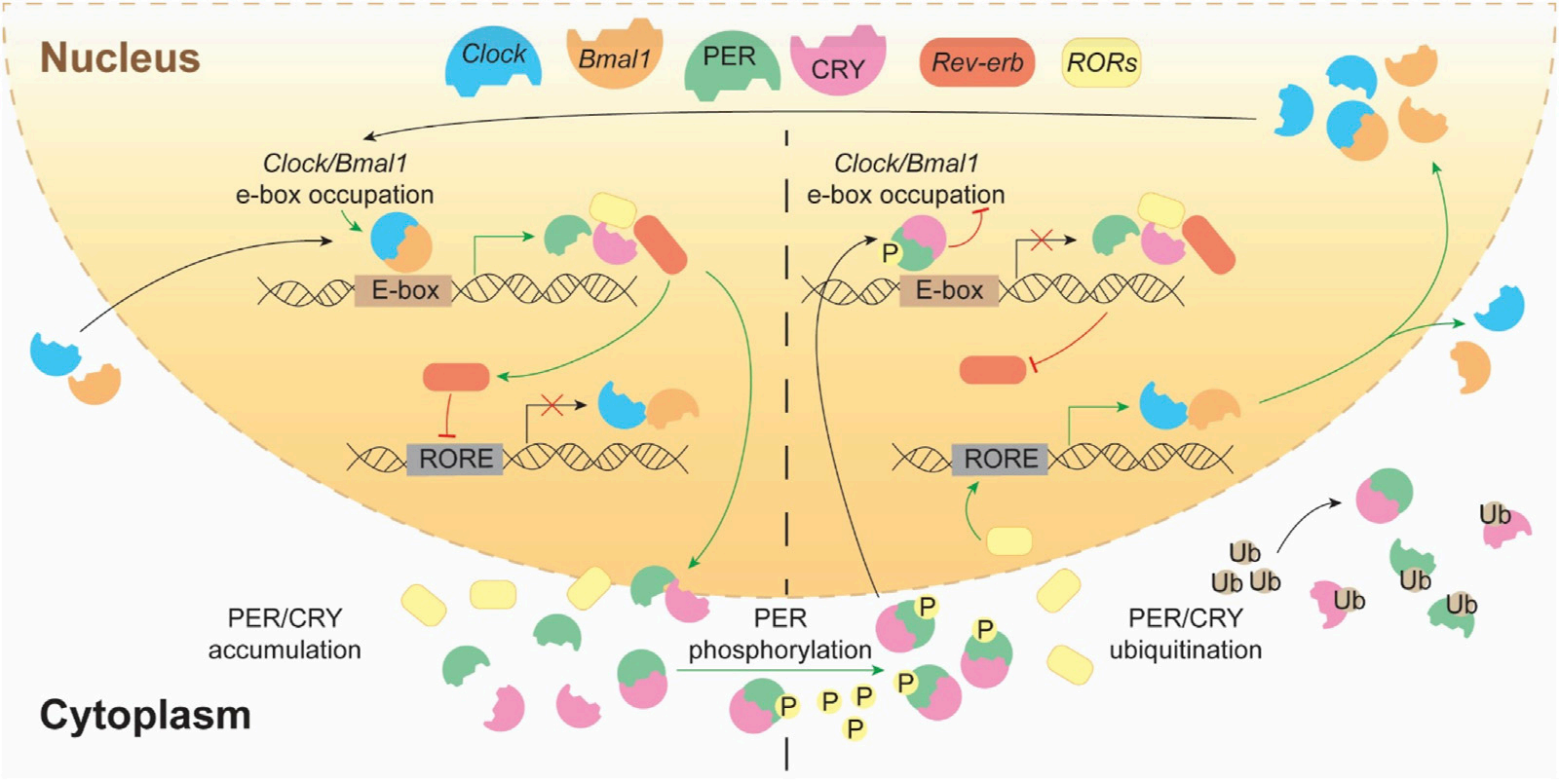
The transcriptional–translational feedback loops of the mammalian circadian clock. This schematic represents the core molecular architecture of the circadian clock, highlighting the dynamic interplay between transcriptional activation and repression across the 24-hour cycle. The heterodimeric complex CLOCK:BMAL1 binds to E-box elements in the promoters of clock-controlled genes, promoting the transcription of Period (*Per*) and Cryptochrome (*Cry*) genes, as well as *Rev-erbα/β* and *Rorα/β/γ*. The translated PER and CRY proteins accumulate in the cytoplasm and form complexes that eventually translocate into the nucleus. As its protein concentration progresses (right side), PER/CRY complexes translocate into the nucleus, where they inhibit CLOCK:BMAL1-mediated transcription, closing the negative feedback loop. PER proteins are phosphorylated by kinases (e.g., CK1δ/ε), which targets them for ubiquitination and proteasomal degradation, allowing the cycle to restart. Meanwhile, REV-ERBs and RORs form an auxiliary loop by rhythmically repressing or activating *Bmal1* transcription through binding to RORE elements, thereby reinforcing the oscillatory robustness of the system.

At the molecular level, in mammals, circadian rhythms are regulated by transcriptional, post-translational and methylation feedback loops generated by a set of interplaying clock proteins (Orozco-Solis and Aguilar-Arnal, 2020). At the core of the mammalian molecular circadian clock, the transcription factors CLOCK and BMAL1/NPAS form a heterodimer that activates the expression of clock-controlled genes by binding to E-box elements, initiating the cycle. Among these target genes are *Per* and *Cry* families. Period proteins PER1–3 and Cryptochrome ones CRY1–2 form complexes, and translocate to the nucleus to inhibit CLOCK-BMAL1 activity, thus closing the negative feedback loop. To start a new cycle, PER and CRY proteins must be degraded via proteasomal degradation (through phosphorylation by CK1δ/ε, AMPK, and other kinases) relieves their inhibition of CLOCK-BMAL1 activity to re-start again the cycle (Eide et al., 2005; Lamia et al., 2009; Yoo et al., 2013; Masuda et al., 2020). Moreover, BMAL1–CLOCK heterodimer also drives the expression of *NR1D1-2* genes, that encode REV-ERBα-β proteins respectively, and DBP. DBP binds D-box motifs to drive expression of genes encoding the transcriptional activators *RORα* and *RORβ* which compete with REV-ERBα and REV-ERBβ for binding to RORE elements as those located in *Bmal1*. These regulatory loops not only induce the expression of their core components but also regulate many other genes involved in key homeostatic processes, including metabolism or DNA replication (Cox and Takahashi, 2019; Mortimer et al., 2024). They also coordinate with epigenetic regulators and tissue-specific transcription factors to drive rhythmic gene expression (Mortimer et al., 2025) (Figure 1).

**FIGURE 2:**
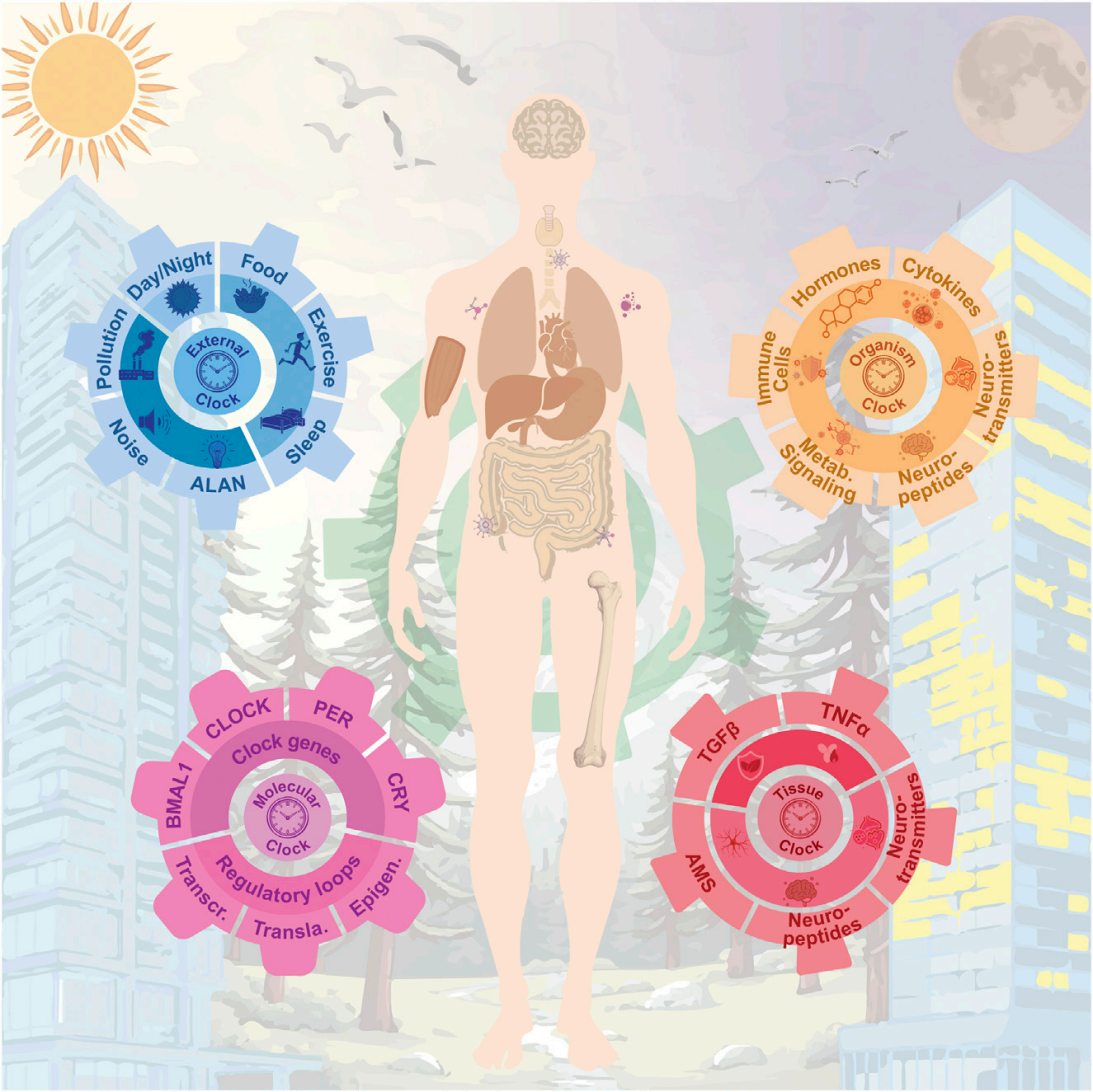
Multilayered architecture of the circadian system and its interplay with environmental, physiological, and molecular rhythms across the human body. At the core (purple gear), the molecular circadian clock. These intrinsic oscillations are modulated by external cues (blue gear), including artificial light at night, all of which contribute to entrainment and synchronization. The intermediate layer (orange gear) consists of organismal signals such as hormones, cytokines, and neuropeptides, which coordinate systemic outputs. Finally, tissue-specific and cellular-level clocks (red gear) govern local physiology and niche-specific functions, including stem cell behavior, neuropeptide secretion, and intercellular signaling.

Beyond the molecular perspective, the circadian system encompasses additional layers of regulation (Figure 2). At the tissue level, paracrine signals are required to synchronize cell populations. In this regard, factors such as TGFβ, TNFα, and neurotransmitters including GABA, VIP, and AVP play crucial roles in coupling cell-autonomous circadian oscillators (Yoshida et al., 2018; Finger et al., 2021; Ono et al., 2021). At a higher level, organisms must also coordinate their rhythms to align physiology in a time-dependent manner. In this context, hormones such as melatonin and cortisol, as well as circulating elements in blood or lymphatic fluid, including immune cells, neuropeptides, and neurotransmitters like dopamine, noradrenaline, and serotonin, are key components of the circadian system (Linsell et al., 1985; Ciarleglio et al., 2011; Freyberg and McCarthy, 2017; Bonmati-Carrion and Tomas-Loba, 2021). Given the complexity of blood composition, the existence of yet undiscovered systemic circadian regulators cannot be ruled out. While both the organismal and tissue clocks act as endogenous entrainers, they operate at different scales and through distinct regulatory mechanisms: organismal clocks integrate and distribute systemic time cues, whereas tissue clocks require local synchronization, often via paracrine signaling, to ensure precise timing of specialized functions. Finally, ecosystem temporal cues, such as natural or artificial light and temperature, as well as behavioral inputs, including the social agenda and timing of nutrition, are also critical components of this complex system.

Time also occurs in a linear setting, very well represented by the irreversible process of aging. All living beings age, with the exception of a few organisms, including hydras (*Hydra vulgaris*) (Suknovic et al., 2021), jellyfishes (*Turritopsis dohrnii)* (Pascual-Torner et al., 2022) that escape this fate. Additionally, there are others in which aging progresses very slowly, like American lobsters (*Homarus americanus*) (Polinski et al., 2021), long-lived turtles (*Testudines*), cavefishes (including *Phreatobius sanguijuela* and *Prietella phreatophila*), and naked mole-rats (*Heterocephalus glaber*) (Montazid et al., 2023). Interestingly, species that exhibit delayed aging also tend to preserve solid circadian rhythms across their lifespan, suggesting that the maintenance of temporal homeostasis may be a hallmark of healthy aging (López-Otín and Kroemer, 2021). Notably, several aging-related signaling pathways are interconnected with the molecular clockwork. Among these, SIRT1, mTOR, AMPK, and insulin signaling present the strongest experimental support, with mechanistic studies across multiple species, including mammals. Others, such as FOXO and NRF2, are increasingly supported but still require deeper mechanistic resolution (Ramanathan et al., 2018; Acosta-Rodríguez et al., 2022; Das et al., 2023; Chhunchha et al., 2020).

In aging mammals, these rhythms tend to adapt to the age stage by modifying its period, phase, and amplitude until later life, when these rhythms fragment or dampen, contributing to metabolic dysfunction, cognitive decline, immunosenescence, and sleep disruption (Liu et al., 2024). However, long-lived span organisms maintain stable internal rhythms over decades, highlighting the possibility that resilience of circadian oscillations, at molecular, cellular, and systemic levels, might underlie their sustained homeostasis. Indeed, recent studies show that age-related circadian decline may not be inevitable, but modifiable by enhancing circadian amplitude via light, feeding schedules, or genetic interventions. These interventions improve metabolic and cognitive function in aged mice, reinforcing the idea that the preservation of biological timing could be as critical to longevity and suggesting that negligible aging may, in part, reflect the capacity to maintain circadian synchrony in the face of time (Welz and Benitah, 2020; Belancio et al., 2015; Acosta-Rodríguez et al., 2022; Altamirano et al., 2024; Whittaker et al., 2023; Acosta-Rodríguez et al., 2021).

Circadian aging describes the convergence of linear time, associated with aging, and cyclical time, governed by circadian rhythms. At this intersection, circadian robustness declines, and aging phenotypes emerge in a mutually reinforcing process. Evidence indicates that restoring circadian function can improve health span, offering opportunities for chrono-geroprotective strategies. This review explores how circadian and aging processes interact in mice, humans and long-lived species, aiming to uncover mechanisms of chrono-aging and their implications for healthy longevity.

## 2 When the circadian system meets aging in the natural timeline

**FIGURE 3:**
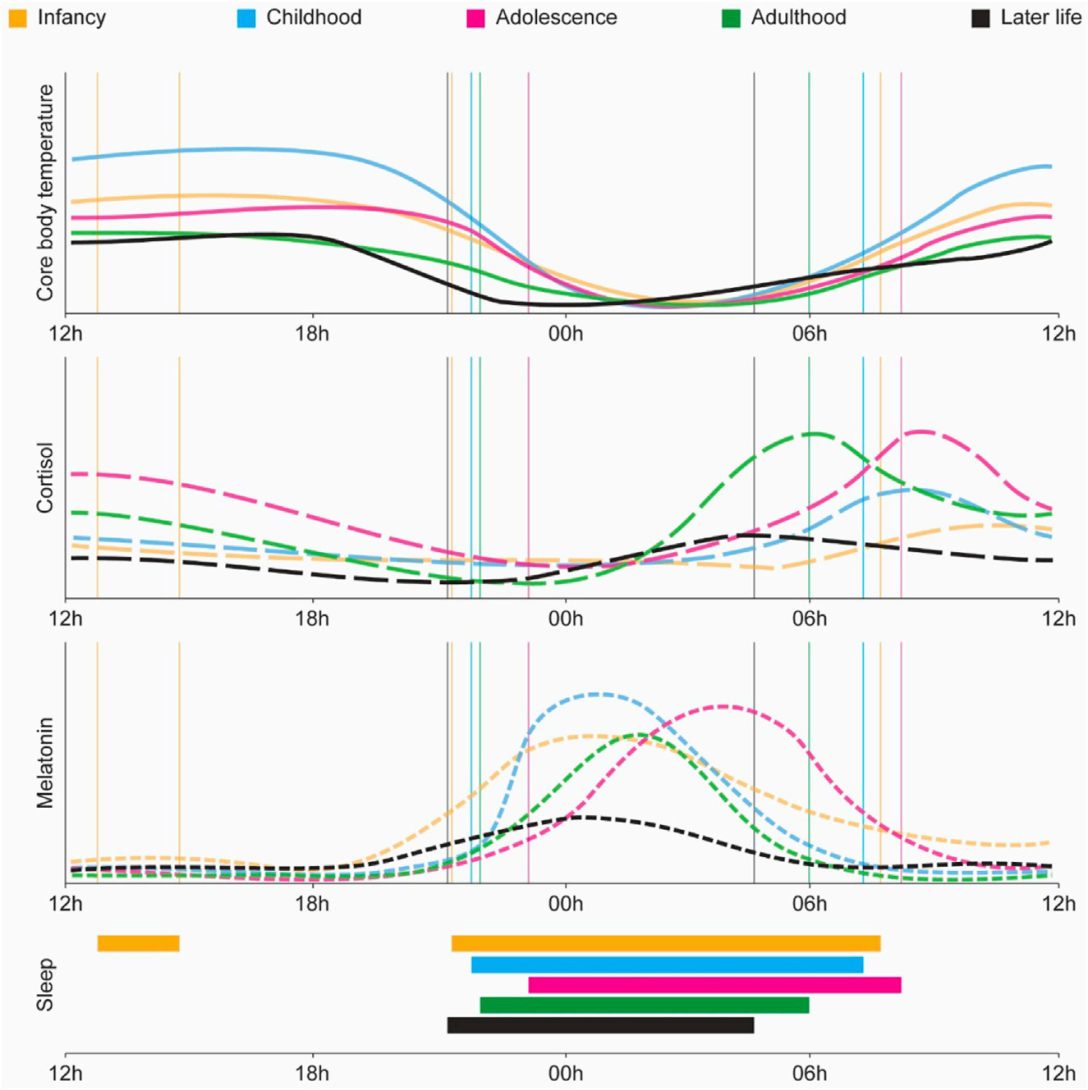
Age-related changes in physiological circadian variables across the lifespan. Circadian rhythms in core physiological variables evolve dynamically from infancy through later life. From top to bottom: core body temperature, cortisol, melatonin, and sleep timing across five life stages: infancy (orange), childhood (blue), adolescence (magenta), adulthood (green), and later life (black). Core body temperature exhibits a progressive shift in phase and amplitude, with adolescence marked by a delayed nadir and later life showing a dampened rhythm. Cortisol peaks in the early morning across all stages, but its amplitude is highest during adolescence and adulthood, declining significantly in older age. Melatonin secretion also follows a robust circadian pattern that matures in childhood, peaks in adolescence with a delayed phase, and gradually loses amplitude and rhythmic precision in aging. Sleep timing (bottom panel) transitions from polyphasic and fragmented sleep in infancy to consolidated nocturnal sleep in adulthood, with delayed sleep onset in adolescence and a tendency for phase advance and fragmentation in later life (Logan and McClung, 2019).

Most physiological and molecular processes in the organism are governed by the circadian system, which generates rhythmic oscillations throughout the lifespan. These rhythms adapt across different stages of life, fulfilling age-specific physiological requirements. At birth, neonates lack a fully matured central circadian clock, resulting in fragmented sleep-wake cycles. As the suprachiasmatic nuclei, in the brain, progressively synchronize with environmental cues, key circadian outputs, such as core body temperature, cortisol, and melatonin secretion, begin to exhibit defined periods, phases, and amplitudes. However, in advanced age, these rhythms often become dampened or desynchronized, leading to reduced circadian robustness and greater physiological variability (Figure 3).

Among the circadian changes across the lifespan described in Figure 3, there are several critical windows in which circadian control shifts and aging-associated phenotypes become more pronounced. Between the ages of 45–64, defined as middle age, disruptions in sleep architecture and circadian regulation begin to undergo significant transformations, with a remarkable change-point at the age of 60, that signals the early decline of the circadian system (Landolt et al., 1996; Carrier et al., 1997; Carrier et al., 2001; Carrier et al., 2005; Dijk and Duffy, 1999). Studies show that by their 40s and 50s, individuals already experience more fragmented sleep and reduced capacity to recover after extended wakefulness that drives a diminished ability to cope with stressors affecting sleep (Gaudreau et al., 2001). Comparative analyses reveal that middle-aged adults display a phase advance in both sleep timing and core body temperature rhythms, waking and sleeping earlier than younger adults, consistent with a reorientation of the circadian phase, even if the overall rhythmic amplitude remains stable (Carrier et al., 2002; Duffy et al., 2015). Moreover, melatonin rhythms also show early signs of decline. Starting around age 40, melatonin amplitude diminishes, down to about 60% of that seen in younger individuals, accompanied by lower daytime levels and prolonged nighttime peaks (Zhou et al., 2003). Notably, these changes occur independently of shifts in light exposure, indicating that the aging of the circadian system itself may drive this early erosion in temporal organization (Kawinska et al., 2005).

Studies in middle-aged female rats have shown that there are significant differences in the pattern of glucose utilization in the suprachiasmatic nucleus compared to young rats. The authors suggest that alterations in the synchronization and amplitude of luteinizing hormone peaks induced by estradiol during the transition to infertility in middle age could be triggering these changes (Wise et al., 1988). Menopause debut with a decline in estrogen levels, which has been linked to increased oxidative stress, an aging-driving agent (Rangel-Zuñiga et al., 2017). Moreover, postmenopausal women show reduced circadian robustness compared to premenopausal women, with lower amplitude in wrist temperature rhythms, lower average core body temperature during the sleep midopoint, earlier chronotype with a phase advance of approximately 1 hour, and blunted cortisol fluctuations (Gómez-Santos et al., 2016). In addition, they experienced greater sleep fragmentation and a higher frequency of sleep-related breathing abnormalities, such as apnea (Gómez-Santos et al., 2016). The loss of estrogen disrupts circadian rhythms, altering *Per2* and *Per3* gene expression in visceral and subcutaneous adipose tissue, respectively (Hernandez-Morante et al., 2012), which may contribute to fat redistribution and a higher risk of metabolic syndrome (Hernandez-Morante et al., 2012; Gómez-Santos et al., 2016; Verde et al., 2022), an aging-like phenotype. In fact, granulosa cells in women over 40 show a significant decrease in the expression of molecular clock genes, which negatively correlates with age (Jiang Z. et al., 2021). Additionally, apart from the *Rev-erbα* gene, all clock genes show also low expression levels in serum, which positively correlate with anti-Müllerian hormone levels (Jiang Z. et al., 2021). Overall, disrupted circadian rhythms in menopausal women are linked to increased multimorbidity and premature mortality (Ren et al., 2025) and coincide with other aging-like phenotypes that emerge profoundly, including bone demineralization, sarcopenia, skin and connective tissue decay or inflammaging.

At the peripheral level, in middle-aged individuals, and given the importance of circadian rhythms in lipid regulation and in their changing profile associated with metabolic problems (Dallmann et al., 2012; Gooley and Chua, 2014; Gooley, 2016; Rahman et al., 2023), it has been shown that the prevalence of endogenous circadian rhythms in the human plasma lipidome is maintained with healthy aging in middle age. Specifically, studies confirm that both young individuals and middle-aged individuals exhibit robust circadian regulation of the lipidome. However, in middle age, there is a reduction in the amplitude of lipid rhythmicity, a greater impact of factors such as sleep deprivation, a phase advance in the acrophase, and an alteration in the synchronization between central and lipid rhythms (Rahman et al., 2023).

Another step of time-fragility comes around age 70, when features such as sarcopenia rise steeply, causing accelerated loss of muscle mass and functionality, that are associated with an increased risk of falls, frailty, and mortality (Cruz-Jentoft and Sayer, 2019; Fernández-Martínez et al., 2023). Aging is one of the primary risk factors for the development of sarcopenia (López-Otín et al., 2023a), and age-related chronodisruption may initiate these pathways in skeletal muscle, preceding its onset (Fernández-Martínez et al., 2023). Notably, the core component of the circadian clock, *Bmal1*, regulates muscle homeostasis by controlling reactive oxygen species levels (ROS), so its decline with age promotes a pro-inflammatory environment (Kondratov et al., 2009). Over time, this situation leads to a chronic inflammatory state known as *inflammaging* (Franceschi and Campisi, 2014) characterized by the activation of the NF-κB pathway and an increase in the production of pro-inflammatory cytokines such as IL-6 and TNF-α (Fernández-Martínez et al., 2023). Sustained inflammation and the loss of circadian regulation interfere with muscle protein degradation and synthesis, also producing mitochondrial damage and thereby compromising energy production in muscle cells. These circadian metabolic changes contribute to the development and progression of sarcopenia. Indeed, *Bmal1* deficiency in preclinical models impairs circadian behavior and accelerates aging, leading to muscle atrophy, reduced strength, disrupted sarcomere organization, and decreased mitochondrial content, all key features of sarcopenia (Kondratov et al., 2006; Christian and Benian, 2020; Gao et al., 2020). Consistent with these observations, Yang et al. (2016) reported that the absence of BMAL1 in mice not only disrupts circadian rhythms but also increases oxidative stress, impairs mitochondrial function, and perturbs metabolic pathways, pointing to a clock-independent role for BMAL1 in maintaining redox balance, proteostasis, and tissue integrity. Strikingly, brain-specific restoration of *Bmal1* failed to rescue normal lifespan, underscoring the essential contribution of peripheral *Bmal1* to longevity (Yang et al., 2016). While this finding was discussed in the context of peripheral clock function, we speculate that it may also suggest that the central pacemaker’s role is less dominant than traditionally assumed, and that peripheral clocks can, under certain conditions, exert substantial autonomous control over specific physiological functions or even over organismal homeostasis.

This pronounced vulnerability around the seventh decade of life highlights the transition into a phase of systemic time-fragility, where the decline in circadian robustness intersects with the acceleration of aging phenotypes. As the circadian system becomes increasingly desynchronized, both centrally and peripherally, the organism’s capacity to adapt to environmental and physiological stressors is diminished. This vulnerability is not merely the result of internal degeneration, but is also shaped by lifelong interactions with the external environment. Indeed, the aging circadian system becomes more susceptible to exogenous influences, suggesting that exposures accumulated across a lifetime may converge with intrinsic molecular changes to further destabilize temporal homeostasis. This interplay sets the stage for understanding aging not only as a biological process but also as an environmentally modulated trajectory, one that unfolds under the constant influence of time-bound cues and stressors. Within this framework, we propose a new concept called the *chrono-exposome*, in which external cues play a particularly relevant role in the biology of time.

## 3 Chrono-Exposome and aging

The concept of the chrono-exposome encompasses the cumulative impact of environmental stressors on homeostatic processes throughout an individual’s life, particularly through their effects on the circadian system and, consequently, on the physiological functions it regulates. Across the entire lifespan, global exposures such as seasonal photoperiod changes, urbanicity, noise, social jetlag, sedentary indoor lifestyles, erratic feeding schedules, poor sleep routines, artificial light, psychosocial stress, and endocrine-disrupting chemicals pose chronic threats to circadian stability, potentially accelerating aging and disease Nahmod et al., 2019; Huang et al., 2024) (Table 1). It has been observed that the exposure to circadian entrainers, such as light, food, stress and exercise at the inappropriate time, i.e., during the rest phase, can shape the circadian system at the molecular level (Wolff and Esser, 2012; Bolsius et al., 2021), altering molecular clock expression in the suprachiasmatic nucleus and peripheral organs, and affecting metabolic processes. Among the different effects, it can increase oxidative stress and contribute to tissue damage (Li et al., 2023; Makris et al., 2023; Ruan et al., 2021). Through this interaction, circadian entrainers, although essential for life, have the potential to shape different signatures of aging depending on their timing and the life stage.

**TABLE 1:** Chrono-exposome factors affecting the circadian system and associated with age-related outcomes.

Life stage	Chrono-exposome factors	Circadian impact	Aging-like related outcome	References
Perinatal and Early Childhood	Maternal circadian disruption	Alters fetal clock development Impaired circadian gene expression rhythms	Reduced adult body mass Social avoidance Impaired metabolic gene expression rhythms	Smarr et al. (2017) Varcoe et al. (2013)
	Artificial lighting in neonatal units	Disruption of light-dark entrainment	Abnormal circadian behavior Irregular sleep-wake patterns Reduced weight gain Neurologic complications	Kok et al. (2024) Miller et al. (1995) Van Gilst et al. (2023)
	Feeding schedule misalignment	Impairs metabolic clock entrainment	Increase in body mass index and adiposity	Cheng et al. (2016), Van Gilst et al. (2023)
	Erratic sleep routines, screen exposure	Weakens circadian consolidation		Staples et al. (2021)
	Inadequate temperature regulation	Disrupts core body temperature rhythm entrainment	Poor sleep quality Less memory consolidation	Berger et al. (2023)
	Noise exposition	Circadian rhythm disruption Poor sleep quality	Cognitive impairment	Li et al. (2023)
Childhood & Adolescence	Early school start times	Induces chronic sleep deprivation and misalignment	Higher risk of risk of obesity and type 2 diabetes Elevated blood pressure and weakened immune function	Minges and Redeker (2016)
	Evening screen exposure	Delays melatonin secretion Inconsistent sleep patterns	Brain development, potentially influencing cognitive aging later in life	Telzer et al. (2015)
	Social jetlag	Leads to circadian misalignment	Mood and sleep disorders Increased body mass index Insulin resistance Elevated triglyceride levels	Magnusdottir et al. (2024) Pompeia et al. (2023)
	Indoor lifestyle	Reduces exposure to synchronizing morning light (reduce melatonin)		Westwood et al. (2023)
	Irregular dietary patterns	Disrupts peripheral clocks	Metabolic imbalances DNA methylation of metabolic circadian genes Insulin resistance	Asher and Sassone-Corsi (2015) Jansen et al. (2021) Garaulet et al. (2022)
	Physical inactivity	Weakens activity-related circadian cues Impaired muscle-Brain Crosstalk via Irisin and BDNF	Metabolic and hormonal imbalance	Zsuga et al. (2018)
Adulthood	Night/rotating shift work Jet lag (frequent travel)	Major disruption of central and peripheral clocks Attenuated Amplitude of Rhythms Melatonin suppression	Type 2 diabetes/metabolic syndrome Cardiovascular Dysfunction Disrupted DNA repair Desynchronized cell-cycle clock genes Disrupted NAD^+^ biosynthesis Cognitive decline Immune dysregulation Cancer risk Premature cellular and organismal aging (+1.3 years biological age acceleration) Sleep disturbances Reproductive aging	Shen et al. (2024) Caravia et al. (2022) Allada and Bass (2021) Yang et al. (2024) Ashimori et al. (2021)
	Chronic social jetlag	Recurrent misalignment of biological and social time	Type 2 diabetes/Obesity	Roenneberg et al. (2012) Roenneberg (2023)
	Artificial light at night	Suppresses melatonin and blunts circadian amplitude	Cancer risk (hormone-dependent)	Palomar-Cros et al. (2024)
	Nocturnal noise pollution	Disrupts sleep quality and circadian stability High cortisol Circadian clock genes dysregulation	Inflammation and oxidative stress DNA methylation changes	Daiber et al. (2022) Arregi et al. (2024) Hahad et al. (2025) Thacher et al. (2024)
	Alcohol/caffeine at late hours	Alters sleep timing (reduced REM) Circadian misalignment Circadian hormone profiles Melatonin suppression	Hormonal imbalance Cognitive dysfunction	Song and Walker (2023)
Older Adults/Aging	Reduced morning light exposure	Dampens SCN-driven rhythms Phase-shift Impact on hormone regulation Disrupted sleep-wake cycles		Lok et al. (2023) Barroggi Constantino et al. (2025)
	Increased sedentarism	Reduces physical Zeitgeber input	Short telomeres Cardiometabolic risk	Stenbäck et al. (2019) Leskinen et al. (2021)
	Polypharmacy/medication timing	Alters rhythmic physiology and pharmacodynamics	Chronotherapy effects altered	Pazan and Wehling (2021)
	Social isolation/retirement	Loss of social Zeitgebers	Impact the regulation of the described 13 hallmarks of aging	Kroemer et al. (2025)

In modern human societies, irregular exposure to circadian entrainers, as happens in shift work, is a common practice. This practice has been linked to the disruption of internal circadian clocks with external time cues, leading to a phenomenon known as chronic jetlag (Makris et al., 2023). This misalignment affects nearly half of the population and is associated with increased risks of cardiovascular disease, obesity, diabetes, and cancer; conditions commonly linked to aging (Covassin et al., 2016; Laermans and Depoortere, 2016; Albrecht, 2017). The disruption of circadian rhythms can induce oxidative stress in cells *via Clock* and *Bmal1*, important regulators of cellular senescence *in vivo*, a state in which the balance between ROS and antioxidants is disturbed (Yuan et al., 2017; Makris et al., 2023). At baseline levels, ROS support normal cellular functions, but when in excess, they can damage macromolecules such as lipids, DNA, and proteins (Sies, 2018), triggering cellular senescence. In this state, cells cease to proliferate and adopt a senescence-associated secretory phenotype, releasing inflammatory factors, including IL-6, TNF-α, CCL2, CXCL1, and matrix metalloproteinases (Ahmed et al., 2022; Zhang et al., 2022; Basisty et al., 2020). This phenomenon, alongside cellular senescence, contributes to *inflammaging*, a broader concept referring to the chronic, low-grade inflammation that arises with aging. Inflammaging plays a causal role in the aging process by promoting immunosenescence, mitochondrial dysfunction, and microbiome alterations (Liguori et al., 2018). However, other mechanisms, such as the dampening of the rhythmic expression of circadian genes in immune cells, may also contribute to the aging-associated inflammation (Blacher et al., 2022).

Artificial light at night (ALAN) has become pervasive in modern societies disrupting natural circadian rhythms. One well-known mechanism is its suppression of melatonin production (Lewy et al., 1980). Melatonin suppression leads to increased oxidative stress and DNA damage, mediated by pathways involving the p53 tumor suppressor and the NF-kB signaling cascade, which are implicated in cancer and metabolic disorders (Stevens and Zhu, 2015; Wang et al., 2015; Jiang et al., 2016; Stephenson et al., 2021). However, melatonin plays additional roles, acting as a scavenger of free radicals and as a chelating agent for heavy metals (Limson et al., 1998). For example, in human placental mitochondria, melatonin suppresses iron-dependent production of ROS (Milczarek et al., 2010). In addition, in a recent study, melatonin has been found to exert protective effects against hepatic fibrosis through melatonin receptor 2 activation, leading to the upregulation of *Bmal1* and antioxidant enzyme pathways (Kim and Cheon, 2024). The repetitive suppression of melatonin cycles by ALAN thus has the potential to accelerate aging through DNA damage, while melatonin supplementation has repeatedly been found to attenuate oxidative stress damage in age-related diseases like diabetes (Mohammadpour Fard et al., 2024). Interestingly, new avenues are emerging in our understanding of how ALAN contributes to other pathophysiological scenarios, including obesity, type 2 diabetes, and broader metabolic disturbances. These effects are likely mediated through alterations in appetite-regulating hormones, leading to increased food intake, a preference for energy-dense foods, and even gut dysbiosis (Vujović et al., 2022; Thaiss et al., 2014). When it comes to food preferences, eating behavior is orchestrated by metabolic, hedonic, and circadian pathways, which together regulate not only how much and what we eat, but also when we eat. Alterations in the expression of clock genes within these brain regions result in heightened dopamine release in response to high-calorie foods, thereby enhancing their rewarding properties and driving a preference for energy-dense foods during periods of circadian disruption (Bainier et al., 2017).

On the other hand, the timing of food intake is a critical factor influencing circadian rhythms. Research on time-restricted feeding (TRF) indicates that aligning eating patterns with natural circadian cycles can improve metabolic health and potentially slow age-related decline (Longo and Panda, 2016; Ezpeleta et al., 2024). In older humans (Lages et al., 2024; Ezzati et al., 2025) and rodents (Milan et al., 2024), restricting food intake to the active phase of the photoperiod improved markers of oxidative stress, suggesting that eating during the rest phase can result in increased oxidative stress damage compared to eating during the active phase. In a rodent model of liver ischemia-reperfusion, TRF for 8–10 h during the active phase, compared to 24 h food access, improved tissue regeneration, reduced pro-inflammatory (like IL-6) and augmented anti-inflammatory (like IL-10) markers, prevented ROS production and increased systemic β-hydroxybutyrate (BHB) (Ren et al., 2019). Fasting decreases glycogen stores in the liver, and cells shift from carbohydrate to lipid and ketone metabolism, increasing BHB levels and blocking the NLRP3 inflammasome (Youm et al., 2015), which is implicated in age-related functional decline (Youm et al., 2013). Conversely, TRF during the rest phase in rodents (Ye et al., 2024), and eating during the late active phase in humans (Allison et al., 2021) exacerbated systemic insulin resistance, a common feature in aged populations. Regarding the long-term effects, a study in a rodent model found that restricting feeding to the rest phase promoted hepatic lipid accumulation by suppressing hepatic miR-27b-3p, thereby enhancing PPARγ activity and upregulating CD36-mediated lipid transport into the liver (Tsurudome et al., 2022). These results suggest that lifelong exposure to misaligned eating patterns might potentially accelerate aging, but long-term experimental approaches are required to assess these changes.

Finally, the benefits of physical exercise in slowing the aging process have been extensively studied (Garatachea et al., 2015). However, exercise also elicits distinct effects depending on the time of the day, with morning exercise benefiting lipolysis, and evening exercise muscle mass (Gabriel and Zierath, 2019; Kim et al., 2023). At the molecular level in rodent models, exercise entrains the core clock by shifting expression patterns of *Per2* (Kemler et al., 2020), and can prevent oxidative damage derived from melatonin deficit or circadian disruption (Jana et al., 2020; Gu et al., 2024). In humans, it is challenging to determine the effects of exercise during normal sleep time. Rodent models of exercise during the rest phase are also limited. It has been reported that exercise during the rest phase, compared to the active phase, increases systemic energy expenditure without enhancing lipid oxidation according to the muscle transcriptome, an effect mediated by the fed status (Sato et al., 2019; Sato et al., 2022; Pendergrast et al., 2024). However, the effects of exercise during the rest phase on oxidative stress and tissue damage require further exploration.

Lifelong environmental exposures, such as light pollution, nutrition timing, and shift work, play crucial roles in shaping circadian health. Understanding the circadian molecular and physiological impacts of these factors can offer valuable insights into the mechanisms of aging. While targeting lifestyle and chrono-exposome factors holds promise for promoting healthy aging, the bidirectional relationship between circadian disruption and aging remains incompletely understood. To bridge this gap, there is an urgent need for both preclinical and clinical studies that clarify how environmental timing influences age-related decline. Complementing this environmental perspective, insights from biological extremes, such as premature aging syndromes and exceptionally long-lived species, offer powerful models to dissect the resilience or vulnerability of the circadian system in aging.

The molecular architecture of the circadian clock is a deep-rooted legacy of life’s earliest adaptation to Earth’s rotation. Organisms began developing intrinsic timekeeping mechanisms over 2.5 billion years ago, with cyanobacteria evolving the KaiABC protein clock system, that regulated global gene expression in alignment with day/night cycles (Swan et al., 2018; Jabbur and Johnson, 2022). In animals, this ancestral timekeeping framework evolved into transcription–translation feedback loops involving core genes like *Clock, Bmal1, Per*, and *Cry*, which share homology with ancient microbial photoreceptors and transcription factors (Stanton et al., 2022). This remarkable evolutionary continuity underscores the fundamental role of circadian timing in coordinating whole body physiology including metabolic homeostasis, DNA repair, and protein quality control, key processes implicated in aging (López-Otín et al., 2023a). By situating premature aging syndromes and long-lived species within this evolutionary framework, we highlight how these extreme models can reveal whether circadian resilience or vulnerability meaningfully influences longevity. However, it is remarkable how little primary evidence exists regarding circadian system regulation in these species and, consequently, about its potential relationship with their long or short lifespan. In the following sections, we summarize the limited literature available on their circadian systems and propose possible correlations between circadian regulatory pathways and aging, with the aim of opening new avenues for future research.

## 4 Progeroid syndromes and circadian system interactions

Aging studies have explored the major regulatory pathways involved in this complex process. *The Hallmarks of Aging,* published in 2013 by López-Otín and colleagues, and subsequently updated into their “expanding universe” a decade later, has ended on 14 hallmarks of aging providing an overview of the key biological features that influence aging (López-Otín et al., 2013; López-Otín et al., 2023b; Kroemer et al., 2025). Further attempts to understand the intricate connections between aging and the circadian system were reviewed in *Impact of the Circadian Clock on the Aging Process* by Fonseca Costa and Ripperger in 2015 and later by Welz and Aznar-Benitah (Fonseca Costa and Ripperger, 2015; Welz and Benitah, 2020). Premature aging syndromes, such as Hutchinson-Gilford progeria syndrome (HGPS), or other progeroid syndromes including Werner syndrome or Néstor-Guillermo progeria syndrome, offer unique windows into the mechanisms of accelerated aging. They serve as valuable models for exploring the interplay between aging and circadian regulation, precisely at the moment when both times meets, out of time.

Hutchinson-Gilford progeria syndrome is a premature aging disorder caused by mutations in the *LMNA* gene, that produce a defective protein called progerin leading to a disorganized nuclear architecture (Eriksson et al., 2003). This syndrome is characterized by accelerated aging, with affected individuals displaying features such as hair loss, joint abnormalities and cardiovascular disease, with a reduced lifespan. Research into HGPS hallmarks could help explore the potential role of circadian dysfunction in its pathology and identify crosstalk between both biological time systems. Genomic instability, a hallmark of HGPS, may be worsened by circadian disruption of DNA repair pathways involving *Sirt6* and *Bmal1* through nicotinamide adenine dinucleotide (NAD^+^), a seminal metabolite in aging and circadian system regulation (Kolinjivadi et al., 2021; Toiber et al., 2013; Nakahata et al., 2009). Similarly, the epigenetic alterations observed in HGPS, including loss of heterochromatin and aberrant histone modifications, likely impair the circadian regulation of chromatin accessibility and gene expression (Oh et al., 2019). The disorganized nuclear architecture in HGPS, driven by progerin, may interfere with the spatial organization of circadian gene loci, compromising rhythmic transcription. Studies revealed that CLOCK formed complexes with nuclear lamina proteins and KAP1, thus maintaining heterochromatin architecture and stabilizing repetitive genomic sequences (Liang et al., 2021). Mitochondrial dysfunction and oxidative stress, common in HGPS cells, could be exacerbated by disruption of circadian control over mitochondrial dynamics and metabolism via NAD + -dependent pathways. Loss of proteostasis, due to impaired autophagy and accumulation of misfolded proteins, may also reflect a breakdown in circadian regulation of cellular quality control systems. Proteostasis, under the regulation of the circadian system (via mTOR, and proteasome activity) (Juste et al., 2021) reciprocally induces the degradation of core circadian proteins like BMAL1, contributing to age-associated circadian disruptions and accelerated aging phenotypes (Lipton et al., 2017; Khapre et al., 2014a). Additionally, stem cell exhaustion and premature cellular senescence in HGPS resemble age-associated decline in circadian coordination of stem cell renewal and senescence-associated gene expression. Vascular dysfunction, a critical cause of morbidity in HGPS, is linked to circadian regulation of endothelial tone and inflammation (Kunieda et al., 2008). Together, these features underscore a possible bidirectional relationship between nuclear envelope defects and circadian misalignment in the pathogenesis of HGPS.

Other progeroid syndromes, while mechanistically distinct from HGPS, may also involve circadian alterations. In Néstor–Guillermo Progeria Syndrome (NGPS), caused by mutations in *BANF1* (encoding BAF1), loss of BAF disrupts nuclear architecture and chromatin organization without progerin accumulation (Cabanillas et al., 2011). Although circadian rhythms have not been directly studied in NGPS, BAF’s interaction with MAN1, a nuclear envelope protein that binds the BMAL1 promoter, and its role in chromatin tethering suggest potential circadian disruption (Zhang et al., 2015; Brunet et al., 2019). Similar gaps exist in Werner syndrome, a segmental progeria caused by mutations in WRN, a gene involved in DNA repair, telomere maintenance, and epigenetic stability (Milosic et al., 2024). While direct links between *WRN* and core circadian genes remain elusive, overlapping pathways such as chromatin remodeling, metabolic regulation, and epigenetic modifications, all influenced by circadian clocks, indicate possible crosstalk (Bellet and Sassone-Corsi, 2010; Koike et al., 2012; Chang and Guarente, 2013; Pacheco-Bernal et al., 2019; Acosta-Rodríguez et al., 2022).

Collectively, these observations point to a potential bidirectional relationship among nuclear envelope defects, circadian misalignment, and the accelerated aging phenotypes observed in progeroid syndromes, highlighting the need for direct studies on circadian system integrity in these conditions.

## 5 Circadian rhythms and long-lived animals

The evolutionary strategies that long-lived species have developed to couple aging with circadian clock homeostasis could shed light on the interaction between biological processes. Although this remains a complex and somewhat controversial area of study, many aged animal tissues exhibit dampened rhythms characterized by reduced amplitude, increased fragmentation, and impaired stability. Consequently, the expression of genes under circadian control, including those involved in metabolism, is also affected (Nakamura et al., 2016; Wallace et al., 2020; Cai et al., 2023; Masuda et al., 2023; Wolff et al., 2023; Buijink et al., 2024). Understanding whether rhythms in long-lived animals maintain their amplitude and acrophase, and how they achieve this, could help unravel the relationship between aging and the temporal dynamics of various biological pathways. Also, species adapted to extreme photoperiods demonstrate a high degree of behavioral plasticity and not only reveal how circadian rhythms adjust to challenging environments but also provide insights into the resilience of the biological clock under environmental stress, identifying mechanisms that promote greater circadian stability and, consequently, help delay aging. Since the circadian system can be entrained and stabilized by external cues, this field offers a valuable opportunity to explore new avenues for understanding the aging process. In this section, we will explore the strategies and evolutionary insights of long-lived animals such as the naked mole-rat, cavefish, and whales, to open new perspectives in the chrono-aging process.

### 5.1 The naked mole-rat

The naked mole-rat (*H. glaber*), a small rodent that strictly inhabits subterranean life, is known for its exceptional lifespan of up to 37 years, making it the longest-lived rodent species (Jarvis, 1981; Bennett and Faulkes, 2000; Buffenstein, 2005). This extraordinary animal with no age-related increase in mortality risk and negligible senescence, exhibits high fertility while maintaining proteostasis, genomic stability, resistance to cancer, and good cardiovascular, neuronal, and metabolic health, even in old age (Buffenstein, 2008; Park et al., 2008; Liang et al., 2010; Edrey et al., 2011; Ruby et al., 2018; Seluanov et al., 2018; Shepard and Kissil, 2020; Hadi et al., 2021; Oka et al., 2023).

This rodent has developed several morphological and physiological adaptations to live in complete darkness, including insensible eyes to light, small pupils with no pupillary response, and a thin optic tract, making it independent of the external light (Peichl et al., 2004; Buffenstein, 2005; Crish et al., 2006). This fact is consistent with the low expression of the *c-fos* gene in the SCN, in contrast to what is observed in animals with light-sensitive eyes and well-developed retinas. In addition, the melatonin pathway is impaired due to pineal atrophy, low or undetectable expression of genes involved in melatonin synthesis, and the presence of non-functional melatonin receptors (Kim et al., 2011; Moqrich, 2014).

Nonetheless, due to the high evolutionary conservation of the molecular clock, comparative analyses between the naked mole-rat and mice revealed that all major clock genes (*Bmal1, Clock, Per1/2, Cry1/2, Rev-Erbα/β*, and *Ror-s*) are present in the naked mole-rat’s reference genome (http://www.naked-mole-rat.org/), and are expressed in liver tissue, indicating that its circadian clock remains functional (Ghosh et al., 2021). However, although both species display rhythmic gene expression, their temporal patterns are not aligned, suggesting that the regulation of core clock genes may have evolved under distinct phase rules compared to mice, possibly due to internal factors or because the primary entrainer is something other than light.

Mitochondrial dysfunction and deregulated nutrient sensing are central to aging (López-Otín et al., 2013), with growing evidence of a bidirectional regulation between the circadian clock and metabolic pathways, particularly glucose metabolism and mTOR signaling (Khapre et al., 2014a; Khapre et al., 2014b). *Bmal1* influences insulin signaling and glucose homeostasis (Mauvoisin et al., 2014) and has been identified among 12 key longevity-associated genes in long-lived species (Yu et al., 2021). mTOR, in turn, regulates *Bmal1* expression, and disruption of this feedback loop during aging may impair metabolic control, weaken circadian robustness, and accelerate aging (Lipton et al., 2017; Cao, 2018; Ramanathan et al., 2018). Notably, these pathways are differentially expressed across aging in naked mole-rats. The expression of glycolytic and gluconeogenic enzymes is highly synchronized with the circadian molecular clock, whereas in mice, these rhythms are less coordinated. This supports the idea that naked mole-rats may have evolved more precise temporal regulation and more efficient metabolic control mechanisms adapted to their unique subterranean lifestyle (Ghosh et al., 2021). This robustness of glucose metabolism coincides with an increase in mTORC2 activity in naked mole-rats. Moreover, it has been shown a reduction in mTORC1 activity, combined with the enhanced synchronization of enzymes involved in glucose homeostasis. It has been proposed that the suppression of mTORC1 activity extends lifespan in multiple species, including mice, and that mTORC1 is one of the main drivers of aging and age-related diseases (Harrison et al., 2010; Miller et al., 2011; Ferrara-Romeo et al., 2020). In contrast, the role of mTORC2 in aging remains less defined, with studies showing that reduced mTORC2 activity shortens lifespan in mice (Nojima et al., 2013; Chellappa et al., 2019). When mTORC1 is suppressed, compensatory mTORC2 upregulation can maintain glucose homeostasis (Hagiwara et al., 2012) and may contribute to lifespan extension in rodents (Dominick et al., 2015). Notably, the elevated mTORC2 activity in naked mole-rats may support both tightly synchronized glucose metabolism and their exceptional longevity despite their small body size.

Thus, proper modulation of glucose and mTORC1-2 pathways suggests that it may enhance the robustness of circadian rhythms, as happens in the naked mole-rat and slow down the aging process by improving metabolic health and reducing cellular damage.

### 5.2 Cavefish

Subterranean environments are unique ecological systems characterized by the absence of light, high humidity, and constant temperature, resulting in a highly stable microclimate (Biswas, 2010; Culver, 2014; Lunghi et al., 2015; Culver and Pipan, 2019; Mammola, 2019). When surface-dwelling species colonize these environments, they often undergo phenotypic changes (Bilandžija et al., 2020; Lunghi and Zhao, 2020) such as loss of pigmentation and eye degeneration (Howarth and Moldovan, 2018), along with other adaptive traits like slower growth, reduced metabolic rate, and decreased investment in reproduction, all of which have been linked to increased lifespan, as in cavefish that live three times longer than surface fish populations (Poulson, 1963; Flatt and Schmidt, 2009).

The circadian system in cavefish also displays unique features that may be related to their increased lifespan (Voituron et al., 2011; Lunghi and Bilandžija, 2022). In these fishes, circadian rhythms are suppressed in their natural habitat due to the absence of light, but they can be restored under artificial light-dark cycles (Carlson and Gross, 2018) or through other environmental synchronizing factors (Yoshizawa et al., 2010; Moran et al., 2014; Blin et al., 2020; De Souza et al., 2024), taking advantage of the enhanced sensitivity of their mechanosensory and chemosensory systems (Bilandžija et al., 2012; Jeffery, 2009; Gonzalez et al., 2018). In these species, the synchronization of circadian rhythms may be influenced by other external zeitgebers, due to the interaction between the internal biological clock and associative memory through time–place learning (Mulder et al., 2013).

In *A. mexicanus*, one of the most widely used model species in cave biology, studies during the embryonic stage have shown that light-induced activation of the molecular clock genes *Cry1* and *Per2* is delayed in cave-dwelling populations compared to their surface counterparts (Frøland Steindal et al., 2018). In adults, these genes are still present but show significantly higher baseline expression levels than in surface fish, even without light exposure. This suggests that the core circadian clock mechanism in this species may be suppressed in response to an overactivation of the light input pathway and the systems responsible for clock synchronization, as part of the fish’s adaptation to the absence of light (Beale et al., 2013). The elevated baseline expression levels of *Per2* in cave-dwelling *Astyanax mexicanus* may also represent an adaptive advantage, as they trigger significantly higher expression of genes such as *CPD/PHR* and *DDB2*, which encode DNA repair proteins, helping to reduce the likelihood of harmful mutations induced by light. Interestingly, after UV exposure, cavefish show significantly less DNA damage and therefore greater repair activity compared to their surface-dwelling counterparts.

The elevated baseline expression of *Per2* in cave-dwelling *A. mexicanus* may represent an adaptive advantage, as it drives significantly higher expression of genes such as *CPD/PHR* and *DDB2*, which encode DNA repair proteins (Tamai et al., 2004; Gavriouchkina et al., 2010; Weger et al., 2011; Beale et al., 2013), thereby reducing the likelihood of light-induced harmful mutations. Notably, after UV exposure, cavefish exhibit significantly less DNA damage and greater repair activity than their surface-dwelling counterparts (Beale et al., 2013), maintaining a higher genome stability, delaying one of the main hallmarks of aging (López-Otín et al., 2023a).

### 5.3 Whales

The bowhead whale (*Balaena mysticetus*) is notable for its exceptional longevity, with a lifespan exceeding 200 years (Seim et al., 2014; Keane et al., 2015). Among the molecular mechanisms underlying this remarkable lifespan, the circadian system emerges as a critical component. In fact, *Bmal1* gene has been identified as one of the key genes associated with longevity (Yu et al., 2021). Its significance lies in its involvement in essential processes such as DNA repair, immune system regulation, and glucose signaling via the PI3K-AKT pathway, mechanisms implicated in cancer prevention and lifespan extension (Zeng et al., 2010; Beker et al., 2019; Zhang et al., 2023; Wang J. et al., 2025). Moreover, the evolutionary rate of Bmal1 has been shown to correlate with the maximum lifespan across species, suggesting that this gene is linked both to rapid evolutionary processes and to those that promote a longer life (Yu et al., 2021). In long-lived cetaceans such as the bowhead whale and the humpback whale, Yin et al. observed that approximately 50% of the circadian genes analyzed had undergone accelerated evolution, and more than 60% exhibited species-specific mutations within their functional domains. This suggests strong selective pressure on this regulatory network, potentially to support adaptations such as their characteristic sleep patterns (Yin et al., 2024).

An example of a circadian adaptation is the *FBXL21* gene, which in cetaceans more efficiently promotes the degradation of the CRY1 protein. This facilitates whales’ ability to maintain prolonged wakefulness in one cerebral hemisphere during unihemispheric sleep, providing them with behavioral flexibility and sustained alertness in the marine environment (Yin et al., 2024), factors that directly contribute to their survival and potential for longevity. Subsequent experiments in zebrafish have validated this regulation of daytime cytoplasmic accumulation of CRY proteins by the functional variant of *FBXL21* found in cetaceans (Hirano et al., 2013). Another adaptation that allows for more flexible control of biological rhythms is a specific mutation identified in the *NFIL3* gene. Although *NFIL3* normally functions as a transcriptional repressor of key circadian rhythm genes, in cetaceans its efficiency in repressing target genes is reduced, and its likelihood of degradation is increased (Yin et al., 2024). The accumulation of mutations in other core clock genes such as *Clock* and *DEC2* (Yin et al., 2024) further suggests an evolutionary convergence in the reconfiguration of the circadian system. This may reflect an adaptation toward slower or more controlled aging in these species by optimizing cellular, endocrine, and metabolic cycles involved in senescence.

Additionally, genes related to the insulin signaling pathway and immune response have been shown to be closely linked to longevity (Yu et al., 2021). In particular, the expression of the insulin receptor protein, which regulates energy metabolism, has been positively correlated with mammalian longevity (Ma S. et al., 2016). Furthermore, insulin pathway-dependent proteinss such as mTOR, AKT, and PI3K are associated with metabolic homeostasis, cell cycle regulation, proliferation, cancer, and longevity (Veilleux et al., 2010; Kenyon, 2011; Xie et al., 2019; Ramasubbu and Devi Rajeswari, 2023). Moreover, several genes undergoing accelerated evolution or positive selection in whales and other long-lived species are involved in the insulin/IGF-1 signaling pathway, reinforcing the idea that this pathway plays a key role in lifespan extension.

Behavioral plasticity resulting from these genetic modifications represents a key adaptive advantage that likely contributes to the longevity of these marine mammals by enabling more refined control of their metabolism, neuronal activity, and repair mechanisms. Thus, the modified circadian system of whales may function as a central regulator of the aging rate, integrating environmental, physiological, and behavioral signals.

**FIGURE 4:**
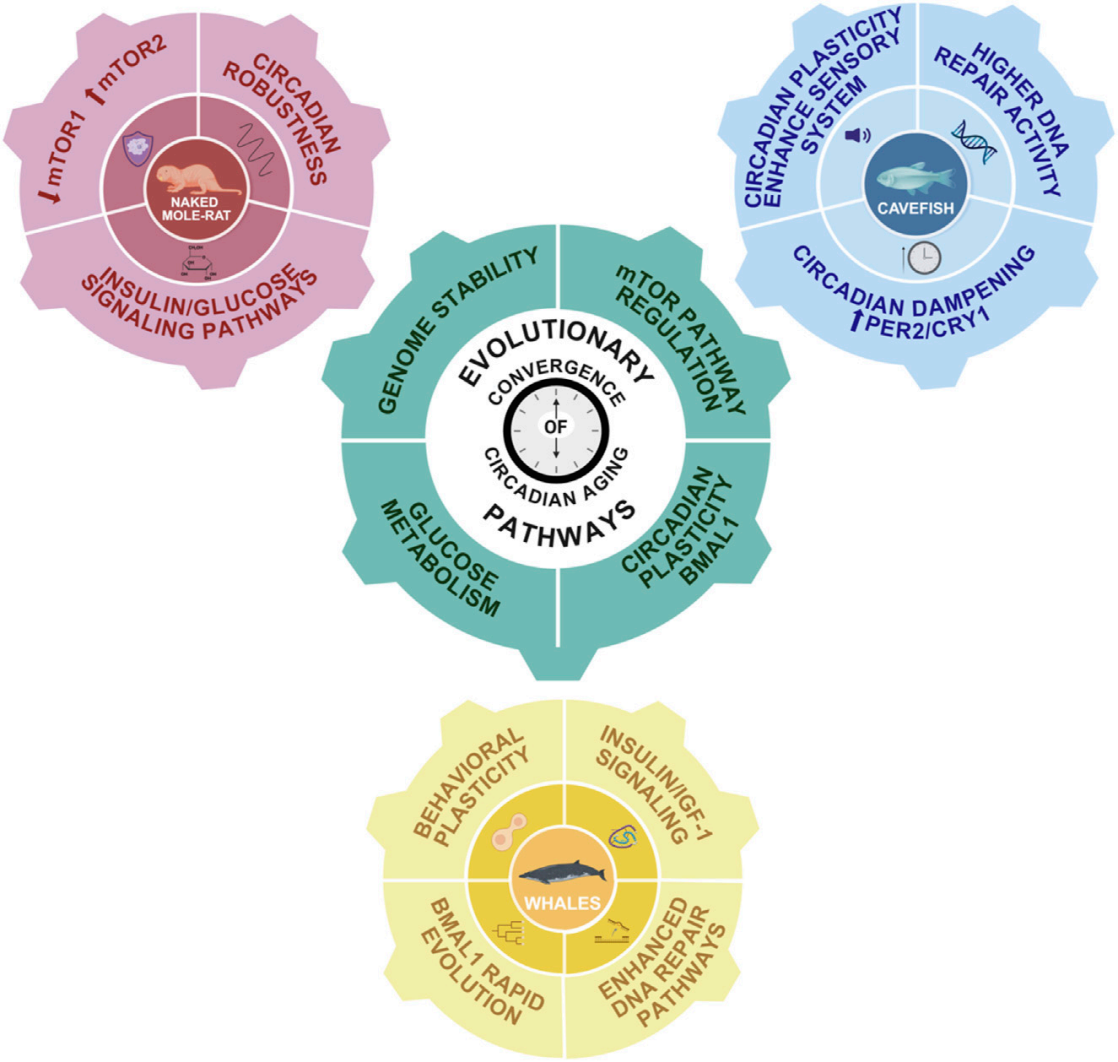
Evolutionary convergence of circadian-aging pathways. Schematic representation of the main interconnected pathways linking aging and the circadian system in long-lived species. In pink, the naked mole-rat exhibits a robust insulin/glucose signaling pathway, downregulated mTORC1 and upregulated mTORC2 pathways, and a strong circadian system. In blue, cavefish display enhanced DNA repair activity and dampened circadian rhythms due to constant external conditions. However, their circadian system remains highly plastic, likely due to an enhanced sensory system. In yellow, whales exhibit increased DNA repair capacity, robust PI3K/AKT and insulin/IGF-1 signaling pathways, and high behavioral plasticity associated with rapid Bmal1 evolution. The central gear symbolizes conserved mechanisms potentially involved in lifespan extension and regulated by the circadian system: insulin/IGF-1 signaling, glucose metabolism, and mTOR pathway modulation.

In summary, studies across highly divergent long-lived species such as whales, cavefish, and naked mole-rats, have revealed that a common molecular thread linking the circadian system to extended lifespan is the regulation of insulin/IGF-1 signaling and glucose metabolism. This pathway, tightly controlled by core clock components like *Bmal1*, appears to be consistently optimized to enhance metabolic efficiency, preserve energy homeostasis, and reduce age-related cellular damage. In whales, positive selection of insulin-related genes and circadian regulators supports metabolic balance and cancer resistance; in cavefish, adaptations to lightless environments result in altered circadian gene expression that may indirectly stabilize energy use and DNA repair; and in naked mole-rats, precise circadian coordination of glucose metabolism and distinct mTORC1/2 activity likely contribute to their exceptional longevity and negligible senescence. Together, these findings suggest that the evolutionary fine-tuning of circadian control over metabolic pathways, genome stability, and circadian plasticity by *Bmal1* may be a unifying strategy for lifespan extension across distant taxa (Figure 4).

## 6 Chronodisruption and age-related diseases

Insights from long-lived species offer a valuable evolutionary perspective on how the circadian system may be optimized to promote resilience against aging and disease. A recurrent feature in these organisms is the enhanced coupling between the molecular clock and key metabolic pathways, including insulin/IGF-1 signaling, glucose metabolism, and mTOR regulation, which are centrally involved in human aging and its associated pathologies, as well as in maintaining DNA integrity. In contrast, humans exhibit a progressive erosion of circadian robustness with age, a decline that exacerbates the risk and severity of chronic diseases by disrupting metabolic, neuronal, and cardiovascular homeostasis. This reciprocal reinforcement between aging and chronodisruption establishes a maladaptive cycle. While circadian rhythms are not defined as a standalone hallmark of aging, their disruption intersects with multiple established hallmarks, such as chronic inflammation, mitochondrial dysfunction, epigenetic alterations, cellular senescence, and particularly psychosocial isolation, a newly proposed hallmark (Kroemer et al., 2025).

In this section, we analyze four major aging-related diseases—neurodegenerative, cardiovascular, metabolic disorders, and cancer—and explore how the interplay between linear time, represented by the aging process, and cyclical time, embodied by the circadian system, shapes their pathophysiology. Our main goal is to identify the common pathways shared by these two processes, which could then be studied in depth and targeted in future circadian aging therapies (Table 2).

**TABLE 2:** Age-related diseases and their intersection with the circadian system.

Age-related disease		Circadian disruption association	Aging-like related outcomes	Specie	References
Neurodegenerative diseases	Physiological changes	Sleep fragmentation, reduced sleep quality and reduced activation of glymphatic system SCN desynchronization Melatonin disturbances Reduce of clock genes expression	Reduced cortical excitability and cognitive decline Loss of GABAergic neurons Increased Aβ production and decreased Aβ clearance White matter reduced volume and myelin dysfunction Axonal degeneration, astrocytic activation, functional disfunction	Mouse and humans	Videnovic and Zee (2015) Kang et al. (2009) Ma Z. et al. (2016) Musiek and Holtzman (2016) Lananna and Musiek (2020) Saeed and Abbott (2017)
	Genetic alterations	BMAL ↓ mean expression in extra-SCN brain (hippocampus, cortex); age-dependent attenuation; loss/dampening of rhythmicity in some contexts PER1: epigenetic downregulation with age in hippocampus and altered expression PER2: damped rhythms and increased fragility *ex-vivo* in aged SCN in some studies REV-ERBα: REV-ERBα deletion/perturbation modifies daily microglial function	BMAL1 KO, premature aging: reduced lifespan, sarcopenia, cataracts, organ atrophy; aged ↓Bmal1 in hippocampus linked to impaired neurogenesis and memory-related decline Altered PER1/2 linked to impaired memory and circadian rhythmicity; SCN PER2 fragility, reduced robustness of central clock outputs (behavioral and physiological rhythm dampening) REV-ERBα deletion, increased microglial phagocytosis and synaptic loss (hippocampus) and altered complement expression, links to synaptic loss/neuroinflammation relevant to neurodegeneration/aging	Mouse (KO and aged WT); hamster; rat; human (reduced with age reported) Mouse (SCN, hippocampus), rat, human post-mortem (prefrontal cortex) Mouse (hippocampus, microglia); human associations	Kondratov et al. (2006) Nakamura et al. (2015) Kwapis et al. (2018) Griffin et al. (2020) Lee et al. (2023)
Cardiovascular diseases	Behavioral changes by shift work	Increased cardiovascular risk profile Higher prevalence of hypertension Dyslipidemia Central obesity Acute ischemic events Elevated inflammatory markers (IL-6, C reactive protein, TNF-α) Elevated lood pressure Shorten lifespan	Higher systolic pressure Lower diastolic pressure and widened aortic pulse pressure Increased collagen and decreased elastin in the aortic extracellular matrix	Humans, hamster, mice	Hemmer et al. (2021) Wong et al. (2015) Torquati et al. (2018) Penev et al. (1998) Tsioufis et al. (2008) Morris et al. (2016) North and Sinclair (2012)
	Genetic alterations	BMAL1 disfunction: impaired angiogenesis, vascular remodeling, endothelial dysfunction, heightened thrombosis risk, and reduced eNOS–Akt signaling Bmal polymorphisms are associated with hypertension BMAL1 and REV-ERBα lost of rhythm: impaired mitochondrial biogenesis and reduced cardiac functional reserve, increased risk of heart failure Clock gene disfunction impact on acute myocardial infarction incidence and extension	Hypertension Endothelial dysfunction Higher risk of heart failure	Humans, hamster, mice	Lecour et al. (2022) Takeda and Maemura (2016) Costello et al. (2023) Crnko et al. (2019) Lananna and Musiek (2020) Anea et al. (2009) Astone et al. (2023) Paschos and FitzGerald (2010) Alibhai et al. (2014) Thosar et al. (2018)
Metabolic diseases	Behavioral changes by food intake	Impaired glycemic control, as evidenced by elevated HbA1c levels Increased risk of T2D Accumulation of visceral fat Accelerated β cell failure and hyperglycemia Arhythmic secretion of insulin, incretins, and glucocorticoids			Nakajima et al. (2017) Rae et al. (2021) Xu et al. (2023) Qian and Scheer (2016) Mason et al. (2020)
	Genetic alterations	CLOCK polymorphisms: associated with obesity, hyperglycemia and higher prevalence of T2D CLOCK mutation: Pancreatic β cell failure, leading to hypo-insulinemia and hyperglycemia Bmal1, PER1/2 mutation: Glucose intolerance and hyperglycemia BMAL1 deficiency: β cell failure via oxidative stress-induced mitochondrial uncoupling BMAL1 disfunction: dyslipidemia	Increased glucose intolerance Altered glucocorticoids secretion Altered plasma adipokine levels and consecutive insulin resistance, due to adipose tissue dysfunction		Corella et al. (2016) Rakshit et al. (2014) Maury (2019) Shimba et al. (2011) Elahi et al. (2002) Lananna and Musiek (2020) Zhao and Yue (2024) Sen et al. (2023) Tran et al. (2024)
Shared mechanisms across diseases		Reduced circadian amplitude and plasticity	Reduced mitochondrial function Reduced autophagy Inflammaging Increased ROS production		Massudi et al. (2012) Imai and Guarente (2014) Yamaguchi and Otsu (2012) Palmer et al. (2025)

### 6.1 Neurodegenerative diseases

Aging is one of the main risk factors for neurodegenerative diseases, promoting their onset and progression through reduced hippocampal neurogenesis, loss of synaptic plasticity, and inflammaging (Kritsilis et al., 2018; Hou et al., 2019), which is intensified by age-related increases in astrogliosis and microglial activation (Iskusnykh et al., 2024). Postmitotic neurons accumulate signs of senescence, activating pathways that lead to progressive synaptic dysfunction and neuronal failure. The loss of the suprachiasmatic nuclei ability to resynchronize in response to environmental cues adds to these factors, reducing the amplitude and synchrony of clock genes expression in key regions such as the prefrontal cortex and hippocampus, which is associated with cognitive deficits and increased susceptibility to neurological insults (He et al., 2023).

Circadian misalignment, particularly of the sleep-wake cycle, has been implicated in the pathophysiology of neurodegenerative disorders such as Alzheimer’s, Parkinson’s, and Huntington’s diseases, acting not only as a clinical manifestation but also as an active driver of disease progression (Videnovic and Zee, 2015; Shen et al., 2023), by disrupting blood-brain barrier integrity and inducing neuroinflammation (Lananna and Musiek, 2020; Schurhoff and Toborek, 2023; Cheng et al., 2024). In Alzheimer’s disease, prolonged wakefulness promotes β-amyloid (Aβ) production and the fragmentation of 24 h activity rhythms has proven to be a strong predictor of Aβ deposition, stronger than total sleep duration (Kang et al., 2009; Ma Z. et al., 2016; Nguyen Ho et al., 2024), while deep sleep enhances its glymphatic clearance (Xie et al., 2013). In Parkinson’s disease, circadian disturbances precede motor symptoms and are associated with dopaminergic loss and degeneration of the SCN (Joyce et al., 2018; Stewart et al., 2018), while in Huntington’s disease, rhythm fragmentation and its experimental restoration directly modulate the functional and behavioral progression of the disease (Fitzgerald et al., 2023; Saade-Lemus and Videnovic, 2023).

Clock proteins have been directly implicated in neuronal homeostasis, and their dysfunction has been associated with synaptic alterations, accumulation of toxic proteins, inflammation, and neuronal death (Musiek and Holtzman, 2016; Carter et al., 2021). Aging is accompanied by marked alterations in the expression and rhythmicity of several core clock genes in the brain, with consequences that align closely with recognized hallmarks of brain aging. At the systems level, transcriptomic profiling in mouse and human brains reveals widespread loss, phase-shifting, or rewiring of diurnal gene expression programs with age, changes that correlate with impaired cognition, metabolic dysregulation, and decreased circadian robustness (Seney et al., 2019; Wolff et al., 2023; Archer et al., 2024; Ishikawa et al., 2025; Wang L. et al., 2025). In mice, *Bmal1* promotes the expression of antioxidant enzymes and plays an autonomous role in maintaining neural integrity, such that its reduction induces oxidative stress, neuroinflammation, and synaptic dysfunction independently of behavioral rhythmic changes (Griffin et al., 2020; Barone et al., 2023; Iweka et al., 2023; Kanan et al., 2024). Its expression declines in extra-suprachiasmatic- nucleus regions such as the hippocampus and cortex in aged rodents and humans, a change linked to impaired neurogenesis, memory deficits, and, when genetically ablated, premature aging phenotypes including sarcopenia, cataracts, and reduced lifespan (Kondratov et al., 2006). *Per2* rhythms in the SCN show dampened amplitude and greater fragility *ex vivo* in aged mice, while *Per1* can be epigenetically downregulated in the aged hippocampus, both changes associated with reduced robustness of circadian outputs and cognitive decline (Nakamura et al., 2015; Kwapis et al., 2018). *Rev-erbα* has shown neuroprotective properties in Parkinson’s disease, as the loss of its rhythmicity in the substantia nigra of affected patients is associated with exacerbated microglial activation and increased inflammatory markers, conditions that are attenuated following pharmacological reactivation of *Rev-erbα* (Kou et al., 2022). Its dysregulation in the aged hippocampus and microglia enhances synaptic loss and microglial phagocytosis through complement pathway upregulation, linking circadian disruption to neuroinflammation and neurodegeneration (Griffin et al., 2020; Lee et al., 2023). *Rev-erbα* In Alzheimer’s disease, altered expression of clock genes is accompanied by increased Aβ deposition, tau hyperphosphorylation, and poorer performance in memory tests in animal models (Niu et al., 2022). Additionally, the circadian clock regulates the inflammatory and stress-response systems via the hormonal axis, promoting increased cortisol secretion, which, through feedback mechanisms, destabilizes the clock itself. This cycle has been associated with early phases of neurodegeneration, preceding overt cognitive symptoms.

Thus, a vicious cycle is established in which aging, while imposing neurological changes intrinsic to advanced age and increasing susceptibility to circadian disturbances, also contributes to the worsening of chronodisruption by impairing the SCN’s ability to synchronize with environmental stimuli. *Clock* gene dysregulation, driven by both aging and circadian misalignment, exacerbates this scenario by compromising detoxification processes, immune control, synaptic stability, and the synchronization between central and peripheral brain structures.

### 6.2 Cardiovascular diseases

Aging promotes progressive structural and functional alterations in cardiovascular physiology, leading to increased arterial stiffness (De La Maza-Bustindui et al., 2025), endothelial dysfunction (Donato et al., 2018), reduced heart rate variability (Natarajan et al., 2020), and declining ventricular function (Westhoff et al., 2024), all of which are associated with the loss of cardiomyocytes, accumulation of fibrosis, and reduced sensitivity to hemodynamic stress, factors that compromise the heart’s ability to adapt to physiological demands (North and Sinclair, 2012). As aforementioned, SCN exhibits a decline in rhythmic robustness with advancing age, characterized by reduced amplitude, consistency, and synchronization of biological rhythms, which impairs synchronization between central and peripheral clocks as well as between the organism and its environment (Crnko et al., 2019). Moreover, aging is associated with inflammaging, which chronically and systemically impairs endothelial function, promotes oxidative stress, and alters vascular responses to homeostatic stimuli, thereby increasing susceptibility to cardiovascular events.

The integrity of the cardiovascular system substantially depends on the proper temporal organization of its hemodynamic, metabolic, and autonomic processes. This dependence is particularly evident in the circadian nature of cardiovascular patterns, such as the blood pressure rhythm, which displays a nocturnal dip, and in the increased incidence of critical cardiovascular events during the early morning hours. Among the physiological alterations frequently described in this context, the non-dipping blood pressure pattern is associated with increased arterial stiffness, nocturnal sympathetic overactivity, and the activation of subclinical inflammatory pathways (Tsioufis et al., 2008). This pattern represents an independent prognostic marker that demonstrates potential reversibility through chronobiological realignment interventions, suggesting a possible causal link (Uzu et al., 2006).

Echoing these physiological patterns, individuals exposed to circadian misalignment, such as night-shift workers, exhibit a significantly increased cardiovascular risk profile, with a higher prevalence of hypertension, dyslipidemia, central obesity, and acute ischemic events (Wong et al., 2015; Torquati et al., 2018; Hemmer et al., 2021). Controlled laboratory studies show that transient circadian–behavior misalignment elevates inflammatory markers (IL-6, C reactive protein, TNF-α) and blood pressure, and impairs glucose regulation, changes linked to cardiovascular risk (Morris et al., 2016). In animal models, jetlag protocols induced a shortened lifespan by 11% in cardiomyopathic hamsters and impair post–myocardial infarction recovery in mice (Penev et al., 1998; Alibhai et al., 2014; Thosar et al., 2018).

Genetic association studies further suggest that variants in the *Clock* gene may modulate cardiovascular risk in elderly individuals through interactions with behavioral factors such as dietary patterns and chronotype, underscoring the clinical relevance of the interplay between aging, circadian rhythm, and cardiometabolic health (Corella et al., 2016). The circadian machinery rhythmically regulates the expression of genes involved in cardiovascular homeostasis, including endothelial, oxidative, and metabolic functions (Takeda and Maemura, 2015; Crnko et al., 2019). *Bmal1* regulates anti-inflammatory and antioxidant pathways essential for maintaining vascular tone and preventing endothelial dysfunction. Its impairment leads to reduced expression of antioxidant enzymes, accumulation of reactive oxygen species, and disruption of nitric oxide regulation, thereby promoting atherogenesis, increasing coronary instability, and elevating the risk of acute myocardial infarction (Takeda and Maemura, 2015; Crnko et al., 2019; Costello et al., 2023). In mouse models, genetic disruption of the core clock gene *Bmal1*, global or endothelial-specific knockout, results in impaired angiogenesis, vascular remodeling, endothelial dysfunction, heightened thrombosis risk, and reduced eNOS–Akt signaling, hallmarks of vascular aging (Anea et al., 2009; Paschos and FitzGerald, 2010; Astone et al., 2023). Clock, Per2, and Cry1/Cry2 mutant mice lose normal blood pressure rhythms and develop arrhythmia, indicating the clock’s central control over cardiac physiology (Costello et al., 2023). Simultaneously, the coordinated activity of BMAL1 and REV-ERBα also controls mitochondrial genes, supporting the myocardium energetic adaptation to environmental fluctuations. Disruption of this rhythmic regulation impairs mitochondrial biogenesis and reduces cardiac functional reserve, particularly under hemodynamic stress, thus increasing the risk of heart failure (Lecour et al., 2022; Murgo et al., 2023).

At the molecular level, in mice, microRNA-29 (miR-29), which has been implicated in both aging and the metabolic regulation of cardiac function (Caravia et al., 2017; Caravia et al., 2018), emerges as a crucial node linking these processes. Notably, miR-29 has been shown to regulate the core clock gene *Per2* (Zhao et al., 2014), suggesting a bidirectional relationship between the circadian system and miR-mediated control of cardiovascular aging. This highlights an intricate regulatory triad in which circadian rhythms, cardiac metabolism, and aging processes converge through shared molecular mediators such as miR-29.

### 6.3 Metabolic diseases

Aging imposes progressive physiological changes that compromise metabolic homeostasis and increase susceptibility to the development of conditions such as insulin resistance, visceral obesity, and type 2 diabetes (T2D) (Cardinali and Hardeland, 2017; Poggiogalle et al., 2018). The reduction of metabolic flexibility, mitochondrial dysfunction, and accumulation of ectopic lipids, particularly in the liver and adipose tissue, promote a state of inflammaging, which is further aggravated by the diminished amplitude and robustness of endogenous rhythms in both the SCN and peripheral metabolic organs (Cardinali and Hardeland, 2017; Chan et al., 2022). The reduced rhythmicity of melatonin and cortisol production, combined with the fragmentation of rest-activity cycles and the decline in deep sleep among the elderly, is associated with a higher risk of metabolic dysfunction, even in individuals with protective genetic predispositions (Baron et al., 2018; Niu et al., 2022; Nguyen Ho et al., 2024).

Chronic exposure to conditions that promote circadian misalignment weakens the amplitude of circadian rhythms, disrupts the sleep-wake cycle, and facilitates the onset of the same conditions mentioned above (Zimmet et al., 2019; Makarem et al., 2021). Population-based studies conducted in diverse contexts consistently demonstrate that altered sleep patterns, both in quality and fragmentation, as well as nighttime light exposure, are independently associated with impaired glycemic control, as evidenced by elevated *HbA1c* levels, increased risk of T2D, and accumulation of visceral fat (Nakajima et al., 2017; Rae et al., 2021; Xu et al., 2023). Mechanistically, circadian misalignment impairs synchronization between the suprachiasmatic nucleus and peripheral clocks, thereby compromising the temporal secretion of insulin, incretins, and glucocorticoids, and negatively affecting both glycemic and lipid homeostasis (Qian and Scheer, 2016; Mason et al., 2020).

The CLOCK:BMAL1 complex activates the transcription of genes encoding proteins involved in glucose uptake and processing, mitochondrial metabolism, and lipolysis, thus linking the circadian clock to energy metabolism (Maury, 2019; Petrenko et al., 2023). In this context, BMAL1 deficiency induces desynchronized expression of gluconeogenic genes and suppresses transcriptional rhythms of mitochondrial biogenesis, resulting in fasting hyperglycemia and insulin resistance. This resistance is further exacerbated by reduced ATP production, accumulation of reactive oxygen species (ROS), and activation of subclinical inflammatory pathways (Rakshit et al., 2014; Qian and Scheer, 2016; Peek, 2020). In adipose tissue, alterations in PER2 impair the rhythmic secretion of leptin and adiponectin, while dysfunction of REV-ERBα compromises the repression of lipogenic and inflammatory genes, thereby intensifying visceral fat accumulation and promoting chronic low-grade inflammation (Szewczyk-Golec et al., 2015; Cardinali and Hardeland, 2017). Dietary lipids act as epigenetic modulators of core clock genes through microRNA regulation, altering the temporal regulation of glycolytic and lipogenic pathways (Altman et al., 2012). Additionally, hormonal factors such as incretin peptides interact with peripheral clock genes, modulating, among others, the expression of BMAL1 and PER2 in tissues such as the liver and pancreas, thus establishing a bidirectional link between postprandial metabolism and molecular rhythmicity (Petrenko et al., 2023; Zilstorff et al., 2024).

Thus, aging, by reducing rhythm robustness and metabolic flexibility, weakens the organism’s temporal adaptation mechanisms and increases its vulnerability to external factors that induce circadian misalignment, while chronodisruption itself exacerbates this susceptibility, creating a self-reinforcing loop. In parallel, the dysregulation of clock genes compromises the temporal expression of metabolic genes, completing the chain of events leading to a collapse in the temporal organization of physiological processes and triggering a progressive dysfunctional metabolic state. Therefore, the integration of aging, chronodisruption, and molecular clock dysregulation constitutes a central pathogenic axis in the onset and perpetuation of metabolic diseases.

### 6.4 Cancer

Aging is one of the major risk factors for cancer development (Peterson and Kennedy, 1979; López-Otín et al., 2023b). Moreover, aging and cancer share features such as genomic instability, epigenetic alterations, chronic inflammation, and cellular senescence (López-Otín et al., 2023a; López-Otín et al., 2023b), which either directly impact circadian rhythm function or are themselves regulated by these rhythms.

Furthermore, disruption of circadian rhythms contributes to cancer development and progression, likely due to their regulatory role in sleep, immune function, metabolism or genome integrity (Savvidis and Koutsilieris, 2012). In this context, studies in rats have shown that circadian disruption caused by constant light exposure leads to accelerated aging, a significant reduction in lifespan, and the rapid development of spontaneous tumors, including carcinomas, hematologic malignancies, and tumors of the reproductive organs (Vinogradova et al., 2007; Vinogradova et al., 2009; Vinogradova et al., 2010; Anisimov et al., 2013). Also, rodents subjected to jetlag conditions exhibited accelerated tumor growth, increased metastasis, and impaired antitumor immune responses (Filipski and Lévi, 2009; Roberts et al., 2022). One proposed mechanism involves the decreased levels of two antioxidant enzymes, superoxide dismutase and catalase (Bartsch, 2010), which exposes cells to excessive oxidative stress, thereby accelerating aging and increasing cancer risk. Furthermore, alterations in feeding-fasting patterns induced by circadian disruption promote carcinogenic processes by abolishing the temporal expression of genes involved in metabolic and immune pathways and by amplifying a pro-inflammatory microenvironment conducive to tumor progression (Crespo et al., 2025). Mechanistically, irregular meals reprogram liver and adipose clocks and their output pathways (e.g., AMPK–SIRT1-dowregulated-, mTOR–SREBP-upregulated-, also important pathways in aging), uncoupling peripheral oscillators from the SCN and abolishing temporal segregation of anabolism, repair, and immune surveillance (Guan et al., 2020; Weger et al., 2021; Acosta-Rodríguez et al., 2024). These effects sit on the global, genome-scale architecture of the clock defined by Takahashi and colleagues, who showed pervasive circadian control of transcription factor occupancy, RNAPII recruitment, and chromatin state that links clock output to metabolism and cell growth (Koike et al., 2012). Time restricted feeding to the active phase restores rhythmic gene expression and metabolic flexibility and, in preclinical models, slows tumor growth and metastasis in breast cancer settings (Das et al., 2023).

In humans, chronic sleep deprivation, insomnia, and shift work have been associated with an elevated risk of breast (Salamanca-Fernández et al., 2018; Wei et al., 2022), prostate (Salamanca-Fernández et al., 2018), and colorectal cancer (Garcia-Saenz et al., 2020; Chiang et al., 2023). Moreover, in 2007 the International Agency for Research on Cancer (IARC) classified circadian disruption as a probable human carcinogen (Group 2A), based on the increased cancer susceptibility observed in shift workers (Straif et al., 2007). Accordingly, various studies have shown that alterations in genes such as *Bmal1*, *Clock*, *Per1/2*, or *Cry1/2* (Hoffman et al., 2010; Yu et al., 2013; Gong et al., 2021; Jiang H. et al., 2021; Santoni et al., 2023; Zheng et al., 2024) can increase the likelihood of tumor initiation, proliferation, invasion, migration, and progression in multiple cancer types (Sulli et al., 2019; Sancar and Van Gelder, 2021), including breast cancer (Wang et al., 2019), colorectal cancer (Sakamoto and Takenoshita, 2015), hepatocellular carcinoma (Yang et al., 2022), melanoma (Zhang et al., 2024), and ovarian cancer (Sun et al., 2017). Often, these variations are limited to single nucleotide polymorphisms (SNPs) in core clock genes (Zienolddiny et al., 2013; Chen et al., 2019).

As we age, the synchronization between the central biological clock and peripheral clocks shifts (Patke et al., 2020), leading to impaired bodily functions and the onset of diseases, including tumors (Roenneberg and Merrow, 2016; Welz and Benitah, 2020). For instance, aging and its associated disruption of circadian rhythms can result in altered secretion patterns of hormones such as melatonin (Karasek, 2004), diminishing its antitumor effects due to its antioxidant and immunomodulatory properties (Bonmati-Carrion and Tomas-Loba, 2021), and potentially facilitating the growth and metastasis of cancer cells (Jung-Hynes et al., 2010).

At the molecular level, *Bmal1* stands out as a central regulator of genomic surveillance, preventing the replication of DNA-damaged cells through its transcriptional modulation of repair genes, activation of p53, and the imposition of rhythmicity on checkpoint mechanisms (Kiessling et al., 2017; De Assis et al., 2018). Members of the *Per* gene family, in turn, negatively regulate the oncogene *MYC* and stabilize the checkpoint protein Chk2 (Collis and Boulton, 2007; Wang C. et al., 2025). *Bmal1* deficiency can lead to genomic instability, increased oxidative stress, and cell cycle imbalance, thereby promoting cancer development and accelerating aging through its capacity to enhance mTORC1 activity (Khapre et al., 2014a). Similarly, loss of *Per2* can result in increased cellular proliferation and reduced inhibitory regulation of the mTORC1 complex (Wu et al., 2019).

Furthermore, with age, the efficiency of the DNA Damage Response in eukaryotes, which is responsible for preventing the replication of damaged DNA, also declines (Zhu et al., 2024). This network includes numerous genes that exhibit circadian rhythms (Wang et al., 2017) or directly interact with components of the molecular clock, and their progressive loss of functionality contributes both to circadian disruptions and to increased accumulation of genomic damage in cells, thereby elevating the risk of cancer development (Miller et al., 2021).

Disrupted nutrient sensing is also a common feature of both aging (López-Otín et al., 2023a) and cancer (Hanahan, 2022), with its physiological regulation closely linked to circadian functionality (Cao and Wang, 2017; Verlande and Masri, 2019). This occurs through enzymes such as SIRT1, which can interact with molecular clock proteins like CLOCK and PER2, affecting their acetylation status (Asher et al., 2008; Nakahata et al., 2008), or through the protein kinase mTOR, whose activity exhibits rhythmicity (Khapre et al., 2014b) and plays a key role in both aging and tumorigenesis (Weichhart, 2018; López-Otín et al., 2023a; Mehta et al., 2024).

Circadian disruption emerges, based on current evidence, not merely as a risk factor, but as a causal agent in the initiation and progression of various cancers. Aging exacerbates this scenario by weakening circadian resilience mechanisms and antitumor defenses.

## 7 Chronotherapy and aging

Pharmacotherapy, traditionally based on standardized dosing protocols, presents considerable challenges when applied to the geriatric population. Older adults exhibit specific physiological changes that increase their vulnerability to adverse drug reactions and toxicity, forming the foundation of geriatric pharmacology for decades. However, emerging evidence highlights that circadian dysregulation with age adds a crucial, often overlooked dimension to this vulnerability. The progressive deterioration of the suprachiasmatic nucleus impairs responsiveness to environmental cues, contributing to internal desynchrony and promoting a breakdown in physiological homeostasis. This circadian misalignment impacts key processes, including sleep-wake cycles, metabolic regulation, immune responses, and cognitive resilience.

Notably, psychosocial isolation, which increases with age (Dahlberg et al., 2024), compounds circadian disruption by weakening social and environmental zeitgebers, activating neuroendocrine stress responses and sleep disturbances (Stafford et al., 2013; Meyer et al., 2024; Yeom et al., 2024; Liu and Jiang, 2025). This establishes a vicious cycle in which aging and chronodisruption reinforce each other, accelerating biological decline and disease susceptibility (Kroemer et al., 2025). Given that circadian rhythms modulate both pharmacokinetics and pharmacodynamics, governing drug absorption, distribution, metabolism, and elimination, understanding the chronobiological underpinnings of organ function becomes essential to optimize pharmacological interventions in aging individuals.

Integrating circadian biology into clinical practice thus adds a multidirectional perspective: the circadian system influences aging, is influenced by it, and critically modulates therapeutic efficacy and safety. As such, preserving circadian integrity emerges as a strategic target in precision geromedicine, not only to mitigate systemic aging but also to tailor pharmacological treatments.

On one hand, it is important to know the circadian physiological detoxification ratio to understand pharmacokinetics. In this regard, precision geromedicine should take into account that the expression of metabolizing enzymes (such as cytochrome P450 isoforms) and hepatic membrane transporters are regulated, like many other genes, by clock-dependent mechanisms, and influence intestinal absorption, hepatic uptake, and biliary and renal excretion of various drugs (Zhang et al., 2009; Ohdo, 2010; Dallmann et al., 2016; Kobuchi et al., 2018; Mukherji et al., 2019; Ayyar and Sukumaran, 2021; Geng et al., 2021; Ma et al., 2023). The alteration of these mechanisms generates distinct temporal windows of bioactivation, efficacy, and toxicity, even under physiological conditions. In addition to this molecular regulation, pharmacokinetics is also influenced by circadian variation in physiological processes such as hepatic blood flow, gastrointestinal motility, gastric pH, renal perfusion, and plasma concentration of binding proteins, which vary rhythmically and interfere with the absorption, availability, metabolism, and excretion of drugs.

However, during aging, a number of molecular, structural, especially in the suprachiasmatic nucleus, and physiological alterations occur (Hood and Amir, 2017; Buijink and Michel, 2021), leading to reduced clock gene expression, particularly in hepatic, intestinal, and renal tissues (Nakamura et al., 2011; Kobuchi et al., 2018; Xu et al., 2023). As a result, the circadian expression of metabolizing enzymes becomes erratic, such as carboxylesterase-1, essential for prodrug bioactivation and hepatic detoxification, affecting drug clearance (Dallmann et al., 2016; Ballesta et al., 2017; Ma et al., 2023). NAD^+^ metabolism is another molecular axis disrupted during aging, impairing hepatic circadian rhythms through three pathways: by reducing sirtuin activity, the function of the CLOCK:BMAL1 complex, and the acetylation of protein targets, not only in the liver, but also in the intestine and the central nervous system. In this way, changes in NAD^+^ metabolism also promote generalized peripheral desynchronization (Massudi et al., 2012; Gomes et al., 2013; Imai and Guarente, 2014).

In addition to these molecular and epigenetic changes, anatomical and functional alterations characteristic of older adults negatively influence pharmacokinetics in this population. A reduction in hepatic blood flow, renal function, and gastric pH, as previously mentioned, compromises drug excretion, while the loss of lean mass and increased body fat alter drug distribution, aggravating the effects of chronodisruption (Hämmerlein et al., 1998; Baraldo and Furlanut, 2006; ELDesoky, 2007).

All these alterations contribute not only to the weakening of the response to conventional therapy in this population, but also to chronotherapy, which presupposes a physiological rhythmicity that is not observed in these patients, particularly in the presence of comorbidities, polypharmacy, or sleep disorders (Lévi et al., 2010; Ohdo, 2010; Dallmann et al., 2016). A paradigmatic example is the nighttime administration of antihypertensives aimed at attenuating the morning blood pressure surge. In the elderly, this response may be paradoxical or even absent, due to the deterioration of blood pressure rhythms and age-related autonomic dysfunction (Albuquerque et al., 2021; Akyel et al., 2023). Oncology protocols based on chronotolerance and chronoefficacy often result in poorer outcomes in older adults, with high interindividual variability and an increased risk of hematologic, neurologic, and gastrointestinal toxicity (Ballesta et al., 2017; Puppala et al., 2021; Amiama-Roig et al., 2022).

This loss of chronobiological responsiveness stems both from the desynchronization between central and peripheral clocks, and from the chronotype heterogeneity observed with aging, as the geriatric tendency toward morning chronotypes may not align with dosing regimens designed for younger adults with evening chronotypes (Dallmann et al., 2014; Kara et al., 2023). Added to this is the pharmacodynamic remodeling associated with aging, with changes in receptors, ion channels, and signaling pathways linked to the circadian system (Ozturk et al., 2017; Zhao et al., 2020).

In light of this scenario, personalized medicine emerges as a promising strategy to counteract the deleterious effects of aging on biological rhythmicity and to enhance therapeutic responses in elderly patients. Devices capable of accurately estimating an individual’s circadian phase have become increasingly valuable tools for aligning interventions not only with the disease but also with the residual rhythmicity of older individuals (Dose et al., 2023). Among these technologies, TimeTeller utilizes transcriptomic data from a single biological sample to analyze the coordinated expression of a multigenic panel composed of core elements of the molecular clock, enabling the inference of internal biological time with high precision. This approach is highly innovative and overcomes the limitations of traditional methods, which rely on indirect markers or behavioral measurements (Dose et al., 2023; Vlachou et al., 2024).

In this regard, as the circadian system is plastic and modifiable, chronomodulation, the strategic alignment of behaviors, treatments, and molecular interventions with the body’s internal clock, offers a compelling and multifaceted strategy to counteract the circadian system decay. Beyond lifestyle-based approaches such as timed light exposure, structured sleep, and feeding-fasting cycles that enhance circadian amplitude and metabolic resilience, molecular chronotherapeutics are gaining traction. Central to this molecular axis are chrono-metabolites, endogenous compounds like NAD^+^, polyamines, and urolithins, whose rhythmic oscillations bridge circadian regulation with metabolic integrity. With aging, the decline in these oscillations compromises core clock function, disrupts mitochondrial homeostasis, and weakens stress responses. Targeted restoration of their rhythmicity, via supplements such as nicotinamide riboside, dietary polyamines, or urolithin A, has been shown to rejuvenate circadian gene expression, synchronize metabolic rhythms, and improve physical performance in aging models. Together, these interventions illustrate how chronomodulation, from behavior to metabolite, stands not only as a preventive strategy but as a foundational pillar of precision geroscience, where aging is rhythmically recalibrated to preserve physiological harmony (Sato et al., 2017; Mukherji et al., 2019; Dong et al., 2020; Verma et al., 2023; Xu et al., 2023). Moreover, circadian-aligned interventions, such as timed light exposure, structured physical activity, meal scheduling, and sleep optimization, hold promise for enhancing drug tolerability, improving therapeutic outcomes, and promoting healthspan in the elderly.

Moreover, understanding whether the target molecule for treating a specific disease is subject to circadian regulation could aid in optimizing the timing of drug administration. In this context, ALZ-801, which targets APOEε4 in Alzheimer’s disease, is currently being evaluated in two clinical trials (NCT04693520, NCT06304883) (Kroemer et al., 2025). Notably, APOE^−/−^ mice exhibit altered rhythms of peripheral and central clock genes in heart and liver tissues, characterized by increased amplitudes and phase shifts, effects that are further exacerbated by a high-fat diet (Xu et al., 2009). Additionally, disruption of the core clock gene *Bmal1* in the brain leads to elevated *Apoe* expression and accelerated amyloid plaque accumulation (Hussain et al., 2024). Another example is Resmetirom, a thyroid hormone receptor-β agonist used to treat non-alcoholic fatty liver disease, a condition highly prevalent in aging, which reduces the expression of DBI, a gene classified as a gero-gene (Kroemer et al., 2025). DBI exhibits circadian oscillation in both the suprachiasmatic nucleus and the liver (Montégut et al., 2023). Thus, it would be of particular interest to further investigate the chronotherapeutic potential of ALZ-801 or Resmetirom to determine whether its efficacy or tolerability is influenced by the timing of administration, in alignment with the circadian nature of their targets.

In parallel, chronotimed drug delivery technologies and stimulus-responsive nanoparticles (e.g., to pH and temperature) represent other technological advances that have also shown the capacity to align drug administration with the patient’s biological phase. These tools contrast with strategies that aim to reprogram hepatic rhythmicity, such as compounds that act on the molecular clock (e.g., *RORα* agonists and *REV-ERB* inhibitors).

Currently, several chronomodulated therapies have proven their efficacy. In this regard, in cardiovascular diseases, it has been evaluated that taking a low dose of acetylsalicylic acid (ASA) in the evening, rather than the morning, is more effective since it aligns better with the body’s circadian rhythm, potentially reducing the risk of cardiovascular events. The limited 24-h efficacy of once-daily ASA is partly explained by its pharmacokinetics and the circadian physiology of platelets. ASA is rapidly absorbed and eliminated, so newly produced platelets, released at a rate of 10%–15% per day, escape COX-1 inhibition and can form clots. Effective platelet inhibition requires ∼95% COX-1 blockade, yet studies show that 24 h after morning intake, a quarter of cardiovascular patients have insufficient inhibition. Notably, platelet production follows a circadian rhythm, peaking in the late night and early morning, coinciding with the timing of adverse cardiovascular events (Buurma et al., 2019).

In epilepsy, administering a higher evening dose of clobazam has been shown to improve seizure control in patients whose seizures occur predominantly at night or in the early morning. Tailoring drug administration to individual seizure susceptibility patterns exemplifies how chronotherapy can optimize epilepsy management: differential dosing allows for delivering higher treatment levels at the times of greatest vulnerability, while minimizing side effects during periods of lower risk (Thome-Souza et al., 2016).

Chronomodulated treatment also shows promise in metabolic diseases, particularly via time-restricted eating and timed administration of antihypertensives or metformin (Hermida et al., 2011; Türk et al., 2023). Several clinical trials and modeling studies support the concept that aligning treatment with circadian rhythms can enhance efficacy and reduce risks.

## 8 Conclusion

The convergence of circadian biology and aging research reveals time not merely as a backdrop but as a dynamic biological force, where *Kronos*, that represents the linear progression of age, and *Kairos*, the opportune moment dictated by biological rhythms, co-determine the trajectory of health and disease.

The circadian system emerges as a central integrator of internal physiology and external temporal cues, shaping molecular pathways implicated in longevity, age-related diseases, and the systemic resilience observed in long-lived species. Disruptions to this temporal architecture, whether through genetic mutations, lifestyle factors, or social disconnection, can accelerate the hallmarks of aging and compromise physiological homeostasis, as seen in natural aging. It would be worthwhile to understand how the circadian system operates in contexts of accelerated aging, such as in progeroid syndromes, both in preclinical models and in humans, to uncover potential shared mechanisms.

This lesson is being learned from long-lived species such as the naked mole-rat, cavefish, and certain whales, which appear to age at a slower pace than other animals. The studies suggest that genome stability, modulation of the mTOR pathway, glucose metabolism, and circadian plasticity act in concert in these species to delay aging and extend lifespan. A key factor underlying this phenomenon is the stable environment in which these species live, coupled with their remarkable ability to adapt swiftly and with high plasticity to external changes when necessary. Conceptually, as with the stem cell pool, the less you exhaust it, the more reserve you retain later in life. In this light, living in a stable environment may reduce the need for constant circadian adjustments, thereby preserving the integrity of the circadian system over time.

The concept of the chrono-exposome, introduced in this review, broadens this perspective by situating circadian rhythms within the wider context of temporal environmental and behavioral exposures throughout life. From this viewpoint, lifelong exposure to various stressors can disrupt circadian homeostasis and accelerate aging. Such disruptions can begin early, for example, when neonates in intensive care units are exposed to inappropriate light cycles, continue through infancy and adolescence with irregular exposure to screens and erratic eating patterns, persist into adulthood where jetlag and shift work are common, and extend into older age with increased sedentarism and psychosocial isolation.

In this context, the interplay between circadian rhythms and pharmacological responses gains critical relevance in the aging population. As aging weakens both the amplitude and synchronization of circadian oscillations, the efficacy and safety of pharmacological interventions are increasingly compromised. This decline in circadian robustness affects drug metabolism, detoxification, and target engagement, while also contributing to the heterogeneity in therapeutic outcomes among older adults. Chronotherapeutic approaches, though promising, must account for the altered rhythmic landscape of the elderly, whose chronotypes, molecular clocks, and peripheral rhythms are no longer aligned with standard dosing paradigms. Innovations such as transcriptome-based circadian phase estimators, rhythmic biomarker profiling, and chrono-active compounds open new avenues for aligning treatments with residual circadian function. As such, circadian-informed precision geromedicine, where both the timing and the nature of interventions are tailored to the aging clock, emerges as a foundational strategy not only to enhance therapeutic efficacy and reduce toxicity, but also to recalibrate biological time itself, preserving physiological harmony across the aging trajectory.

As we advance toward precision geromedicine, recognizing the plasticity of the circadian system to recalibrate homeostasis, restoring circadian integrity through personalized, time-aligned interventions emerges as a promising strategy to mitigate age-related decline and improve therapeutic outcomes. Embracing time not merely as a measurable factor but as a modifiable variable may ultimately enable us to harmonize human aging with biological opportunity.

Future circadian aging research should integrate molecular, environmental, and therapeutic approaches, deciphering shared longevity pathways with those that keep a healthy circadian system, mitigating chrono-exposome insults, and tailoring chronotherapy to the changing clock across life. Protecting genome stability, metabolic balance, and circadian plasticity emerges as essential for the health of both the organism and its circadian system. Ultimately, preserving temporal harmony may prove as vital as preserving life itself.

## References

[B1] Acosta-Rodríguez V. A. Rijo-Ferreira F. Green C. B. Takahashi J. S. (2021). Importance of circadian timing for aging and longevity. Nat. Commun. 12 (1), 2862. 10.1038/s41467-021-22922-6 34001884 PMC8129076

[B2] Acosta-Rodríguez V. Rijo-Ferreira F. Izumo M. Xu P. Wight-Carter M. Green C. B. (2022). Circadian alignment of early onset caloric restriction promotes longevity in male C57BL/6J mice. Science 376 (6598), 1192–1202. 10.1126/science.abk0297 35511946 PMC9262309

[B3] Acosta-Rodríguez V. A. Rijo-Ferreira F. van Rosmalen L. Izumo M. Park N. Joseph C. (2024). Misaligned feeding uncouples daily rhythms within brown adipose tissue and between peripheral clocks. Cell Rep. 43 (8), 114523. 10.1016/j.celrep.2024.114523 39046875 PMC12223410

[B4] Ahmed R. Reza H. M. Shinohara K. Nakahata Y. (2022). Cellular senescence and its impact on the circadian clock. J. Biochem. 171 (5), 493–500. 10.1093/jb/mvab115 34668549

[B5] Akyel Y. K. Ozturk Civelek D. Ozturk Seyhan N. Gul S. Gazioglu I. Pala Kara Z. (2023). Diurnal changes in capecitabine clock-controlled metabolism enzymes are responsible for its pharmacokinetics in Male mice. J. Biol. Rhythms 38 (2), 171–184. 10.1177/07487304221148779 36762608 PMC10037547

[B6] Albrecht U. (2017). The circadian clock, metabolism and obesity. Obes. Rev. 18 (S1), 25–33. 10.1111/obr.12502 28164453

[B7] Albuquerque T. Neves A. R. Quintela T. Costa D. (2021). Exploring the link between chronobiology and drug delivery: effects on cancer therapy. J. Mol. Med. 99 (10), 1349–1371. 10.1007/s00109-021-02106-x 34213595

[B8] Alibhai F. J. Tsimakouridze E. V. Chinnappareddy N. Wright D. C. Billia F. O'Sullivan M. L. (2014). Short-Term disruption of diurnal rhythms after Murine myocardial infarction adversely affects long-term myocardial structure and function. Circulation Res. 114 (11), 1713–1722. 10.1161/CIRCRESAHA.114.302995 24687134

[B9] Allada R. Bass J. (2021). Circadian mechanisms in medicine. N. Engl. J. Med. 384 (6), 550–561. 10.1056/NEJMra1802337 33567194 PMC8108270

[B10] Allison K. C. Hopkins C. M. Ruggieri M. Spaeth A. M. Ahima R. S. Zhang Z. (2021). Prolonged, controlled daytime *versus* delayed eating impacts weight and metabolism. Curr. Biol. 31 (3), 650–657.e3. 10.1016/j.cub.2020.10.092 33259790 PMC7878354

[B11] Altamirano F. G. Castro-Pascual I. Ponce I. T. Coria-Lucero C. D. Cargnelutti E. Ferramola M. L. (2024). Late-Onset caloric restriction improves cognitive performance and restores circadian patterns of neurotrophic, clock, and epigenetic factors in the hippocampus of old Male rats. J. Gerontol. A Biol. Sci. Med. Sci. 80 (1), glae252. 10.1093/gerona/glae252 39447038

[B12] Altman N. G. Izci-Balserak B. Schopfer E. Jackson N. Rattanaumpawan P. Gehrman P. R. (2012). Sleep duration *versus* sleep insufficiency as predictors of cardiometabolic health outcomes. Sleep. Med. 13 (10), 1261–1270. 10.1016/j.sleep.2012.08.005 23141932 PMC3527631

[B13] Amiama-Roig A. Verdugo-Sivianes E. M. Carnero A. Blanco J. R. (2022). Chronotherapy: circadian rhythms and their influence in cancer therapy. Cancers 14 (20), 5071. 10.3390/cancers14205071 36291855 PMC9599830

[B14] Anea C. B. Zhang M. Stepp D. W. Simkins G. B. Reed G. Fulton D. J. (2009). Vascular disease in mice with a dysfunctional circadian clock. Circulation 119 (11), 1510–1517. 10.1161/CIRCULATIONAHA.108.827477 19273720 PMC2761686

[B15] Anisimov N. Vinogradova I. A. Panchenko A. V. Popovich I. G. Zabezhinski M. A. (2013). Light-at-Night-Induced circadian disruption, cancer and aging. Curr. Aging Sci. 5 (3), 170–177. 10.2174/1874609811205030002 23237593

[B16] Archer S. N. Möller-Levet C. Bonmatí-Carrión M. Á. Laing E. E. Dijk D. J. (2024). Extensive dynamic changes in the human transcriptome and its circadian organization during prolonged bed rest. iScience 27 (3), 109331. 10.1016/j.isci.2024.109331 38487016 PMC10937834

[B17] Arregi A. Vegas O. Lertxundi A. Silva A. Ferreira I. Bereziartua A. (2024). Road traffic noise exposure and its impact on health: evidence from animal and human studies—chronic stress, inflammation, and oxidative stress as key components of the complex downstream pathway underlying noise-induced non-auditory health effects. Environ. Sci. Pollut. Res. 31 (34), 46820–46839. 10.1007/s11356-024-33973-9 38977550 PMC11297122

[B18] Asher G. Sassone-Corsi P. (2015). Time for food: the intimate interplay between nutrition, metabolism, and the circadian clock. Cell 161 (1), 84–92. 10.1016/j.cell.2015.03.015 25815987

[B19] Asher G. Gatfield D. Stratmann M. Reinke H. Dibner C. Kreppel F. (2008). SIRT1 regulates circadian clock gene expression through PER2 deacetylation. Cell 134 (2), 317–328. 10.1016/j.cell.2008.06.050 18662546

[B20] Ashimori A. Nakahata Y. Sato T. Fukamizu Y. Matsui T. Yoshitane H. (2021). Attenuated SIRT1 activity leads to PER2 cytoplasmic localization and Dampens the amplitude of Bmal1 promoter-driven circadian oscillation. Front. Neurosci. 15, 647589. 10.3389/fnins.2021.647589 34108855 PMC8180908

[B21] Astone M. Oberkersch R. E. Tosi G. Biscontin A. Santoro M. M. (2023). The circadian protein BMAL1 supports endothelial cell cycle during angiogenesis. Cardiovasc. Res. 119 (10), 1952–1968. 10.1093/cvr/cvad057 37052172

[B22] Ayyar V. S. Sukumaran S. (2021). Circadian rhythms: influence on physiology, pharmacology, and therapeutic interventions. J. Pharmacokinet. Pharmacodynamics 48 (3), 321–338. 10.1007/s10928-021-09751-2 33797011 PMC8015932

[B23] Bainier C. Mateo M. Felder-Schmittbuhl M. P. Mendoza J. (2017). Circadian rhythms of hedonic drinking behavior in mice. Neuroscience 349, 229–238. 10.1016/j.neuroscience.2017.03.002 28286126

[B24] Ballesta A. Innominato P. F. Dallmann R. Rand D. A. Lévi F. A. (2017). Systems chronotherapeutics. Pharmacol. Rev. 69 (2), 161–199. 10.1124/pr.116.013441 28351863 PMC5394920

[B25] Baraldo M. Furlanut M. (2006). Chronopharmacokinetics of ciclosporin and tacrolimus. Clin. Pharmacokinet. 45 (8), 775–788. 10.2165/00003088-200645080-00002 16884317

[B26] Baron K. G. Reid K. J. Wolfe L. F. Attarian H. Zee P. C. (2018). Phase relationship between DLMO and sleep onset and the risk of metabolic disease among normal weight and Overweight/Obese adults. J. Biol. Rhythms 33 (1), 76–83. 10.1177/0748730417745914 29262758 PMC7201427

[B27] Barone I. Gilette N. M. Hawks-Mayer H. Handy J. Zhang K. J. Chifamba F. F. (2023). Synaptic BMAL1 phosphorylation controls circadian hippocampal plasticity. Sci. Adv. 9 (43), eadj1010. 10.1126/sciadv.adj1010 37878694 PMC10599629

[B28] Barroggi Constantino D. Lederle K. A. Middleton B. Revell V. L. Sletten T. L. Williams P. (2025). The bright and dark side of blue-enriched light on sleep and activity in older adults. GeroScience 47, 3927–3939. 10.1007/s11357-025-01506-y 39821044 PMC12181448

[B29] Bartsch C. (2010). Light-at-night, cancer and aging. Aging 2 (2), 76–77. 10.18632/aging.100126 20354267 PMC2850142

[B30] Basisty N. Kale A. Jeon O. H. Kuehnemann C. Payne T. Rao C. (2020). A proteomic atlas of senescence-associated secretomes for aging biomarker development. PLOS Biol. 18 (1), e3000599. 10.1371/journal.pbio.3000599 31945054 PMC6964821

[B31] Beale A. Guibal C. Tamai T. K. Klotz L. Cowen S. Peyric E. (2013). Circadian rhythms in Mexican blind cavefish Astyanax mexicanus in the lab and in the field. Nat. Commun. 4 (1), 2769. 10.1038/ncomms3769 24225650

[B32] Beker M. C. Caglayan B. Caglayan A. B. Kelestemur T. Yalcin E. Caglayan A. (2019). Interaction of melatonin and Bmal1 in the regulation of PI3K/AKT pathway components and cellular survival. Sci. Rep. 9 (1), 19082. 10.1038/s41598-019-55663-0 31836786 PMC6910929

[B33] Belancio V. P. Blask D. E. Deininger P. Hill S. M. Jazwinski S. M. (2015). The aging clock and circadian control of metabolism and genome stability. Front. Genet. 5, 455. 10.3389/fgene.2014.00455 25642238 PMC4294216

[B34] Bellet M. M. Sassone-Corsi P. (2010). Mammalian circadian clock and metabolism – the epigenetic link. J. Cell Sci. 123 (22), 3837–3848. 10.1242/jcs.051649 21048160 PMC2972271

[B35] Bennett N. C. Faulkes C. G. (2000). African mole-rats: ecology and eusociality. Cambridge: Cambridge University Press.

[B36] Berger S. E. Ordway M. R. Schoneveld E. Lucchini M. Thakur S. Anders T. (2023). The impact of extreme summer temperatures in the United Kingdom on infant sleep: implications for learning and development. Sci. Rep. 13 (1), 10061. 10.1038/s41598-023-37111-2 37344536 PMC10284886

[B37] Bilandžija H. Ćetković H. Jeffery W. R. (2012). Evolution of albinism in cave planthoppers by a convergent defect in the first step of melanin biosynthesis. Evol. and Dev. 14 (2), 196–203. 10.1111/j.1525-142X.2012.00535.x 23017027 PMC6169799

[B38] Bilandžija H. Hollifield B. Steck M. Meng G. Ng M. Koch A. D. (2020). Phenotypic plasticity as a mechanism of cave colonization and adaptation. eLife 9, e51830. 10.7554/eLife.51830 32314737 PMC7173965

[B39] Biswas J. (2010). Kotumsar Cave biodiversity: a review of cavernicoles and their troglobiotic traits. Biodivers. Conservation 19 (1), 275–289. 10.1007/s10531-009-9710-7

[B40] Blacher E. Tsai C. Litichevskiy L. Shipony Z. Iweka C. A. Schneider K. M. (2022). Aging disrupts circadian gene regulation and function in macrophages. Nat. Immunol. 23 (2), 229–236. 10.1038/s41590-021-01083-0 34949832 PMC9704320

[B41] Blin M. Fumey J. Lejeune C. Policarpo M. Leclercq J. Père S. (2020). Diversity of olfactory responses and skills in Astyanax Mexicanus cavefish populations inhabiting different Caves. Diversity 12 (10), 395. 10.3390/d12100395

[B42] Bolsius Y. G. Zurbriggen M. D. Kim J. K. Kas M. J. Meerlo P. Aton S. J. (2021). The role of clock genes in sleep, stress and memory. Biochem. Pharmacol. 191, 114493. 10.1016/j.bcp.2021.114493 33647263 PMC9487905

[B43] Bonmati-Carrion M.-A. Tomas-Loba A. (2021). Melatonin and cancer: a polyhedral network where the source matters. Antioxidants 10 (2), 210. 10.3390/antiox10020210 33535472 PMC7912767

[B44] Brunet A. Forsberg F. Fan Q. Sæther T. Collas P. (2019). Nuclear lamin B1 interactions with chromatin during the circadian cycle are uncoupled from periodic gene expression. Front. Genet. 10, 917. 10.3389/fgene.2019.00917 31632442 PMC6785633

[B45] Buffenstein R. (2005). The naked Mole-Rat: a new long-living model for human aging research. Journals Gerontology Ser. A Biol. Sci. Med. Sci. 60 (11), 1369–1377. 10.1093/gerona/60.11.1369 16339321

[B46] Buffenstein R. (2008). Negligible senescence in the longest living rodent, the naked mole-rat: insights from a successfully aging species. J. Comp. Physiology B 178 (4), 439–445. 10.1007/s00360-007-0237-5 18180931

[B47] Buijink M. R. Michel S. (2021). A multi‐level assessment of the bidirectional relationship between aging and the circadian clock. J. Neurochem. 157 (1), 73–94. 10.1111/jnc.15286 33370457 PMC8048448

[B48] Buijink M. R. van Weeghel M. Harms A. Murli D. S. Meijer J. H. Hankemeier T. (2024). Loss of temporal coherence in the circadian metabolome across multiple tissues during ageing in mice. Eur. J. Neurosci. 60 (2), 3843–3857. 10.1111/ejn.16428 38802069

[B49] Buurma M. van Diemen J. J. K. Thijs A. Numans M. E. Bonten T. N. (2019). Circadian rhythm of cardiovascular disease: the potential of chronotherapy with aspirin. Front. Cardiovasc. Med. 6, 84. 10.3389/fcvm.2019.00084 31281821 PMC6595227

[B50] Cabanillas R. Cadiñanos J. Villameytide J. A. F. Pérez M. Longo J. Richard J. M. (2011). Néstor–Guillermo progeria syndrome: a novel premature aging condition with early onset and chronic development caused by *BANF1* mutations. Am. J. Med. Genet. Part A 155 (11), 2617–2625. 10.1002/ajmg.a.34249 21932319

[B51] Cai R. Gao L. Gao C. Yu L. Zheng X. Bennett D. (2023). Circadian disturbances and frailty risk in older adults: a prospective cohort study. Res. Sq. 10.21203/rs.3.rs-2648399/v1 37973796 PMC10654720

[B52] Cao R. (2018). mTOR signaling, translational control, and the circadian clock. Front. Genet. 9, 367. 10.3389/fgene.2018.00367 30250482 PMC6139299

[B53] Cao Y. Wang R.-H. (2017). Associations among metabolism, circadian rhythm and age-associated diseases. Aging Dis. 8 (3), 314–333. 10.14336/AD.2016.1101 28580187 PMC5440111

[B54] Caravia X. M. Roiz-Valle D. Morán-Álvarez A. López-Otín C. (2017). Functional relevance of miRNAs in premature ageing. Mech. Ageing Dev. 168, 10–19. 10.1016/j.mad.2017.05.003 28502819

[B55] Caravia X. M. Fanjul V. Oliver E. Roiz-Valle D. Morán-Álvarez A. Desdín-Micó G. (2018). The microRNA-29/PGC1α regulatory axis is critical for metabolic control of cardiac function. PLOS Biol. 16 (10), e2006247. 10.1371/journal.pbio.2006247 30346946 PMC6211751

[B56] Caravia X. M. Ramirez-Martinez A. Gan P. Wang F. McAnally J. R. Xu L. (2022). Loss of function of the nuclear envelope protein LEMD2 causes DNA damage–dependent cardiomyopathy. J. Clin. Investigation 132 (22), e158897. 10.1172/JCI158897 36377660 PMC9663152

[B57] Cardinali D. P. Hardeland R. (2017). Inflammaging, metabolic syndrome and melatonin: a call for treatment studies. Neuroendocrinology 104 (4), 382–397. 10.1159/000446543 27165273

[B58] Carlson B. M. Gross J. B. (2018). Characterization and comparison of activity profiles exhibited by the cave and surface morphotypes of the blind Mexican tetra, Astyanax mexicanus. Comp. Biochem. Physiology Part C Toxicol. and Pharmacol. 208, 114–129. 10.1016/j.cbpc.2017.08.002 28823830 PMC5817046

[B59] Carrier J. Monk T. H. Buysse D. J. Kupfer D. J. (1997). Sleep and morningness-eveningness in the “middle” years of life (20-59 y). J. Sleep Res. 6 (4), 230–237. 10.1111/j.1365-2869.1997.00230.x 9493522

[B60] Carrier J. Land S. Buysse D. J. Kupfer D. J. Monk T. H. (2001). The effects of age and gender on sleep EEG power spectral density in the middle years of life (ages 20-60 years old). Psychophysiology 38 (2), 232–242. 10.1017/s0048577201991838 11347869

[B61] Carrier J. Paquet J. Morettini J. Touchette E. (2002). Phase advance of sleep and temperature circadian rhythms in the middle years of life in humans. Neurosci. Lett. 320 (1–2), 1–4. 10.1016/s0304-3940(02)00038-1 11849749

[B62] Carrier J. Frenette S. Montplaisir J. Paquet J. Drapeau C. Morettini J. (2005). Effects of periodic leg movements during sleep in middle-aged subjects without sleep complaints. Mov. Disord. 20 (9), 1127–1132. 10.1002/mds.20506 15884036

[B63] Carter B. Justin H. S. Gulick D. Gamsby J. J. (2021). The molecular clock and neurodegenerative disease: a stressful time. Front. Mol. Biosci. 8, 644747. 10.3389/fmolb.2021.644747 33889597 PMC8056266

[B64] Chan K. Wong F. S. Pearson J. A. (2022). Circadian rhythms and pancreas physiology: a review. Front. Endocrinol. 13, 920261. 10.3389/fendo.2022.920261 36034454 PMC9399605

[B65] Chang H.-C. Guarente L. (2013). SIRT1 mediates central circadian control in the SCN by a mechanism that decays with aging. Cell 153 (7), 1448–1460. 10.1016/j.cell.2013.05.027 23791176 PMC3748806

[B66] Chellappa K. Brinkman J. A. Mukherjee S. Morrison M. Alotaibi M. I. Carbajal K. A. (2019). Hypothalamic mTORC2 is essential for metabolic health and longevity. Aging Cell 18 (5), e13014. 10.1111/acel.13014 31373126 PMC6718533

[B67] Chen Y. Wang D. Song Y. Zhang X. Jiao Z. Dong J. (2019). Functional polymorphisms in circadian positive feedback loop genes predict postsurgical prognosis of gastric cancer. Cancer Med. 8 (4), 1919–1929. 10.1002/cam4.2050 30843665 PMC6488121

[B68] Cheng T. S. Loy S. L. Toh J. Y. Cheung Y. B. Chan J. K. Y. Godfrey K. M. (2016). Predominantly nighttime feeding and weight outcomes in infants. Am. J. Clin. Nutr. 104 (2), 380–388. 10.3945/ajcn.116.130765 27385614

[B69] Cheng W.-Y. Chan P. L. Ong H. Y. Wong K. H. Chang R. C. C. (2024). Systemic inflammation disrupts circadian rhythms and diurnal neuroimmune dynamics. Int. J. Mol. Sci. 25 (13), 7458. 10.3390/ijms25137458 39000563 PMC11242289

[B70] Chhunchha B. Kubo E. Singh D. P. (2020). Clock protein Bmal1 and Nrf2 cooperatively control aging or oxidative response and redox homeostasis by regulating rhythmic expression of Prdx6. Cells 9 (8), 1861. 10.3390/cells9081861 32784474 PMC7463585

[B71] Chiang P.-L. Hao W. R. Hong H. J. Chen C. C. Chiu C. C. Fang Y. A. (2023). The effects of different types of sleep disorder on colorectal cancer: a nationwide population-based cohort Study. Cancers 15 (19), 4728. 10.3390/cancers15194728 37835421 PMC10571828

[B72] Christian C. J. Benian G. M. (2020). Animal models of sarcopenia. Aging Cell 19 (10), e13223. 10.1111/acel.13223 32857472 PMC7576270

[B73] Ciarleglio C. M. Resuehr H. E. S. McMahon D. G. (2011). Interactions of the serotonin and circadian systems: nature and nurture in rhythms and blues. Neuroscience 197, 8–16. 10.1016/j.neuroscience.2011.09.036 21963350

[B74] Collis S. J. Boulton S. J. (2007). Emerging links between the biological clock and the DNA damage response. Chromosoma 116 (4), 331–339. 10.1007/s00412-007-0108-6 17492458

[B75] Corella D. Asensio E. M. Coltell O. Sorlí J. V. Estruch R. Martínez-González M. Á. (2016). CLOCK gene variation is associated with incidence of type-2 diabetes and cardiovascular diseases in type-2 diabetic subjects: dietary modulation in the PREDIMED randomized trial. Cardiovasc. Diabetol. 15 (1), 4. 10.1186/s12933-015-0327-8 26739996 PMC4704407

[B76] Costello H. M. Sharma R. K. McKee A. R. Gumz M. L. (2023). Circadian disruption and the molecular clock in atherosclerosis and hypertension. Can. J. Cardiol. 39 (12), 1757–1771. 10.1016/j.cjca.2023.06.416 37355229 PMC11446228

[B77] Covassin N. Singh P. Somers V. K. (2016). Keeping up with the clock: circadian disruption and obesity risk. Hypertension 68 (5), 1081–1090. 10.1161/HYPERTENSIONAHA.116.06588 27620394 PMC5063707

[B78] Cox K. H. Takahashi J. S. (2019). Circadian clock genes and the transcriptional architecture of the clock mechanism. J. Mol. Endocrinol. 63 (4), R93–R102. 10.1530/JME-19-0153 31557726 PMC6872945

[B79] Crespo M. T. Trebucq L. L. Senna C. A. Hokama G. Paladino N. Agostino P. V. (2025). Circadian disruption of feeding-fasting rhythm and its consequences for metabolic, immune, cancer, and cognitive processes. Biomed. J. 48, 100827. 10.1016/j.bj.2025.100827 39756653 PMC12164040

[B80] Crish S. D. Dengler‐Crish C. M. Catania K. C. (2006). Central visual system of the naked mole‐rat (*Heterocephalus glaber*). Anatomical Rec. Part A Discov. Mol. Cell. Evol. Biol. 288A (2), 205–212. 10.1002/ar.a.20288 16419086

[B81] Crnko S. Du Pré B. C. Sluijter J. P. G. Van Laake L. W. (2019). Circadian rhythms and the molecular clock in cardiovascular biology and disease. Nat. Rev. Cardiol. 16 (7), 437–447. 10.1038/s41569-019-0167-4 30796369

[B82] Cruz-Jentoft A. J. Sayer A. A. (2019). Sarcopenia. Lancet 393 (10191), 2636–2646. 10.1016/S0140-6736(19)31138-9 31171417

[B83] Culver D. C. (2014). Shallow subterranean habitats: ecology, evolution, and conservation. Oxford: Oxford University Press.

[B84] Culver D. C. Pipan T. (2019). The biology of caves and other subterranean habitats. Second edition. Oxford: Oxford University Press.

[B85] Dahlberg L. von Saenger I. Naseer M. Lennartsson C. Agahi N. (2024). National trends in loneliness and social isolation in older adults: an examination of subgroup trends over three decades in Sweden. Front. Public Health 12, 1444990. 10.3389/fpubh.2024.1444990 39324154 PMC11422125

[B86] Daiber A. Frenis K. Kuntic M. Li H. Wolf E. Kilgallen A. B. (2022). Redox regulatory changes of circadian rhythm by the environmental risk factors traffic noise and air pollution. Antioxidants and Redox Signal. 37 (10–12), 679–703. 10.1089/ars.2021.0272 35088601 PMC9618394

[B87] Dallmann R. Viola A. U. Tarokh L. Cajochen C. Brown S. A. (2012). The human circadian metabolome. Proc. Natl. Acad. Sci. U. S. A. 109 (7), 2625–2629. 10.1073/pnas.1114410109 22308371 PMC3289302

[B88] Dallmann R. Brown S. A. Gachon F. (2014). Chronopharmacology: new insights and therapeutic implications. Annu. Rev. Pharmacol. Toxicol. 54 (1), 339–361. 10.1146/annurev-pharmtox-011613-135923 24160700 PMC3885389

[B89] Dallmann R. Okyar A. Lévi F. (2016). Dosing-Time makes the poison: circadian regulation and pharmacotherapy. Trends Mol. Med. 22 (5), 430–445. 10.1016/j.molmed.2016.03.004 27066876

[B90] Das J. K. Banskota N. Candia J. Griswold M. E. Orenduff M. de Cabo R. (2023). Calorie restriction modulates the transcription of genes related to stress response and longevity in human muscle: the CALERIE study. Aging Cell 22 (12), e13963. 10.1111/acel.13963 37823711 PMC10726900

[B91] De Assis L. V. M. Kinker G. S. Moraes M. N. Markus R. P. Fernandes P. A. Castrucci A. M. d. L. (2018). Expression of the circadian clock gene BMAL1 positively correlates with antitumor immunity and patient survival in metastatic Melanoma. Front. Oncol. 8, 185. 10.3389/fonc.2018.00185 29946530 PMC6005821

[B92] De La Maza-Bustindui N. S. León-Álvarez M. Ponce-Acosta C. Zarco-Morales K. P. Fermín-Martínez C. A. Antonio-Villa N. E. (2025). Impact of cardiometabolic risk factors and its management on the reversion and progression of arterial stiffness. npj Cardiovasc. Health 2 (1), 36. 10.1038/s44325-025-00074-6 PMC12912415 41776052

[B93] De Souza P. E. Souza-Silva M. Ferreira R. L. (2024). The ticking clock in the dark: review of biological rhythms in cave invertebrates. Chronobiology Int. 41 (5), 738–756. 10.1080/07420528.2024.2348010 38722073

[B94] Dijk D. J. Duffy J. F. (1999). Circadian regulation of human sleep and age-related changes in its timing, consolidation and EEG characteristics. Ann. Med. 31 (2), 130–140. 10.3109/07853899908998789 10344586

[B95] Dominick G. Berryman D. E. List E. O. Kopchick J. J. Li X. Miller R. A. (2015). Regulation of mTOR activity in snell dwarf and GH receptor gene-disrupted mice. Endocrinology 156 (2), 565–575. 10.1210/en.2014-1690 25456069 PMC4298324

[B96] Donato A. J. Machin D. R. Lesniewski L. A. (2018). Mechanisms of dysfunction in the aging vasculature and role in age-related disease. Circulation Res. 123 (7), 825–848. 10.1161/CIRCRESAHA.118.312563 30355078 PMC6207260

[B97] Dong D. Yang D. Lin L. Wang S. Wu B. (2020). Circadian rhythm in pharmacokinetics and its relevance to chronotherapy. Biochem. Pharmacol. 178, 114045. 10.1016/j.bcp.2020.114045 32446886

[B98] Dose B. Yalçin M. Dries S. P. M. Relógio A. (2023). TimeTeller for timing health: the potential of circadian medicine to improve performance, prevent disease and optimize treatment. Front. Digital Health 5, 1157654. 10.3389/fdgth.2023.1157654 37153516 PMC10155816

[B99] Duffy J. F. Zitting K.-M. Chinoy E. D. (2015). Aging and circadian rhythms. Sleep. Med. Clin. 10 (4), 423–434. 10.1016/j.jsmc.2015.08.002 26568120 PMC4648699

[B100] Edrey Y. H. Hanes M. Pinto M. Mele J. Buffenstein R. (2011). Successful aging and sustained good health in the naked mole rat: a long-lived Mammalian model for biogerontology and biomedical research. ILAR J. 52 (1), 41–53. 10.1093/ilar.52.1.41 21411857

[B101] Eide E. J. Woolf M. F. Kang H. Woolf P. Hurst W. Camacho F. (2005). Control of mammalian circadian rhythm by CKIepsilon-regulated proteasome-mediated PER2 degradation. Mol. Cell. Biol. 25 (7), 2795–2807. 10.1128/MCB.25.7.2795-2807.2005 15767683 PMC1061645

[B102] Elahi D. Muller D. C. Egan J. M. Andres R. Veldhuis J. D. Meneilly G. S. (2002). “Glucose tolerance, glucose utilization and insulin secretion in ageing,” in Novartis foundation symposia. Editors Chadwick D. J. Goode J. A. 1st ed. (Wiley), 222–246. 10.1002/0470846542.ch14 11855690

[B103] Eldesoky E. S. (2007). Pharmacokinetic-Pharmacodynamic crisis in the elderly. Am. J. Ther. 14 (5), 488–498. 10.1097/01.mjt.0000183719.84390.4d 17890940

[B104] Eriksson M. Brown W. T. Gordon L. B. Glynn M. W. Singer J. Scott L. (2003). Recurrent *de novo* point mutations in lamin A cause Hutchinson–Gilford progeria syndrome. Nature 423 (6937), 293–298. 10.1038/nature01629 12714972 PMC10540076

[B105] Ezpeleta M. Cienfuegos S. Lin S. Pavlou V. Gabel K. Tussing-Humphreys L. (2024). Time-restricted eating: watching the clock to treat obesity. Cell Metab. 36 (2), 301–314. 10.1016/j.cmet.2023.12.004 38176412 PMC11221496

[B106] Ezzati A. Tamargo J. A. Golberg L. Haub M. D. Anton S. D. (2025). The effects of time-restricted eating on inflammation and oxidative stress in overweight older adults: a pilot Study. Nutrients 17 (2), 322. 10.3390/nu17020322 39861451 PMC11768921

[B107] Fernández-Martínez J. Ramírez-Casas Y. Yang Y. Aranda-Martínez P. Martínez-Ruiz L. Escames G. (2023). From chronodisruption to sarcopenia: the therapeutic potential of melatonin. Biomolecules 13 (12), 1779. 10.3390/biom13121779 38136651 PMC10741491

[B108] Ferrara-Romeo I. Martinez P. Saraswati S. Whittemore K. Graña-Castro O. Thelma Poluha L. (2020). The mTOR pathway is necessary for survival of mice with short telomeres. Nat. Commun. 11 (1), 1168. 10.1038/s41467-020-14962-1 32127537 PMC7054554

[B109] Filipski E. Lévi F. (2009). Circadian disruption in experimental cancer processes. Integr. Cancer Ther. 8 (4), 298–302. 10.1177/1534735409352085 20042408

[B110] Finger A.-M. Jäschke S. Del Olmo M. Hurwitz R. Granada A. E. Herzel H. (2021). Intercellular coupling between peripheral circadian oscillators by TGF-β signaling. Sci. Adv. 7 (30), eabg5174. 10.1126/sciadv.abg5174 34301601 PMC8302137

[B111] Fitzgerald E. S. Stout J. C. Glikmann-Johnston Y. Anderson C. Jackson M. L. (2023). Sleep, circadian rhythms, and cognitive dysfunction in Huntington’s disease. J. Huntingt. Dis. 12 (3), 293–304. 10.3233/JHD-230578 37599535

[B112] Flatt T. Schmidt P. S. (2009). Integrating evolutionary and molecular genetics of aging. Biochimica Biophysica Acta (BBA) - General Subj. 1790 (10), 951–962. 10.1016/j.bbagen.2009.07.010 19619612 PMC2972575

[B113] Fonseca Costa S. S. Ripperger J. A. (2015). Impact of the circadian clock on the aging process. Front. Neurology 6, 43. 10.3389/fneur.2015.00043 25798127 PMC4351613

[B114] Franceschi C. Campisi J. (2014). Chronic inflammation (inflammaging) and its potential contribution to age-associated diseases. Journals Gerontology. Ser. A, Biol. Sci. Med. Sci. 69 (Suppl. 1), S4–9. 10.1093/gerona/glu057 24833586

[B115] Freyberg Z. McCarthy M. J. (2017). Dopamine D2 receptors and the circadian clock reciprocally mediate antipsychotic drug-induced metabolic disturbances. npj Schizophr. 3 (1), 17. 10.1038/s41537-017-0018-4 28560263 PMC5441531

[B116] Frøland Steindal I. A. Beale A. D. Yamamoto Y. Whitmore D. (2018). Development of the Astyanax mexicanus circadian clock and non-visual light responses. Dev. Biol. 441 (2), 345–354. 10.1016/j.ydbio.2018.06.008 29909064 PMC6141809

[B117] Gabriel B. M. Zierath J. R. (2019). Circadian rhythms and exercise — re-setting the clock in metabolic disease. Nat. Rev. Endocrinol. 15 (4), 197–206. 10.1038/s41574-018-0150-x 30655625

[B118] Gao H. Xiong X. Lin Y. Chatterjee S. Ma K. (2020). The clock regulator Bmal1 protects against muscular dystrophy. Exp. Cell Res. 397 (1), 112348. 10.1016/j.yexcr.2020.112348 33130178 PMC9030224

[B119] Garatachea N. Pareja-Galeano H. Sanchis-Gomar F. Santos-Lozano A. Fiuza-Luces C. Morán M. (2015). Exercise Attenuates the Major hallmarks of aging. Rejuvenation Res. 18 (1), 57–89. 10.1089/rej.2014.1623 25431878 PMC4340807

[B120] Garaulet M. Lopez-Minguez J. Dashti H. S. Vetter C. Hernández-Martínez A. M. Pérez-Ayala M. (2022). Interplay of dinner timing and *MTNR1B* type 2 diabetes risk variant on glucose tolerance and insulin secretion: a randomized crossover trial. Diabetes Care 45 (3), 512–519. 10.2337/dc21-1314 35015083 PMC8918262

[B121] Garcia-Saenz A. de Miguel A. S. Espinosa A. Costas L. Aragonés N. Tonne C. (2020). Association between outdoor light-at-night exposure and colorectal cancer in Spain. Epidemiology 31 (5), 718–727. 10.1097/EDE.0000000000001226 32639250

[B122] Gaudreau H. Morettini J. Lavoie H. B. Carrier J. (2001). Effects of a 25-h sleep deprivation on daytime sleep in the middle-aged. Neurobiol. Aging 22 (3), 461–468. 10.1016/s0197-4580(00)00251-7 11378253

[B123] Gavriouchkina D. Fischer S. Ivacevic T. Stolte J. Benes V. Dekens M. P. S. (2010). Thyrotroph embryonic factor regulates light-induced transcription of repair genes in zebrafish embryonic cells. PLoS ONE 5 (9), e12542. 10.1371/journal.pone.0012542 20830285 PMC2935359

[B124] Geng Y.-J. Madonna R. Hermida R. C. Smolensky M. H. (2021). Pharmacogenomics and circadian rhythms as mediators of cardiovascular drug-drug interactions. Curr. Res. Pharmacol. Drug Discov. 2, 100025. 10.1016/j.crphar.2021.100025 34909660 PMC8663962

[B125] Ghosh S. Lewis K. N. Tulsian R. Astafev A. A. Buffenstein R. Kondratov R. V. (2021). It’s about time; divergent circadian clocks in livers of mice and naked mole‐rats. FASEB J. 35 (5), e21590. 10.1096/fj.202100116R 33871093 PMC9109208

[B126] Gomes A. P. Price N. L. Ling A. J. Y. Moslehi J. J. Montgomery M. K. Rajman L. (2013). Declining NAD+ induces a pseudohypoxic State disrupting nuclear-mitochondrial communication during aging. Cell 155 (7), 1624–1638. 10.1016/j.cell.2013.11.037 24360282 PMC4076149

[B127] Gómez-Santos C. Saura C. B. Lucas J. A. R. Castell P. Madrid J. A. Garaulet M. (2016). Menopause status is associated with circadian- and sleep-related alterations. Menopause (New York, N.Y.) 23 (6), 682–690. 10.1097/GME.0000000000000612 27093617

[B128] Gong X. Tang H. Yang K. (2021). PER1 suppresses glycolysis and cell proliferation in oral squamous cell carcinoma *via* the PER1/RACK1/PI3K signaling complex. Cell Death and Dis. 12 (3), 276. 10.1038/s41419-021-03563-5 33723221 PMC7960720

[B129] Gonzalez B. C. Worsaae K. Fontaneto D. Martínez A. (2018). Anophthalmia and elongation of body appendages in cave scale worms (Annelida: aphroditiformia). Zool. Scr. 47 (1), 106–121. 10.1111/zsc.12258

[B130] Gooley J. J. (2016). Circadian regulation of lipid metabolism. Proc. Nutr. Soc. 75 (4), 440–450. 10.1017/S0029665116000288 27225642

[B131] Gooley J. J. Chua E.C.-P. (2014). Diurnal regulation of lipid metabolism and applications of circadian lipidomics. J. Genet. Genomics = Yi Chuan Xue Bao 41 (5), 231–250. 10.1016/j.jgg.2014.04.001 24894351

[B132] Griffin P. Sheehan P. W. Dimitry J. M. Guo C. Kanan M. F. Lee J. (2020). REV-ERBα mediates complement expression and diurnal regulation of microglial synaptic phagocytosis. eLife 9, e58765. 10.7554/eLife.58765 33258449 PMC7728439

[B134] Gu Y. Seong D. H. Liu W. Wang Z. Jeong Y. W. Kim J. C. (2024). Exercise improves muscle mitochondrial dysfunction-associated lipid profile under circadian rhythm disturbance. Korean J. Physiology and Pharmacol. 28 (6), 515–526. 10.4196/kjpp.2024.28.6.515 39467715 PMC11519723

[B135] Guan D. Xiong Y. Trinh T. M. Xiao Y. Hu W. Jiang C. (2020). The hepatocyte clock and feeding control chronophysiology of multiple liver cell types. Science 369 (6509), 1388–1394. 10.1126/science.aba8984 32732282 PMC7849028

[B136] Hadi F. Smith E.St.J. Khaled W. T. (2021). “Naked mole-rats: resistant to developing cancer or good at avoiding it?,” in The extraordinary biology of the naked mole-rat. Editors Buffenstein R. Park T. J. Holmes M. M. (Cham: Springer International Publishing), 341–352. 10.1007/978-3-030-65943-1_14 34424524

[B137] Hagiwara A. Cornu M. Cybulski N. Polak P. Betz C. Trapani F. (2012). Hepatic mTORC2 activates glycolysis and lipogenesis through Akt, glucokinase, and SREBP1c. Cell Metab. 15 (5), 725–738. 10.1016/j.cmet.2012.03.015 22521878

[B138] Hahad O. Kuntic M. Al-Kindi S. Kuntic I. Gilan D. Petrowski K. (2025). Noise and mental health: evidence, mechanisms, and consequences. J. Expo. Sci. and Environ. Epidemiol. 35 (1), 16–23. 10.1038/s41370-024-00642-5 38279032 PMC11876073

[B139] Hämmerlein A. Derendorf H. Lowenthal D. T. (1998). Pharmacokinetic and pharmacodynamic changes in the elderly. Clinical implications. Clin. Pharmacokinet. 10.2165/00003088-199835010-00004 9673834

[B140] Hanahan D. (2022). Hallmarks of cancer: new dimensions. Cancer Discov. 12 (1), 31–46. 10.1158/2159-8290.CD-21-1059 35022204

[B141] Harrison D. E. Strong R. Sharp Z. D. Nelson J. F. Astle C. M. Flurkey K. (2010). Rapamycin fed late in life extends lifespan in genetically heterogeneous mice. Nature 460, 392–395. 10.1038/nature08221 19587680 PMC2786175

[B142] He H. Yang Y. Wang L. Guo Z. Ye L. Ou-Yang W. (2023). Combined analysis of single-cell and bulk RNA sequencing reveals the expression patterns of circadian rhythm disruption in the immune microenvironment of Alzheimer’s disease. Front. Immunol. 14, 1182307. 10.3389/fimmu.2023.1182307 37251379 PMC10213546

[B143] Hemmer A. Mareschal J. Dibner C. Pralong J. A. Dorribo V. Perrig S. (2021). The effects of shift work on cardio-metabolic diseases and eating patterns. Nutrients 13 (11), 4178. 10.3390/nu13114178 34836433 PMC8617838

[B144] Hermida R. C. Ayala D. E. Mojón A. Fernández J. R. (2011). Influence of time of day of blood pressure–lowering treatment on cardiovascular risk in hypertensive patients with type 2 diabetes. Diabetes Care 34 (6), 1270–1276. 10.2337/dc11-0297 21617110 PMC3114338

[B145] Hernandez-Morante J. J. Gomez-Santos C. Margareto J. Formiguera X. Martínez C. M. González R. (2012). Influence of menopause on adipose tissue clock gene genotype and its relationship with metabolic syndrome in morbidly obese women. Age Dordr. Neth. 34 (6), 1369–1380. 10.1007/s11357-011-9309-2 21898035 PMC3528363

[B146] Hirano A. Yumimoto K. Tsunematsu R. Matsumoto M. Oyama M. Kozuka-Hata H. (2013). FBXL21 regulates oscillation of the circadian clock through ubiquitination and stabilization of cryptochromes. Cell 152 (5), 1106–1118. 10.1016/j.cell.2013.01.054 23452856

[B147] Hoffman A. E. Zheng T. Yi C. H. Stevens R. G. Ba Y. Zhang Y. (2010). The Core circadian gene *Cryptochrome 2* influences breast cancer risk, possibly by mediating hormone signaling. Cancer Prev. Res. 3 (4), 539–548. 10.1158/1940-6207.CAPR-09-0127 20233903 PMC3175631

[B148] Hood S. Amir S. (2017). The aging clock: circadian rhythms and later life. J. Clin. Investigation 127 (2), 437–446. 10.1172/JCI90328 28145903 PMC5272178

[B149] Hou Y. Dan X. Babbar M. Wei Y. Hasselbalch S. G. Croteau D. L. (2019). Ageing as a risk factor for neurodegenerative disease. Nat. Rev. Neurol. 15 (10), 565–581. 10.1038/s41582-019-0244-7 31501588

[B150] Howarth F. G. Moldovan O. T. (2018). “The ecological classification of Cave animals and their adaptations,” in Cave ecology. Editors Moldovan O. T. Kováč Ľ. Halse S. (Cham: Springer International Publishing), 41–67. 10.1007/978-3-319-98852-8_4

[B151] Huang W. Y. Feng J. Zheng C. Jiao J. Wong S. H. S. (2024). Associations of social jetlag with physical activity and sedentary behaviour in children and adolescents: a systematic review and meta‐analysis. J. Sleep Res. 33 (1), e13997. 10.1111/jsr.13997 37443521

[B152] Hussain Y. Dar M. I. Pan X. (2024). Circadian influences on brain lipid metabolism and neurodegenerative diseases. Metabolites 14 (12), 723. 10.3390/metabo14120723 39728504 PMC11677446

[B153] Imai S. Guarente L. (2014). NAD+ and sirtuins in aging and disease. Trends Cell Biol. 24 (8), 464–471. 10.1016/j.tcb.2014.04.002 24786309 PMC4112140

[B154] Ishikawa H. Hoshino T. Hamanaka G. Mandeville E. T. Guo S. Kimura S. (2025). Effects of aging on diurnal transcriptome change in the mouse corpus callosum. iScience 28 (1), 111556. 10.1016/j.isci.2024.111556 39845418 PMC11750567

[B155] Iskusnykh I. Y. Zakharova A. A. Kryl'skii E. D. Popova T. N. (2024). Aging, neurodegenerative disorders, and cerebellum. Int. J. Mol. Sci. 25 (2), 1018. 10.3390/ijms25021018 38256091 PMC10815822

[B156] Iweka C. A. Seigneur E. Hernandez A. L. Paredes S. H. Cabrera M. Blacher E. (2023). Myeloid deficiency of the intrinsic clock protein BMAL1 accelerates cognitive aging by disrupting microglial synaptic pruning. J. Neuroinflammation 20 (1), 48. 10.1186/s12974-023-02727-8 36829230 PMC9951430

[B157] Jabbur M. L. Johnson C. H. (2022). Spectres of clock evolution: past, present, and yet to come. Front. Physiology 12, 815847. 10.3389/fphys.2021.815847 35222066 PMC8874327

[B158] Jana T. Tzveta S. Zlatina N. Natasha I. Dimitrinka A. Milena A. (2020). Effect of endurance training on diurnal rhythms of superoxide dismutase activity, glutathione and lipid peroxidation in plasma of pinealectomized rats. Neurosci. Lett. 716, 134637. 10.1016/j.neulet.2019.134637 31751669

[B159] Jansen E. C. Dolinoy D. Peterson K. E. O’Brien L. M. Chervin R. D. Cantoral A. (2021). Adolescent sleep timing and dietary patterns in relation to DNA methylation of core circadian genes: a pilot study of Mexican youth. Epigenetics 16 (8), 894–907. 10.1080/15592294.2020.1827719 33016191 PMC8331002

[B160] Jarvis J. U. M. (1981). Eusociality in a mammal: cooperative breeding in naked mole-rat colonies. Science 212 (4494), 571–573. 10.1126/science.7209555 7209555

[B161] Jeffery W. R. (2009). Regressive evolution in *Astyanax* cavefish. Annu. Rev. Genet. 43 (1), 25–47. 10.1146/annurev-genet-102108-134216 19640230 PMC3594788

[B162] Jiang W. Zhao S. Jiang X. Zhang E. Hu G. Hu B. (2016). The circadian clock gene Bmal1 acts as a potential anti-oncogene in pancreatic cancer by activating the p53 tumor suppressor pathway. Cancer Lett. 371 (2), 314–325. 10.1016/j.canlet.2015.12.002 26683776

[B163] Jiang H. Yang X. Mi M. Wei X. Wu H. Xin Y. (2021). PER2: a potential molecular marker for hematological malignancies. Mol. Biol. Rep. 48 (11), 7587–7595. 10.1007/s11033-021-06751-w 34642831

[B164] Jiang Z. Zou K. Liu X. Gu H. Meng Y. Lin J. (2021). Aging attenuates the ovarian circadian rhythm. J. Assisted Reproduction Genet. 38 (1), 33–40. 10.1007/s10815-020-01943-y 32926298 PMC7822988

[B165] Joyce D. S. Feigl B. Kerr G. Roeder L. Zele A. J. (2018). Melanopsin-mediated pupil function is impaired in Parkinson’s disease. Sci. Rep. 8 (1), 7796. 10.1038/s41598-018-26078-0 29773814 PMC5958070

[B166] Jung‐Hynes B. Reiter R. J. Ahmad N. (2010). Sirtuins, melatonin and circadian rhythms: building a bridge between aging and cancer. J. Pineal Res. 48 (1), 9–19. 10.1111/j.1600-079X.2009.00729.x 20025641 PMC2948667

[B167] Juste Y. R. Kaushik S. Bourdenx M. Aflakpui R. Bandyopadhyay S. Garcia F. (2021). Reciprocal regulation of chaperone-mediated autophagy and the circadian clock. Nat. Cell Biol. 23 (12), 1255–1270. 10.1038/s41556-021-00800-z 34876687 PMC8688252

[B168] Kanan M. F. Sheehan P. W. Haines J. N. Gomez P. G. Dhuler A. Nadarajah C. J. (2024). Neuronal deletion of the circadian clock gene Bmal1 induces cell-autonomous dopaminergic neurodegeneration. JCI Insight 9 (2), e162771. 10.1172/jci.insight.162771 38032732 PMC10906231

[B169] Kang J.-E. Lim M. M. Bateman R. J. Lee J. J. Smyth L. P. Cirrito J. R. (2009). Amyloid-β dynamics are regulated by orexin and the sleep-wake cycle. Science 326 (5955), 1005–1007. 10.1126/science.1180962 19779148 PMC2789838

[B170] Kara N. Iweka C. A. Blacher E. (2023). Chrono‐Gerontology: integrating circadian rhythms and aging in stroke research. Adv. Biol. 7 (11), 2300048. 10.1002/adbi.202300048 37409422

[B171] Karasek M. (2004). Melatonin, human aging, and age-related diseases. Exp. Gerontol. 39 (11–12), 1723–1729. 10.1016/j.exger.2004.04.012 15582288

[B172] Kawinska A. Dumont M. Selmaoui B. Paquet J. Carrier J. (2005). Are modifications of melatonin circadian rhythm in the middle years of life related to habitual patterns of light exposure? J. Biol. Rhythms 20 (5), 451–460. 10.1177/0748730405280248 16267384

[B173] Keane M. Semeiks J. Webb A. E. Li Y. I. Quesada V. Craig T. (2015). Insights into the evolution of longevity from the bowhead whale genome. Cell Rep. 10 (1), 112–122. 10.1016/j.celrep.2014.12.008 25565328 PMC4536333

[B174] Kemler D. Wolff C. A. Esser K. A. (2020). Time‐of‐day dependent effects of contractile activity on the phase of the skeletal muscle clock. J. Physiology 598 (17), 3631–3644. 10.1113/JP279779 32537739 PMC7479806

[B175] Kenyon C. (2011). The first long-lived mutants: discovery of the insulin/IGF-1 pathway for ageing. Philosophical Trans. R. Soc. Lond. Ser. B, Biol. Sci. 366 (1561), 9–16. 10.1098/rstb.2010.0276 21115525 PMC3001308

[B177] Khapre R. V. Kondratova A. A. Patel S. Dubrovsky Y. Wrobel M. Antoch M. P. (2014a). BMAL1-dependent regulation of the mTOR signaling pathway delays aging. Aging 6 (1), 48–57. 10.18632/aging.100633 24481314 PMC3927809

[B178] Khapre R. V. Patel S. A. Kondratova A. A. Chaudhary A. Velingkaar N. Antoch M. P. (2014b). Metabolic clock generates nutrient anticipation rhythms in mTOR signaling. Aging 6 (8), 675–689. 10.18632/aging.100686 25239872 PMC4169861

[B179] Kiessling S. Beaulieu-Laroche L. Blum I. D. Landgraf D. Welsh D. K. Storch K. F. (2017). Enhancing circadian clock function in cancer cells inhibits tumor growth. BMC Biol. 15 (1), 13. 10.1186/s12915-017-0349-7 28196531 PMC5310078

[B180] Kim J. I. Cheon H. G. (2024). Melatonin ameliorates hepatic fibrosis *via* the melatonin receptor 2-mediated upregulation of BMAL1 and anti-oxidative enzymes. Eur. J. Pharmacol. 966, 176337. 10.1016/j.ejphar.2024.176337 38246330

[B181] Kim E. B. Fang X. Fushan A. A. Huang Z. Lobanov A. V. Han L. (2011). Genome sequencing reveals insights into physiology and longevity of the naked mole rat. Nature 479 (7372), 223–227. 10.1038/nature10533 21993625 PMC3319411

[B182] Kim H.-K. Radak Z. Takahashi M. Inami T. Shibata S. (2023). Chrono-exercise: Time-of-day-dependent physiological responses to exercise. Sports Med. Health Sci. 5 (1), 50–58. 10.1016/j.smhs.2022.11.003 36994180 PMC10040331

[B183] Kobuchi S. Yazaki Y. Ito Y. Sakaeda T. (2018). Circadian variations in the pharmacokinetics of capecitabine and its metabolites in rats. Eur. J. Pharm. Sci. 112, 152–158. 10.1016/j.ejps.2017.11.021 29175408

[B184] Koike N. Yoo S. H. Huang H. C. Kumar V. Lee C. Kim T. K. (2012). Transcriptional Architecture and chromatin landscape of the core circadian clock in mammals. Science 338 (6105), 349–354. 10.1126/science.1226339 22936566 PMC3694775

[B185] Kok E. Y. Kaur S. Mohd Shukri N. H. Abdul Razak N. Takahashi M. Teoh S. C. (2024). The role of light exposure in infant circadian rhythm establishment: a scoping review perspective. Eur. J. Pediatr. 184 (1), 112. 10.1007/s00431-024-05951-3 39738921 PMC11685245

[B187] Kolinjivadi A. M. Chong S. T. Ngeow J. (2021). Molecular connections between circadian rhythm and genome maintenance pathways. Endocrine-Related Cancer 28 (2), R55–R66. 10.1530/ERC-20-0372 33300498

[B188] Kondratov R. V. Kondratova A. A. Gorbacheva V. Y. Vykhovanets O. V. Antoch M. P. (2006). Early aging and age-related pathologies in mice deficient in BMAL1, the core componentof the circadian clock. Genes and Dev. 20 (14), 1868–1873. 10.1101/gad.1432206 16847346 PMC1522083

[B189] Kondratov R. V. Vykhovanets O. Kondratova A. A. Antoch M. P. (2009). Antioxidant N-acetyl-L-cysteine ameliorates symptoms of premature aging associated with the deficiency of the circadian protein BMAL1. Aging 1 (12), 979–987. 10.18632/aging.100113 20157581 PMC2815755

[B190] Kou L. Chi X. Sun Y. Han C. Wan F. Hu J. (2022). The circadian clock protein Rev-erbα provides neuroprotection and attenuates neuroinflammation against Parkinson’s disease *via* the microglial NLRP3 inflammasome. J. Neuroinflammation 19 (1), 133. 10.1186/s12974-022-02494-y 35668454 PMC9169406

[B191] Kritsilis M. V Rizou S. Koutsoudaki P. N. Evangelou K. Gorgoulis V. G. Papadopoulos D. (2018). Ageing, cellular senescence and neurodegenerative disease. Int. J. Mol. Sci. 19 (10), 2937. 10.3390/ijms19102937 30261683 PMC6213570

[B192] Kroemer G. Maier A. B. Cuervo A. M. Gladyshev V. N. Ferrucci L. Gorbunova V. (2025). From geroscience to precision geromedicine: understanding and managing aging. Cell 188 (8), 2043–2062. 10.1016/j.cell.2025.03.011 40250404 PMC12037106

[B193] Kunieda T. Minamino T. Miura K. Katsuno T. Tateno K. Miyauchi H. (2008). Reduced nitric oxide causes age-associated impairment of circadian rhythmicity. Circulation Res. 102 (5), 607–614. 10.1161/CIRCRESAHA.107.162230 18218984

[B194] Kwapis J. L. Alaghband Y. Kramár E. A. López A. J. Vogel Ciernia A. White A. O. (2018). Epigenetic regulation of the circadian gene Per1 contributes to age-related changes in hippocampal memory. Nat. Commun. 9 (1), 3323. 10.1038/s41467-018-05868-0 30127461 PMC6102273

[B195] Laermans J. Depoortere I. (2016). Chronobesity: role of the circadian system in the obesity epidemic. Obes. Rev. 17 (2), 108–125. 10.1111/obr.12351 26693661

[B196] Lages M. Carmo-Silva S. Barros R. Guarino M. P. (2024). Effects of time-restricted eating on body composition, biomarkers of metabolism, inflammation, circadian system and oxidative stress in overweight and obesity: an exploratory review. Proc. Nutr. Soc., 1–10. 10.1017/S002966512400747X 39563167

[B197] Lamia K. A. Sachdeva U. M. DiTacchio L. Williams E. C. Alvarez J. G. Egan D. F. (2009). AMPK regulates the circadian clock by cryptochrome phosphorylation and degradation. Science 326 (5951), 437–440. 10.1126/science.1172156 19833968 PMC2819106

[B198] Lananna B. V. Musiek E. S. (2020). The wrinkling of time: aging, inflammation, oxidative stress, and the circadian clock in neurodegeneration. Neurobiol. Dis. 139, 104832. 10.1016/j.nbd.2020.104832 32179175 PMC7727873

[B199] Landolt H. P. Dijk D. J. Achermann P. Borbély A. A. (1996). Effect of age on the sleep EEG: slow-wave activity and spindle frequency activity in young and middle-aged men. Brain Res. 738 (2), 205–212. 10.1016/s0006-8993(96)00770-6 8955514

[B200] Lecour S. Du Pré B. C. Bøtker H. E. Brundel B. J. J. M. Daiber A. Davidson S. M. (2022). Circadian rhythms in ischaemic heart disease: key aspects for preclinical and translational research: position paper of the ESC working group on cellular biology of the heart. Cardiovasc. Res. 118 (12), 2566–2581. 10.1093/cvr/cvab293 34505881

[B201] Lee J. Dimitry J. M. Song J. H. Son M. Sheehan P. W. King M. W. (2023). Microglial REV-ERBα regulates inflammation and lipid droplet formation to drive tauopathy in male mice. Nat. Commun. 14 (1), 5197. 10.1038/s41467-023-40927-1 37626048 PMC10457319

[B202] Leskinen T. Suorsa K. Heinonen I. H. Löyttyniemi E. Pentti J. Vahtera J. (2021). The effect of commercial activity tracker based physical activity intervention on body composition and cardiometabolic health among recent retirees. Front. Aging 2, 757080. 10.3389/fragi.2021.757080 35822058 PMC9261302

[B203] Lévi F. Okyar A. Dulong S. Innominato P. F. Clairambault J. (2010). Circadian timing in cancer treatments. Annu. Rev. Pharmacol. Toxicol. 50 (1), 377–421. 10.1146/annurev.pharmtox.48.113006.094626 20055686

[B204] Lewy A. J. Wehr T. A. Goodwin F. K. Newsome D. A. Markey S. P. (1980). Light suppresses melatonin secretion in humans. Science 210 (4475), 1267–1269. 10.1126/science.7434030 7434030

[B205] Li X. Fu B. Zhao C. Hu J. Zhang X. Fu Y. (2023). Early-life noise exposure causes cognitive impairment in a sex-dependent manner by disrupting homeostasis of the microbiota–gut–brain axis. Brain, Behav. Immun. 114, 221–239. 10.1016/j.bbi.2023.08.021 37648006

[B206] Liang S. Mele J. Wu Y. Buffenstein R. Hornsby P. J. (2010). Resistance to experimental tumorigenesis in cells of a long‐lived mammal, the naked mole‐rat (*Heterocephalus glaber*). Aging Cell 9 (4), 626–635. 10.1111/j.1474-9726.2010.00588.x 20550519 PMC3743245

[B207] Liang C. Liu Z. Song M. Li W. Wu Z. Wang Z. (2021). Stabilization of heterochromatin by CLOCK promotes stem cell rejuvenation and cartilage regeneration. Cell Res. 31 (2), 187–205. 10.1038/s41422-020-0385-7 32737416 PMC8027439

[B208] Liguori I. Russo G. Curcio F. Bulli G. Aran L. Della-Morte D. (2018). Oxidative stress, aging, and diseases. Clin. Interventions Aging 13, 757–772. 10.2147/CIA.S158513 29731617 PMC5927356

[B209] Limson J. Nyokong T. Daya S. (1998). The interaction of melatonin and its precursors with aluminium, cadmium, copper, iron, lead, and zinc: an adsorptive voltammetric study. J. Pineal Res. 24 (1), 15–21. 10.1111/j.1600-079X.1998.tb00361.x 9468114

[B210] Linsell C. R. Lightman S. L. Mullen P. E. Brown M. J. Causon R. C. (1985). Circadian rhythms of epinephrine and norepinephrine in man. J. Clin. Endocrinol. and Metabolism 60 (6), 1210–1215. 10.1210/jcem-60-6-1210 3998066

[B211] Lipton J. O. Boyle L. M. Yuan E. D. Hochstrasser K. J. Chifamba F. F. Nathan A. (2017). Aberrant proteostasis of BMAL1 underlies circadian abnormalities in a paradigmatic mTOR-opathy. Cell Rep. 20 (4), 868–880. 10.1016/j.celrep.2017.07.008 28746872 PMC5603761

[B212] Liu Y. Jiang H. (2025). The impact of social isolation on subjective cognitive decline in older adults: a Study based on network analysis and longitudinal model. arXiv. 10.48550/ARXIV.2506.13914

[B213] Liu J. A. Bumgarner J. R. Walker W. H. 2nd Meléndez-Fernández O. H. Walton J. C. DeVries A. C. (2024). Chronic phase advances reduces recognition memory and increases vascular cognitive dementia-like impairments in aged mice. Sci. Rep. 14 (1), 7760. 10.1038/s41598-024-57511-2 38565934 PMC10987525

[B428] Logan R. W. McClung C. A. (2019). Rhythms of life: circadian disruption and brain disorders across the lifespan. Nat. Rev. Neurosci. 20 (1), 49–65. 30459365 10.1038/s41583-018-0088-y PMC6338075

[B214] Lok R. Ancoli-Israel S. Ensrud K. E. Redline S. Stone K. L. Zeitzer J. M. (2023). Timing of outdoor light exposure is associated with sleep-wake consolidation in community-dwelling older men. Front. Sleep 2, 1268379. 10.3389/frsle.2023.1268379 PMC12713814 41426466

[B215] Longo V. D. Panda S. (2016). Fasting, circadian rhythms, and time-restricted feeding in healthy lifespan. Cell Metab. 23 (6), 1048–1059. 10.1016/j.cmet.2016.06.001 27304506 PMC5388543

[B216] López-Otín C. Kroemer G. (2021). Hallmarks of health. Cell 184 (1), 33–63. 10.1016/j.cell.2020.11.034 33340459

[B217] López-Otín C. Blasco M. A. Partridge L. Serrano M. Kroemer G. (2013). The hallmarks of aging. Cell 153 (6), 1194–1217. 10.1016/j.cell.2013.05.039 23746838 PMC3836174

[B218] López-Otín C. Blasco M. A. Partridge L. Serrano M. Kroemer G. (2023a). Hallmarks of aging: an expanding universe. Cell 186 (2), 243–278. 10.1016/j.cell.2022.11.001 36599349

[B219] López-Otín C. Pietrocola F. Roiz-Valle D. Galluzzi L. Kroemer G. (2023b). Meta-hallmarks of aging and cancer. Cell Metab. 35 (1), 12–35. 10.1016/j.cmet.2022.11.001 36599298

[B220] Lunghi E. Bilandžija H. (2022). Longevity in Cave animals. Front. Ecol. Evol. 10, 874123. 10.3389/fevo.2022.874123

[B221] Lunghi E. Zhao Y. (2020). Do Chinese cavefish show intraspecific variability in morphological traits? Ecol. Evol. 10 (14), 7723–7730. 10.1002/ece3.6495 32760559 PMC7391565

[B222] Lunghi E. Manenti R. Ficetola G. F. (2015). Seasonal variation in microhabitat of salamanders: environmental variation or shift of habitat selection? PeerJ 3, e1122. 10.7717/peerj.1122 26290788 PMC4540018

[B223] Ma S. Upneja A. Galecki A. Tsai Y. M. Burant C. F. Raskind S. (2016). Cell culture-based profiling across mammals reveals DNA repair and metabolism as determinants of species longevity. eLife 5, e19130. 10.7554/eLife.19130 27874830 PMC5148604

[B224] Ma L. Yu F. He D. Guo L. Yang Y. Li W. (2023). Role of circadian clock in the chronoefficacy and chronotoxicity of clopidogrel. Br. J. Pharmacol. 180 (23), 2973–2988. 10.1111/bph.16188 37403641

[B225] Ma Z. Jiang W. Zhang E. E. (2016). Orexin signaling regulates both the hippocampal clock and the circadian oscillation of Alzheimer’s disease-risk genes. Sci. Rep. 6 (1), 36035. 10.1038/srep36035 27796320 PMC5086843

[B226] Magnusdottir S. Magnusdottir I. Gunnlaugsdottir A. K. Hilmisson H. Hrolfsdottir L. Paed A. E. E. M. (2024). Sleep duration and social jetlag in healthy adolescents. Association with anxiety, depression, and chronotype: a pilot study. Sleep Breath. 28 (4), 1541–1551. 10.1007/s11325-024-03026-z 38546939

[B227] Makarem N. Sears D. D. St-Onge M. P. Zuraikat F. M. Gallo L. C. Talavera G. A. (2021). Variability in daily eating patterns and eating Jetlag are associated with worsened cardiometabolic risk profiles in the American heart Association Go red for women strategically focused research network. J. Am. Heart Assoc. 10 (18), e022024. 10.1161/JAHA.121.022024 34482703 PMC8649529

[B228] Makris K. C. Heibati B. Narui S. Z. (2023). Chrono-modulated effects of external stressors on oxidative stress and damage in humans: a scoping review on night shift work. Environ. Int. 178, 108048. 10.1016/j.envint.2023.108048 37463540

[B229] Mammola S. (2019). Finding answers in the dark: caves as models in ecology fifty years after Poulson and White. Ecography 42 (7), 1331–1351. 10.1111/ecog.03905

[B230] Mason I. C. Qian J. Adler G. K. Scheer F. A. J. L. (2020). Impact of circadian disruption on glucose metabolism: implications for type 2 diabetes. Diabetologia 63 (3), 462–472. 10.1007/s00125-019-05059-6 31915891 PMC7002226

[B231] Massudi H. Grant R. Braidy N. Guest J. Farnsworth B. Guillemin G. J. (2012). Age-Associated changes in oxidative stress and NAD+ metabolism in human tissue. PLoS ONE 7 (7), e42357. 10.1371/journal.pone.0042357 22848760 PMC3407129

[B232] Masuda S. Narasimamurthy R. Yoshitane H. Kim J. K. Fukada Y. Virshup D. M. (2020). Mutation of a PER2 phosphodegron perturbs the circadian phosphoswitch. Proc. Natl. Acad. Sci. 117 (20), 10888–10896. 10.1073/pnas.2000266117 32354999 PMC7245125

[B233] Masuda K. Katsuda Y. Niwa Y. Sakurai T. Hirano A. (2023). Analysis of circadian rhythm components in EEG/EMG data of aged mice. Front. Neurosci. 17, 1173537. 10.3389/fnins.2023.1173537 37250413 PMC10213445

[B234] Maury E. (2019). Off the clock: from circadian disruption to Metabolic disease. Int. J. Mol. Sci. 20 (7), 1597. 10.3390/ijms20071597 30935034 PMC6480015

[B235] Mauvoisin D. Wang J. Jouffe C. Martin E. Atger F. Waridel P. (2014). Circadian clock-dependent and -independent rhythmic proteomes implement distinct diurnal functions in mouse liver. Proc. Natl. Acad. Sci. 111 (1), 167–172. 10.1073/pnas.1314066111 24344304 PMC3890886

[B236] Mehta D. Rajput K. Jain D. Bajaj A. Dasgupta U. (2024). Unveiling the role of mechanistic target of Rapamycin Kinase (MTOR) signaling in cancer progression and the emergence of MTOR inhibitors as therapeutic strategies. ACS Pharmacol. and Transl. Sci. 7 (12), 3758–3779. 10.1021/acsptsci.4c00530 39698262 PMC11650738

[B237] Meyer N. Lok R. Schmidt C. Kyle S. D. McClung C. A. Cajochen C. (2024). The sleep–circadian interface: a window into mental disorders. Proc. Natl. Acad. Sci. 121 (9), e2214756121. 10.1073/pnas.2214756121 38394243 PMC10907245

[B238] Milan M. Brown J. O'Reilly C. L. Bubak M. P. Negri S. Balasubramanian P. (2024). Time-restricted feeding improves aortic endothelial relaxation by enhancing mitochondrial function and attenuating oxidative stress in aged mice. Redox Biol. 73, 103189. 10.1016/j.redox.2024.103189 38788541 PMC11140804

[B239] Milczarek R. Hallmann A. Sokołowska E. Kaletha K. Klimek J. (2010). Melatonin enhances antioxidant action of α-tocopherol and ascorbate against NADPH- and iron-dependent lipid peroxidation in human placental mitochondria: antioxidant action of melatonin in human placenta. J. Pineal Res. 49, 149–155. 10.1111/j.1600-079X.2010.00779.x 20524970

[B240] Miller C. L. White R. Whitman T. L. O’Callaghan M. F. Maxwell S. E. (1995). The effects of cycled *versus* noncycled lighting on growth and development in preterm infants. Infant Behav. Dev. 18 (1), 87–95. 10.1016/0163-6383(95)90010-1

[B241] Miller R. A. Harrison D. E. Astle C. M. Baur J. A. Boyd A. R. de Cabo R. (2011). Rapamycin, but not resveratrol or Simvastatin, extends life span of genetically heterogeneous mice. Journals Gerontology Ser. A 66A (2), 191–201. 10.1093/gerona/glq178 20974732 PMC3021372

[B242] Miller K. N. Victorelli S. G. Salmonowicz H. Dasgupta N. Liu T. Passos J. F. (2021). Cytoplasmic DNA: sources, sensing, and role in aging and disease. Cell 184 (22), 5506–5526. 10.1016/j.cell.2021.09.034 34715021 PMC8627867

[B243] Milosic F. Hengstschläger M. Osmanagic-Myers S. (2024). Premature aging in genetic diseases: what conclusions can be drawn for physiological aging. Front. Aging 4, 1327833. 10.3389/fragi.2023.1327833 38481648 PMC10933081

[B244] Minges K. E. Redeker N. S. (2016). Delayed school start times and adolescent sleep: a systematic review of the experimental evidence. Sleep. Med. Rev. 28, 86–95. 10.1016/j.smrv.2015.06.002 26545246 PMC4844764

[B245] Mohammadpour Fard R. Rashno M. Bahreiny S. S. (2024). Effects of melatonin supplementation on markers of inflammation and oxidative stress in patients with diabetes: a systematic review and meta-analysis of randomized controlled trials. Clin. Nutr. ESPEN 63, 530–539. 10.1016/j.clnesp.2024.07.015 39053698

[B246] Montazid S. Bandyopadhyay S. Hart D. W. Gao N. Johnson B. Thrumurthy S. G. (2023). Adult stem cell activity in naked mole rats for long-term tissue maintenance. Nat. Commun. 14 (1), 8484. 10.1038/s41467-023-44138-6 38123565 PMC10733326

[B247] Montégut L. Abdellatif M. Motiño O. Madeo F. Martins I. Quesada V. (2023). Acyl coenzyme A binding protein (ACBP): an aging‐ and disease‐relevant “autophagy checkpoint”. Aging Cell 22 (9), e13910. 10.1111/acel.13910 37357988 PMC10497816

[B248] Moqrich A. (2014). Peripheral pain-sensing neurons: from molecular diversity to functional specialization. Cell Rep. 6 (2), 245–246. 10.1016/j.celrep.2014.01.018 24484769

[B249] Moran D. Softley R. Warrant E. J. (2014). Eyeless Mexican cavefish save energy by eliminating the circadian rhythm in metabolism. PLoS ONE 9 (9), e107877. 10.1371/journal.pone.0107877 25251018 PMC4176717

[B250] Morris C. J. Purvis T. E. Hu K. Scheer F. A. J. L. (2016). Circadian misalignment increases cardiovascular disease risk factors in humans. Proc. Natl. Acad. Sci. 113 (10), E1402–E1411. 10.1073/pnas.1516953113 26858430 PMC4790999

[B251] Mortimer T. Zinna V. M. Atalay M. Laudanna C. Deryagin O. Posas G. (2024). The epidermal circadian clock integrates and subverts brain signals to guarantee skin homeostasis. Cell Stem Cell 31 (6), 834–849.e4. 10.1016/j.stem.2024.04.013 38701785

[B252] Mortimer T. Smith J. G. Muñoz-Cánoves P. Benitah S. A. (2025). Circadian clock communication during homeostasis and ageing. Nat. Rev. Mol. Cell Biol. 26 (4), 314–331. 10.1038/s41580-024-00802-3 39753699

[B253] Mukherji A. Bailey S. M. Staels B. Baumert T. F. (2019). The circadian clock and liver function in health and disease. J. Hepatology 71 (1), 200–211. 10.1016/j.jhep.2019.03.020 30930223 PMC7613420

[B254] Mulder C. K. Gerkema M. P. Van Der Zee E. A. (2013). Circadian clocks and memory: time-place learning. Front. Mol. Neurosci. 6, 8. 10.3389/fnmol.2013.00008 23596390 PMC3622895

[B255] Murgo E. Colangelo T. Bellet M. M. Malatesta F. Mazzoccoli G. (2023). Role of the circadian gas-responsive hemeprotein NPAS2 in physiology and pathology. Biology 12 (10), 1354. 10.3390/biology12101354 37887064 PMC10603908

[B256] Musiek E. S. Holtzman D. M. (2016). Mechanisms linking circadian clocks, sleep, and neurodegeneration. Science 354 (6315), 1004–1008. 10.1126/science.aah4968 27885006 PMC5219881

[B257] Nahmod N. G. Master L. Chang A. M. Hale L. Buxton O. M. (2019). Later high school start times associated with longer actigraphic sleep duration in adolescents. Sleep 42 (2), zsy212. 10.1093/sleep/zsy212 30395345 PMC6369724

[B258] Nakahata Y. Kaluzova M. Grimaldi B. Sahar S. Hirayama J. Chen D. (2008). The NAD+-Dependent deacetylase SIRT1 modulates CLOCK-Mediated chromatin remodeling and circadian control. Cell 134 (2), 329–340. 10.1016/j.cell.2008.07.002 18662547 PMC3526943

[B259] Nakahata Y. Sahar S. Astarita G. Kaluzova M. Sassone-Corsi P. (2009). Circadian control of the NAD^+^ salvage pathway by CLOCK-SIRT1. Science 324 (5927), 654–657. 10.1126/science.1170803 19286518 PMC6501775

[B260] Nakajima K. Suwa K. Toyama K. (2017). Age-dependent changes in the association between sleep duration and impaired glucose metabolism. World J. Diabetes 8 (8), 397–406. 10.4239/wjd.v8.i8.397 28861177 PMC5561039

[B261] Nakamura T. J. Nakamura W. Yamazaki S. Kudo T. Cutler T. Colwell C. S. (2011). Age-Related decline in circadian output. J. Neurosci. 31 (28), 10201–10205. 10.1523/JNEUROSCI.0451-11.2011 21752996 PMC3155746

[B262] Nakamura T. J. Nakamura W. Tokuda I. T. Ishikawa T. Kudo T. Colwell C. S. (2015). Age-Related changes in the circadian System unmasked by constant conditions. eneuro 2 (4), ENEURO.0064-15. 10.1523/ENEURO.0064-15.2015 26464996 PMC4596014

[B263] Nakamura T. J. Takasu N. N. Nakamura W. (2016). The suprachiasmatic nucleus: age-related decline in biological rhythms. J. Physiological Sci. 66 (5), 367–374. 10.1007/s12576-016-0439-2 26915078 PMC10717791

[B264] Natarajan A. Pantelopoulos A. Emir-Farinas H. Natarajan P. (2020). Heart rate variability with photoplethysmography in 8 million individuals: a cross-sectional study. Lancet Digital Health 2 (12), e650–e657. 10.1016/S2589-7500(20)30246-6 33328029

[B265] Nguyen Ho P. T. Hoepel S. J. W. Rodriguez-Ayllon M. Luik A. I. Vernooij M. W. Neitzel J. (2024). Sleep, 24-Hour activity rhythms, and subsequent Amyloid-β pathology. JAMA Neurol. 81 (8), 824–834. 10.1001/jamaneurol.2024.1755 38913396 PMC11197458

[B266] Niu L. Zhang F. Xu X. Yang Y. Li S. Liu H. (2022). Chronic sleep deprivation altered the expression of circadian clock genes and aggravated Alzheimer’s disease neuropathology. Brain Pathol. 32 (3), e13028. 10.1111/bpa.13028 34668266 PMC9048513

[B267] Nojima A. Yamashita M. Yoshida Y. Shimizu I. Ichimiya H. Kamimura N. (2013). Haploinsufficiency of Akt1 prolongs the lifespan of mice. PLoS ONE 8 (7), e69178. 10.1371/journal.pone.0069178 23935948 PMC3728301

[B268] North B. J. Sinclair D. A. (2012). The intersection between aging and cardiovascular disease. Circulation Res. 110 (8), 1097–1108. 10.1161/CIRCRESAHA.111.246876 22499900 PMC3366686

[B269] Oh G. Koncevičius K. Ebrahimi S. Carlucci M. Groot D. E. Nair A. (2019). Circadian oscillations of cytosine modification in humans contribute to epigenetic variability, aging, and complex disease. Genome Biol. 20 (1), 2. 10.1186/s13059-018-1608-9 30606238 PMC6317262

[B270] Ohdo S. (2010). Chronotherapeutic strategy: rhythm monitoring, manipulation and disruption. Adv. Drug Deliv. Rev. 62 (9–10), 859–875. 10.1016/j.addr.2010.01.006 20188774

[B271] Oka K. Yamakawa M. Kawamura Y. Kutsukake N. Miura K. (2023). The naked mole-rat as a model for healthy aging. Annu. Rev. Animal Biosci. 11 (1), 207–226. 10.1146/annurev-animal-050322-074744 36318672

[B272] Ono D. Honma K. Honma S. (2021). Roles of neuropeptides, VIP and AVP, in the Mammalian central circadian clock. Front. Neurosci. 15, 650154. 10.3389/fnins.2021.650154 33935635 PMC8081951

[B273] Orozco-Solis R. Aguilar-Arnal L. (2020). Circadian regulation of immunity through epigenetic mechanisms. Front. Cell. Infect. Microbiol. 10, 96. 10.3389/fcimb.2020.00096 32232012 PMC7082642

[B274] Ozturk N. Ozturk D. Kavakli I. H. Okyar A. (2017). Molecular aspects of circadian pharmacology and relevance for cancer chronotherapy. Int. J. Mol. Sci. 18 (10), 2168. 10.3390/ijms18102168 29039812 PMC5666849

[B275] Pacheco-Bernal I. Becerril-Pérez F. Aguilar-Arnal L. (2019). Circadian rhythms in the three-dimensional genome: implications of chromatin interactions for cyclic transcription. Clin. Epigenetics 11 (1), 79. 10.1186/s13148-019-0677-2 31092281 PMC6521413

[B276] Palmer J. E. Wilson N. Son S. M. Obrocki P. Wrobel L. Rob M. (2025). Autophagy, aging, and age-related neurodegeneration. Neuron 113 (1), 29–48. 10.1016/j.neuron.2024.09.015 39406236

[B277] Palomar-Cros A. Deprato A. Papantoniou K. Straif K. Lacy P. Maidstone R. (2024). Indoor and outdoor artificial light-at-night (ALAN) and cancer risk: a systematic review and meta-analysis of multiple cancer sites and with a critical appraisal of exposure assessment. Sci. Total Environ. 955, 177059. 10.1016/j.scitotenv.2024.177059 39437923

[B278] Park T. J. Lu Y. Jüttner R. Smith E. S. J. Hu J. Brand A. (2008). Selective inflammatory pain insensitivity in the African naked mole-rat (Heterocephalus glaber). PLoS Biol. 6 (1), e13. 10.1371/journal.pbio.0060013 18232734 PMC2214810

[B279] Paschos G. K. FitzGerald G. A. (2010). Circadian clocks and vascular function. Circulation Res. 106 (5), 833–841. 10.1161/CIRCRESAHA.109.211706 20299673 PMC2848505

[B280] Pascual-Torner M. Carrero D. Pérez-Silva J. G. Álvarez-Puente D. Roiz-Valle D. Bretones G. (2022). Comparative genomics of mortal and immortal cnidarians unveils novel keys behind rejuvenation. Proc. Natl. Acad. Sci. 119 (36), e2118763119. 10.1073/pnas.2118763119 36037356 PMC9459311

[B281] Patke A. Young M. W. Axelrod S. (2020). Molecular mechanisms and physiological importance of circadian rhythms. Nat. Rev. Mol. Cell Biol. 21 (2), 67–84. 10.1038/s41580-019-0179-2 31768006

[B282] Pazan F. Wehling M. (2021). Polypharmacy in older adults: a narrative review of definitions, epidemiology and consequences. Eur. Geriatr. Med. 12 (3), 443–452. 10.1007/s41999-021-00479-3 33694123 PMC8149355

[B283] Peek C. B. (2020). Metabolic implications of Circadian–HIF crosstalk. Trends Endocrinol. and Metabolism 31 (6), 459–468. 10.1016/j.tem.2020.02.008 32396846

[B284] Peichl L. Němec P. Burda H. (2004). Unusual cone and rod properties in subterranean African mole‐rats (Rodentia, Bathyergidae). Eur. J. Neurosci. 19 (6), 1545–1558. 10.1111/j.1460-9568.2004.03263.x 15066151

[B285] Pendergrast L. A. Ashcroft S. P. Ehrlich A. M. Treebak J. T. Krook A. Dollet L. (2024). Metabolic plasticity and obesity-associated changes in diurnal postexercise metabolism in mice. Metabolism 155, 155834. 10.1016/j.metabol.2024.155834 38479569

[B286] Penev P. D. Kolker D. E. Zee P. C. Turek F. W. (1998). Chronic circadian desynchronization decreases the survival of animals with cardiomyopathic heart disease. Am. J. Physiology-Heart Circulatory Physiology 275 (6), H2334–H2337. 10.1152/ajpheart.1998.275.6.H2334 9843836

[B287] Peterson B. A. Kennedy B. J. (1979). Aging and cancer management part I: clinical observations. CA A Cancer J. Clin. 29 (6), 322–332. 10.3322/canjclin.29.6.322 116720

[B288] Petrenko V. Sinturel F. Riezman H. Dibner C. (2023). Lipid metabolism around the body clocks. Prog. Lipid Res. 91, 101235. 10.1016/j.plipres.2023.101235 37187314

[B289] Poggiogalle E. Jamshed H. Peterson C. M. (2018). Circadian regulation of glucose, lipid, and energy metabolism in humans. Metabolism 84, 11–27. 10.1016/j.metabol.2017.11.017 29195759 PMC5995632

[B290] Polinski J. M. Zimin A. V. Clark K. F. Kohn A. B. Sadowski N. Timp W. (2021). The American lobster genome reveals insights on longevity, neural, and immune adaptations. Sci. Adv. 7 (26), eabe8290. 10.1126/sciadv.abe8290 34162536 PMC8221624

[B291] Pompeia S. Panjeh S. Louzada F. M. D’Almeida V. Hipolide D. C. Cogo-Moreira H. (2023). Social jetlag is associated with adverse cardiometabolic latent traits in early adolescence: an observational study. Front. Endocrinol. 14, 1085302. 10.3389/fendo.2023.1085302 37469985 PMC10352840

[B292] Poulson T. L. (1963). Cave adaptation in amblyopsid fishes. Am. Midl. Nat. 70 (2), 257. 10.2307/2423056

[B293] Puppala A. Rankawat S. Ray S. (2021). Circadian timekeeping in anticancer therapeutics: an emerging vista of chronopharmacology research. Curr. Drug Metab. 22 (13), 998–1008. 10.2174/1389200222666211119103422 34802402

[B294] Qian J. Scheer F. A. J. L. (2016). Circadian System and glucose metabolism: implications for physiology and disease. Trends Endocrinol. and Metabolism 27 (5), 282–293. 10.1016/j.tem.2016.03.005 27079518 PMC4842150

[B295] Rae D. E. Tomaz S. A. Jones R. A. Hinkley T. Twine R. Kahn K. (2021). Sleep and BMI in South African urban and rural, high and low-income preschool children. BMC Public Health 21 (1), 571. 10.1186/s12889-021-10591-5 33757479 PMC7986550

[B296] Rahman S. A. Gathungu R. M. Marur V. R. St Hilaire M. A. Scheuermaier K. Belenky M. (2023). Age-related changes in circadian regulation of the human plasma lipidome. Commun. Biol. 6 (1), 756. 10.1038/s42003-023-05102-8 37474677 PMC10359364

[B297] Rakshit K. Thomas A. P. Matveyenko A. V. (2014). Does disruption of circadian rhythms contribute to beta-cell failure in type 2 diabetes? Curr. Diabetes Rep. 14 (4), 474. 10.1007/s11892-014-0474-4 24532160 PMC3988110

[B298] Ramanathan C. Kathale N. D. Liu D. Lee C. Freeman D. A. Hogenesch J. B. (2018). mTOR signaling regulates central and peripheral circadian clock function. PLOS Genet. 14 (5), e1007369. 10.1371/journal.pgen.1007369 29750810 PMC5965903

[B299] Ramasubbu K. Devi Rajeswari V. (2023). Impairment of insulin signaling pathway PI3K/Akt/mTOR and insulin resistance induced AGEs on diabetes mellitus and neurodegenerative diseases: a perspective review. Mol. Cell. Biochem. 478 (6), 1307–1324. 10.1007/s11010-022-04587-x 36308670

[B300] Rangel-Zuñiga O. A. Cruz-Teno C. Haro C. Quintana-Navarro G. M. Camara-Martos F. Perez-Martinez P. (2017). Differential menopause-*versus* aging-induced changes in oxidative stress and circadian rhythm gene markers. Mech. Ageing Dev. 164, 41–48. 10.1016/j.mad.2017.04.002 28408140

[B301] Ren J. Hu D. Mao Y. Yang H. Liao W. Xu W. (2019). Alteration in gut microbiota caused by time‐restricted feeding alleviate hepatic ischaemia reperfusion injury in mice. J. Cell. Mol. Med. 23 (3), 1714–1722. 10.1111/jcmm.14069 30588757 PMC6378231

[B302] Ren X. Wang W. Li W. Sun L. Liu T. Zhou H. (2025). Circadian rest-activity rhythms and multimorbidity and mortality risks among menopausal women: a trajectory analysis of a UK Biobank cohort. BMC public health 25 (1), 1304. 10.1186/s12889-025-22536-3 40197377 PMC11974044

[B303] Roberts N. T. MacDonald C. R. Mohammadpour H. Antoch M. P. Repasky E. A. (2022). Circadian rhythm disruption increases tumor growth rate and accumulation of myeloid-derived suppressor cells. Adv. Biol. 6 (9), 2200031. 10.1002/adbi.202200031 35652494 PMC9474681

[B304] Roenneberg T. (2023). How can social jetlag affect health? Nat. Rev. Endocrinol. 19 (7), 383–384. 10.1038/s41574-023-00851-2 37221400 PMC10204006

[B305] Roenneberg T. Merrow M. (2016). The circadian clock and human health. Curr. Biol. 26 (10), R432–R443. 10.1016/j.cub.2016.04.011 27218855

[B306] Roenneberg T. Allebrandt K. V. Merrow M. Vetter C. (2012). Social Jetlag and obesity. Curr. Biol. 22 (10), 939–943. 10.1016/j.cub.2012.03.038 22578422

[B427] Ruan W. Yuan X. Eltzschig H. K. (2021). Circadian rhythm as a therapeutic target. Nat. Rev. Drug Discov. 20 (4), 287–307. 33589815 10.1038/s41573-020-00109-w PMC8525418

[B307] Ruby J. G. Smith M. Buffenstein R. (2018). Naked mole-rat mortality rates defy Gompertzian laws by not increasing with age. eLife 7, e31157. 10.7554/eLife.31157 29364116 PMC5783610

[B308] Saade-Lemus S. Videnovic A. (2023). Sleep disorders and circadian disruption in Huntington’s disease. J. Huntingt. Dis. 12 (2), 121–131. 10.3233/JHD-230576 37424473 PMC10473087

[B309] Saeed Y. Abbott S. M. (2017). Circadian disruption associated with alzheimer’s disease. Curr. Neurology Neurosci. Rep. 17 (4), 29. 10.1007/s11910-017-0745-y 28324298

[B310] Sakamoto W. Takenoshita S. (2015). Overexpression of both clock and bmal1 inhibits entry to s phase in human colon cancer cells. Fukushima J. Med. Sci. 61 (2), 111–124. 10.5387/fms.2015-11 26370682 PMC5131586

[B311] Salamanca-Fernández E. Rodríguez-Barranco M. Guevara M. Ardanaz E. Olry de Labry Lima A. Sánchez M. J. (2018). Night-shift work and breast and prostate cancer risk: updating the evidence from epidemiological studies. An. del Sist. Sanit. Navar. 41 (2), 211–226. 10.23938/ASSN.0307 30063040

[B312] Sancar A. Van Gelder R. N. (2021). Clocks, cancer, and chronochemotherapy. Science 371 (6524), eabb0738. 10.1126/science.abb0738 33384351

[B313] Santoni M. Molina-Cerrillo J. Santoni G. Lam E. T. Massari F. Mollica V. (2023). Role of clock genes and circadian rhythm in renal cell carcinoma: recent evidence and therapeutic consequences. Cancers 15 (2), 408. 10.3390/cancers15020408 36672355 PMC9856936

[B314] Sato S. Solanas G. Peixoto F. O. Bee L. Symeonidi A. Schmidt M. S. (2017). Circadian reprogramming in the liver identifies metabolic pathways of aging. Cell 170 (4), 664–677.e11. 10.1016/j.cell.2017.07.042 28802039 PMC7792549

[B315] Sato S. Basse A. L. Schönke M. Chen S. Samad M. Altıntaş A. (2019). Time of exercise specifies the impact on muscle metabolic pathways and systemic energy homeostasis. Cell Metab. 30 (1), 92–110.e4. 10.1016/j.cmet.2019.03.013 31006592

[B316] Sato S. Dyar K. A. Treebak J. T. Jepsen S. L. Ehrlich A. M. Ashcroft S. P. (2022). Atlas of exercise metabolism reveals time-dependent signatures of metabolic homeostasis. Cell Metab. 34 (2), 329–345.e8. 10.1016/j.cmet.2021.12.016 35030324 PMC13189211

[B317] Savvidis C. Koutsilieris M. (2012). Circadian rhythm disruption in cancer biology. Mol. Med. 18 (9), 1249–1260. 10.2119/molmed.2012.00077 22811066 PMC3521792

[B318] Schurhoff N. Toborek M. (2023). Circadian rhythms in the blood–brain barrier: impact on neurological disorders and stress responses. Mol. Brain 16 (1), 5. 10.1186/s13041-023-00997-0 36635730 PMC9835375

[B319] Seim I. Ma S. Zhou X. Gerashchenko M. V. Lee S. G. Suydam R. (2014). The transcriptome of the bowhead whale Balaena mysticetus reveals adaptations of the longest-lived mammal. Aging 6 (10), 879–899. 10.18632/aging.100699 25411232 PMC4247388

[B320] Seluanov A. Gladyshev V. N. Vijg J. Gorbunova V. (2018). Mechanisms of cancer resistance in long-lived mammals. Nat. Rev. Cancer 18 (7), 433–441. 10.1038/s41568-018-0004-9 29622806 PMC6015544

[B321] Sen S. K. Wang L. Brown M. Wu J. Wei Z. Matveyenko A. (2023). 1773-P: Glucocorticoid receptor signaling regulates pancreatic beta-cell circadian transcriptome and function. Diabetes 72 (Suppl_1), 1773. 10.2337/db23-1773-P

[B322] Seney M. L. Cahill K. Enwright J. F. 3rd Logan R. W. Huo Z. Zong W. (2019). Diurnal rhythms in gene expression in the prefrontal cortex in schizophrenia. Nat. Commun. 10 (1), 3355. 10.1038/s41467-019-11335-1 31399567 PMC6689017

[B323] Shen Y. Lv Q. K. Xie W. Y. Gong S. Y. Zhuang S. Liu J. Y. (2023). Circadian disruption and sleep disorders in neurodegeneration. Transl. Neurodegener. 12 (1), 8. 10.1186/s40035-023-00340-6 36782262 PMC9926748

[B324] Shen W. Cai L. Li J. Sun Y. Wang B. Wang N. (2024). Association of night shift work and biological ageing: the mediating role of body mass index. Age Ageing 53 (11), afae242. 10.1093/ageing/afae242 39497270

[B325] Shepard A. Kissil J. L. (2020). The use of non-traditional models in the study of cancer resistance—the case of the naked mole rat. Oncogene 39 (28), 5083–5097. 10.1038/s41388-020-1355-8 32535616

[B326] Shimba S. Ogawa T. Hitosugi S. Ichihashi Y. Nakadaira Y. Kobayashi M. (2011). Deficient of a clock gene, brain and muscle arnt-like Protein-1 (BMAL1), induces dyslipidemia and ectopic fat Formation. PLoS ONE 6 (9), e25231. 10.1371/journal.pone.0025231 21966465 PMC3178629

[B327] Sies H. (2018). On the history of oxidative stress: concept and some aspects of current development. Curr. Opin. Toxicol. 7, 122–126. 10.1016/j.cotox.2018.01.002

[B328] Smarr B. L. Grant A. D. Perez L. Zucker I. Kriegsfeld L. J. (2017). Maternal and early-life circadian disruption have long-lasting negative consequences on offspring development and adult behavior in mice. Sci. Rep. 7 (1), 3326. 10.1038/s41598-017-03406-4 28607386 PMC5468226

[B329] Song F. Walker M. P. (2023). Sleep, alcohol, and caffeine in financial traders. PLOS ONE 18 (11), e0291675. 10.1371/journal.pone.0291675 37939019 PMC10631622

[B330] Stafford M. Gardner M. Kumari M. Kuh D. Ben-Shlomo Y. (2013). Social isolation and diurnal cortisol patterns in an ageing cohort. Psychoneuroendocrinology 38 (11), 2737–2745. 10.1016/j.psyneuen.2013.07.002 23920224 PMC3820041

[B331] Stanton D. Justin H. S. Reitzel A. M. (2022). Step in time: conservation of circadian clock genes in animal evolution. Integr. Comp. Biol. 62 (6), 1503–1518. 10.1093/icb/icac140 36073444

[B332] Staples A. D. Hoyniak C. McQuillan M. E. Molfese V. Bates J. E. (2021). Screen use before bedtime: consequences for nighttime sleep in young children. Infant Behav. Dev. 62, 101522. 10.1016/j.infbeh.2020.101522 33385752 PMC7977486

[B333] Stenbäck V. Mutt S. J. Leppäluoto J. Gagnon D. D. Mäkelä K. A. Jokelainen J. (2019). Association of physical activity with telomere length among elderly Adults—The oulu cohort 1945. Front. Physiology 10, 444. 10.3389/fphys.2019.00444 31105579 PMC6499171

[B334] Stephenson E. M. Usselmann L. E. J. Tergaonkar V. Virshup D. M. Dallmann R. (2021). Cancer clocks in tumourigenesis: the p53 pathway and beyond. Endocrine-Related Cancer 28 (4), R95–R110. 10.1530/ERC-20-0475 33638942

[B335] Stevens R. G. Zhu Y. (2015). Electric light, particularly at night, disrupts human circadian rhythmicity: is that a problem? Philosophical Trans. R. Soc. B Biol. Sci. 370 (1667), 20140120. 10.1098/rstb.2014.0120 25780233 PMC4375361

[B336] Stewart J. Bachman G. Cooper C. Liu L. Ancoli-Israel S. Alibiglou L. (2018). Circadian dysfunction and fluctuations in gait initiation impairment in Parkinson’s disease. Exp. Brain Res. 236 (3), 655–664. 10.1007/s00221-017-5163-5 29294143

[B337] Straif K. Baan R. Grosse Y. Secretan B. El Ghissassi F. Bouvard V. (2007). Carcinogenicity of shift-work, painting, and fire-fighting. Lancet Oncol. 8 (12), 1065–1066. 10.1016/S1470-2045(07)70373-X 19271347

[B338] Suknovic N. Tomczyk S. Colevret D. Perruchoud C. Galliot B. (2021). The ULK1 kinase, a necessary component of the pro-regenerative and anti-aging machinery in Hydra. Mech. Ageing Dev. 194, 111414. 10.1016/j.mad.2020.111414 33338499

[B339] Sulli G. Lam M. T. Y. Panda S. (2019). Interplay between circadian clock and cancer: new frontiers for cancer treatment. Trends Cancer 5 (8), 475–494. 10.1016/j.trecan.2019.07.002 31421905 PMC7120250

[B340] Sun Y. Jin L. Sui Y. X. Han L. L. Liu J. H. (2017). Circadian gene CLOCK affects drug-resistant gene expression and cell proliferation in ovarian cancer SKOV3/DDP cell lines through autophagy. Cancer Biotherapy Radiopharm. 32 (4), 139–146. 10.1089/cbr.2016.2153 28514207

[B341] Swan J. A. Golden S. S. LiWang A. Partch C. L. (2018). Structure, function, and mechanism of the core circadian clock in Cyanobacteria. J. Biol. Chem. 293 (14), 5026–5034. 10.1074/jbc.TM117.001433 29440392 PMC5892564

[B342] Szewczyk‐Golec K. Woźniak A. Reiter R. J. (2015). Inter‐relationships of the chronobiotic, melatonin, with leptin and adiponectin: implications for obesity. J. Pineal Res. 59 (3), 277–291. 10.1111/jpi.12257 26103557

[B344] Takeda N. Maemura K. (2015). The role of clock genes and circadian rhythm in the development of cardiovascular diseases. Cell. Mol. Life Sci. 72 (17), 3225–3234. 10.1007/s00018-015-1923-1 25972277 PMC11113935

[B345] Takeda N. Maemura K. (2016). Circadian clock and the onset of cardiovascular events. Hypertens. Res. 39 (6), 383–390. 10.1038/hr.2016.9 26888119

[B346] Tamai T. K. Vardhanabhuti V. Foulkes N. S. Whitmore D. (2004). Early embryonic light detection improves survival. Curr. Biol. 14 (5), 446. 10.1016/j.cub.2004.02.040 14986634

[B347] Telzer E. H. Goldenberg D. Fuligni A. J. Lieberman M. D. Gálvan A. (2015). Sleep variability in adolescence is associated with altered brain development. Dev. Cogn. Neurosci. 14, 16–22. 10.1016/j.dcn.2015.05.007 26093368 PMC4536158

[B348] Thacher J. D. Snigireva A. Dauter U. M. Delaval M. N. Oudin A. Mattisson K. (2024). Road traffic noise and breast cancer: DNA methylation in four core circadian genes. Clin. Epigenetics 16 (1), 168. 10.1186/s13148-024-01774-z 39587706 PMC11590349

[B349] Thaiss C. A. Zeevi D. Levy M. Zilberman-Schapira G. Suez J. Tengeler A. C. (2014). Transkingdom control of microbiota diurnal oscillations promotes metabolic homeostasis. Cell 159 (3), 514–529. 10.1016/j.cell.2014.09.048 25417104

[B350] Thome-Souza S. Klehm J. Jackson M. Kadish N. E. Manganaro S. Fernández I. S. (2016). Clobazam higher-evening differential dosing as an add-on therapy in refractory epilepsy. Seizure 40, 1–6. 10.1016/j.seizure.2016.05.014 27281712

[B351] Thosar S. S. Butler M. P. Shea S. A. (2018). Role of the circadian system in cardiovascular disease. J. Clin. Investigation 128 (6), 2157–2167. 10.1172/JCI80590 29856365 PMC5983320

[B352] Toiber D. Erdel F. Bouazoune K. Silberman D. M. Zhong L. Mulligan P. (2013). SIRT6 recruits SNF2H to DNA break sites, preventing genomic instability through chromatin remodeling. Mol. Cell 51 (4), 454–468. 10.1016/j.molcel.2013.06.018 23911928 PMC3761390

[B353] Torquati L. Mielke G. I. Brown W. J. Kolbe-Alexander T. (2018). Shift work and the risk of cardiovascular disease. A systematic review and meta-analysis including dose–response relationship. Scand. J. Work, Environ. and Health 44 (3), 229–238. 10.5271/sjweh.3700 29247501

[B354] Tran H. T. Kondo T. Ashry A. Fu Y. Okawa H. Sawangmake C. (2024). Effect of circadian clock disruption on type 2 diabetes. Front. Physiology 15, 1435848. 10.3389/fphys.2024.1435848 39165284 PMC11333352

[B355] Tsioufis C. Syrseloudis D. Dimitriadis K. Thomopoulos C. Tsiachris D. Pavlidis P. (2008). Disturbed circadian blood pressure rhythm and C-reactive protein in essential hypertension. J. Hum. Hypertens. 22 (7), 501–508. 10.1038/jhh.2008.20 18385743

[B356] Tsurudome Y. Akamine T. Horiguchi M. Wada Y. Fujimura A. Ushijima K. (2022). Potential mechanism of hepatic lipid accumulation during a long-term rest phase restricted feeding in mice. Chronobiology Int. 39 (8), 1132–1143. 10.1080/07420528.2022.2077746 35603436

[B357] Türk D. Scherer N. Selzer D. Dings C. Hanke N. Dallmann R. (2023). Significant impact of time-of-day variation on metformin pharmacokinetics. Diabetologia 66 (6), 1024–1034. 10.1007/s00125-023-05898-4 36930251 PMC10163090

[B358] Uzu T. Kimura G. Yamauchi A. Kanasaki M. Isshiki K. Araki S. i. (2006). Enhanced sodium sensitivity and disturbed circadian rhythm of blood pressure in essential hypertension. J. Hypertens. 24 (8), 1627–1632. 10.1097/01.hjh.0000239299.71001.77 16877966

[B359] Van Gilst D. Puchkina A. V. Roelants J. A. Kervezee L. Dudink J. Reiss I. K. M. (2023). Effects of the neonatal intensive care environment on circadian health and development of preterm infants. Front. Physiology 14, 1243162. 10.3389/fphys.2023.1243162 37719464 PMC10500197

[B360] Varcoe T. J. Boden M. J. Voultsios A. Salkeld M. D. Rattanatray L. Kennaway D. J. (2013). Characterisation of the maternal response to chronic phase shifts during gestation in the rat: implications for fetal metabolic programming. PLoS ONE 8 (1), e53800. 10.1371/journal.pone.0053800 23342007 PMC3544759

[B361] Veilleux A. Houde V. P. Bellmann K. Marette A. (2010). Chronic inhibition of the mTORC1/S6K1 pathway increases insulin-induced PI3K activity but inhibits Akt2 and glucose transport stimulation in 3T3-L1 adipocytes. Mol. Endocrinol. Baltim. Md. 24 (4), 766–778. 10.1210/me.2009-0328 20203102 PMC5417537

[B362] Verde L. Barrea L. Vetrani C. Frias-Toral E. Chapela S. P. Jayawardena R. (2022). Chronotype and sleep quality in obesity: how do they change after menopause? Curr. Obes. Rep. 11 (4), 254–262. 10.1007/s13679-022-00479-9 36053414 PMC9729134

[B363] Verlande A. Masri S. (2019). Circadian clocks and cancer: timekeeping governs cellular metabolism. Trends Endocrinol. and Metabolism 30 (7), 445–458. 10.1016/j.tem.2019.05.001 31155396 PMC6679985

[B364] Verma A. K. Khan M. I. Ashfaq F. Rizvi S. I. (2023). Crosstalk between aging, circadian rhythm, and Melatonin. Rejuvenation Res. 26 (6), 229–241. 10.1089/rej.2023.0047 37847148

[B365] Videnovic A. Zee P. C. (2015). Consequences of circadian disruption on neurologic health. Sleep. Med. Clin. 10 (4), 469–480. 10.1016/j.jsmc.2015.08.004 26568123 PMC4648713

[B366] Vinogradova I. A. Bukalev A. V. Zabezhinski M. A. Semenchenko A. V. Khavinson V. K. Anisimov V. N. (2007). Effect of ala-glu-asp-gly peptide on life span and development of spontaneous tumors in female rats exposed to different illumination regimes. Bull. Exp. Biol. Med. 144 (6), 825–830. 10.1007/s10517-007-0441-z 18856211

[B367] Vinogradova I. A. Anisimov V. N. Bukalev A. V. Semenchenko A. V. Zabezhinski M. A. (2009). Circadian disruption induced by light-at-night accelerates aging and promotes tumorigenesis in rats. Aging 1 (10), 855–865. 10.18632/aging.100092 20157558 PMC2816394

[B368] Vinogradova I. A. Anisimov V. N. Bukalev A. V. Ilyukha V. A. Khizhkin E. A. Lotosh T. A. (2010). Circadian disruption induced by light-at-night accelerates aging and promotes tumorigenesis in young but not in old rats. Aging 2 (2), 82–92. 10.18632/aging.100120 20354269 PMC2850144

[B369] Vlachou D. Veretennikova M. Usselmann L. Vasilyev V. Ott S. Bjarnason G. A. (2024). TimeTeller: a tool to probe the circadian clock as a multigene dynamical system. PLOS Comput. Biol. 20 (2), e1011779. 10.1371/journal.pcbi.1011779 38422117 PMC10931517

[B370] Voituron Y. de Fraipont M. Issartel J. Guillaume O. Clobert J. (2011). Extreme lifespan of the human fish (*Proteus anguinus*): a challenge for ageing mechanisms. Biol. Lett. 7 (1), 105–107. 10.1098/rsbl.2010.0539 20659920 PMC3030882

[B371] Vujović N. Piron M. J. Qian J. Chellappa S. L. Nedeltcheva A. Barr D. (2022). Late isocaloric eating increases hunger, decreases energy expenditure, and modifies metabolic pathways in adults with overweight and obesity. Cell Metab. 34 (10), 1486–1498.e7. 10.1016/j.cmet.2022.09.007 36198293 PMC10184753

[B372] Wallace N. K. Pollard F. Savenkova M. Karatsoreos I. N. (2020). Effect of aging on daily rhythms of lactate metabolism in the medial prefrontal cortex of Male mice. Neuroscience 448, 300–310. 10.1016/j.neuroscience.2020.07.032 32717298 PMC7584730

[B373] Wang X. Yu W. Zheng L. (2015). The dynamics of NF-κB pathway regulated by circadian clock. Math. Biosci. 260, 47–53. 10.1016/j.mbs.2014.07.012 25172045

[B374] Wang J. Mauvoisin D. Martin E. Atger F. Galindo A. N. Dayon L. (2017). Nuclear proteomics uncovers diurnal regulatory landscapes in mouse liver. Cell Metab. 25 (1), 102–117. 10.1016/j.cmet.2016.10.003 27818260 PMC5241201

[B375] Wang J. Li S. Li X. Li B. Li Y. Xia K. (2019). Circadian protein BMAL1 promotes breast cancer cell invasion and metastasis by up-regulating matrix metalloproteinase9 expression. Cancer Cell Int. 19 (1), 182. 10.1186/s12935-019-0902-2 31346317 PMC6636133

[B376] Wang C. Zhang Y. Cao J. Yang Z. (2025). Oscillatory dynamics and regulatory mechanisms of the p53–Per2 network in DNA-Damaged cells. IEEE Trans. Neural Netw. Learn. Syst. 36 (5), 9725–9732. 10.1109/TNNLS.2024.3424784 39058613

[B377] Wang J. Shao F. Yu Q. X. Ye L. Wusiman D. Wu R. (2025). The common hallmarks and interconnected pathways of aging, circadian rhythms, and cancer: implications for therapeutic strategies. Res. Wash. D.C. 8, 0612. 10.34133/research.0612 40046513 PMC11880593

[B378] Wang L. Cui C. Y. Lee C. T. Bodogai M. Yang N. Shi C. (2025). Spatial transcriptomics of the aging mouse brain reveals origins of inflammation in the white matter. Nat. Commun. 16 (1), 3231. 10.1038/s41467-025-58466-2 40185750 PMC11971433

[B379] Weger B. D. Sahinbas M. Otto G. W. Mracek P. Armant O. Dolle D. (2011). The light responsive transcriptome of the zebrafish: function and regulation. PLoS ONE 6 (2), e17080. 10.1371/journal.pone.0017080 21390203 PMC3039656

[B380] Weger B. D. Gobet C. David F. P. A. Atger F. Martin E. Phillips N. E. (2021). Systematic analysis of differential rhythmic liver gene expression mediated by the circadian clock and feeding rhythms. Proc. Natl. Acad. Sci. 118 (3), e2015803118. 10.1073/pnas.2015803118 33452134 PMC7826335

[B381] Wei F. Chen W. Lin X. (2022). Night-shift work, breast cancer incidence, and all-cause mortality: an updated meta-analysis of prospective cohort studies. Sleep Breath. 26 (4), 1509–1526. 10.1007/s11325-021-02523-9 34775538

[B382] Weichhart T. (2018). mTOR as regulator of lifespan, aging, and cellular senescence: a mini-review. Gerontology 64 (2), 127–134. 10.1159/000484629 29190625 PMC6089343

[B383] Welz P.-S. Benitah S. A. (2020). Molecular connections between circadian clocks and aging. J. Mol. Biol. 432 (12), 3661–3679. 10.1016/j.jmb.2019.12.036 31887285

[B384] Westhoff M. Del Villar S. G. Voelker T. L. Thai P. N. Spooner H. C. Costa A. D. (2024). BIN1 knockdown rescues systolic dysfunction in aging Male mouse hearts. Nat. Commun. 15 (1), 3528. 10.1038/s41467-024-47847-8 38664444 PMC11045846

[B385] Westwood E. Smith S. Mann D. Pattinson C. Allan A. Staton S. (2023). The effects of light in children: a systematic review. J. Environ. Psychol. 89, 102062. 10.1016/j.jenvp.2023.102062

[B386] Whittaker D. S. Akhmetova L. Carlin D. Romero H. Welsh D. K. Colwell C. S. (2023). Circadian modulation by time-restricted feeding rescues brain pathology and improves memory in mouse models of Alzheimer’s disease. Cell Metab. 35 (10), 1704–1721.e6. 10.1016/j.cmet.2023.07.014 37607543 PMC10591997

[B387] Wise P. M. Cohen I. R. Weiland N. G. London E. D. (1988). Aging alters the circadian rhythm of glucose utilization in the suprachiasmatic nucleus. Proc. Natl. Acad. Sci. U. S. A. 85 (14), 5305–5309. 10.1073/pnas.85.14.5305 3393539 PMC281739

[B388] Wolff G. Esser K. A. (2012). Scheduled exercise phase shifts the circadian clock in skeletal muscle. Med. and Sci. Sports and Exerc. 44 (9), 1663–1670. 10.1249/MSS.0b013e318255cf4c 22460470 PMC3414645

[B389] Wolff C. A. Gutierrez-Monreal M. A. Meng L. Zhang X. Douma L. G. Costello H. M. (2023). Defining the age-dependent and tissue-specific circadian transcriptome in Male mice. Cell Rep. 42 (1), 111982. 10.1016/j.celrep.2022.111982 36640301 PMC9929559

[B390] Wong P. M. Hasler B. P. Kamarck T. W. Muldoon M. F. Manuck S. B. (2015). Social jetlag, chronotype, and cardiometabolic risk. J. Clin. Endocrinol. and Metabolism 100 (12), 4612–4620. 10.1210/jc.2015-2923 26580236 PMC4667156

[B391] Wu R. Dang F. Li P. Wang P. Xu Q. Liu Z. (2019). The circadian protein Period2 suppresses mTORC1 activity *via* recruiting Tsc1 to mTORC1 complex. Cell Metab. 29 (3), 653–667.e6. 10.1016/j.cmet.2018.11.006 30527742

[B392] Xie L. Kang H. Xu Q. Chen M. J. Liao Y. Thiyagarajan M. (2013). Sleep drives metabolite clearance from the adult brain. Science 342 (6156), 373–377. 10.1126/science.1241224 24136970 PMC3880190

[B393] Xie Y. Shi X. Sheng K. Han G. Li W. Zhao Q. (2019). PI3K/Akt signaling transduction pathway, erythropoiesis and glycolysis in hypoxia (review). Mol. Med. Rep. 19 (2), 783–791. 10.3892/mmr.2018.9713 30535469 PMC6323245

[B394] Xu C. Lu C. Hua L. Jin H. Yin L. Chen S. (2009). Rhythm changes of clock genes, apoptosis-related genes and atherosclerosis-related genes in apolipoprotein E knockout mice. Can. J. Cardiol. 25 (8), 473–479. 10.1016/S0828-282X(09)70122-9 19668782 PMC2732375

[B395] Xu Z. Jin J. Yang T. Wang Y. Huang J. Pan X. (2023). Outdoor light at night, genetic predisposition and type 2 diabetes mellitus: a prospective cohort study. Environ. Res. 219, 115157. 10.1016/j.envres.2022.115157 36572333

[B426] Yamaguchi O. Otsu K. (2012). Role of autophagy in aging. J. Cardiovasc. Pharmacol. 60 (3), 242–247. 22343371 10.1097/FJC.0b013e31824cc31c

[B396] Yang G. Chen L. Grant G. R. Paschos G. Song W. L. Musiek E. S. (2016). Timing of expression of the core clock gene *Bmal1* influences its effects on aging and survival. Sci. Transl. Med. 8 (324), 324ra16. 10.1126/scitranslmed.aad3305 26843191 PMC4870001

[B397] Yang Y. Yang T. Zhao Z. Zhang H. Yuan P. Wang G. (2022). Down-regulation of BMAL1 by MiR-494-3p promotes hepatocellular carcinoma growth and metastasis by increasing GPAM-Mediated lipid biosynthesis. Int. J. Biol. Sci. 18 (16), 6129–6144. 10.7150/ijbs.74951 36439870 PMC9682529

[B398] Yang Z. Black K. Ohman‐Strickland P. Graber J. M. Kipen H. M. Fang M. (2024). Disruption of central and peripheral circadian clocks and circadian controlled estrogen receptor rhythms in night shift nurses in working environments. FASEB J. 38 (11), e23719. 10.1096/fj.202302261RR 38837828 PMC11884403

[B399] Ye Z. Huang K. Dai X. Gao D. Gu Y. Qian J. (2024). Light-phase time-restricted feeding disrupts the muscle clock and insulin sensitivity yet potentially induces muscle fiber remodeling in mice. Heliyon 10 (18), e37475. 10.1016/j.heliyon.2024.e37475 39328525 PMC11425116

[B400] Yeom J. W. Park S. Lee H.-J. (2024). Managing circadian rhythms: a key to enhancing mental health in college students. Psychiatry Investig. 21 (12), 1309–1317. 10.30773/pi.2024.0250 39757810 PMC11704804

[B401] Yin D. Yu Z. Jiang H. Chong Y. Zhong C. Xu S. (2024). Circadian rhythm mechanisms underlying convergent adaptation of unihemispheric slow-wave sleep in marine mammals. Mol. Biol. Evol. 41 (12), msae257. 10.1093/molbev/msae257 39703058 PMC11683420

[B402] Yoo S.-H. Mohawk J. A. Siepka S. M. Shan Y. Huh S. K. Hong H. K. (2013). Competing E3 ubiquitin ligases govern circadian periodicity by degradation of CRY in nucleus and cytoplasm. Cell 152 (5), 1091–1105. 10.1016/j.cell.2013.01.055 23452855 PMC3694781

[B403] Yoshida K. Nakai A. Kaneshiro K. Hashimoto N. Suzuki K. Uchida K. (2018). TNF-α induces expression of the circadian clock gene Bmal1 *via* dual calcium-dependent pathways in rheumatoid synovial cells. Biochem. Biophysical Res. Commun. 495 (2), 1675–1680. 10.1016/j.bbrc.2017.12.015 29217191

[B404] Yoshizawa M. Goricki S. Soares D. Jeffery W. R. (2010). Evolution of a behavioral shift mediated by superficial neuromasts helps cavefish find food in darkness. Curr. Biol. 20 (18), 1631–1636. 10.1016/j.cub.2010.07.017 20705469 PMC2946428

[B405] Youm Y.-H. Grant R. W. McCabe L. R. Albarado D. C. Nguyen K. Y. Ravussin A. (2013). Canonical Nlrp3 inflammasome links systemic low-grade inflammation to functional decline in aging. Cell Metab. 18 (4), 519–532. 10.1016/j.cmet.2013.09.010 24093676 PMC4017327

[B406] Youm Y.-H. Nguyen K. Y. Grant R. W. Goldberg E. L. Bodogai M. Kim D. (2015). The ketone metabolite β-hydroxybutyrate blocks NLRP3 inflammasome–mediated inflammatory disease. Nat. Med. 21 (3), 263–269. 10.1038/nm.3804 25686106 PMC4352123

[B407] Yu H. Meng X. Wu J. Pan C. Ying X. Zhou Y. (2013). Cryptochrome 1 overexpression correlates with tumor progression and poor prognosis in patients with colorectal cancer. PLoS ONE 8 (4), e61679. 10.1371/journal.pone.0061679 23626715 PMC3634012

[B408] Yu Z. Seim I. Yin M. Tian R. Sun D. Ren W. (2021). Comparative analyses of aging-related genes in long-lived mammals provide insights into natural longevity. Innovation 2 (2), 100108. 10.1016/j.xinn.2021.100108 34557758 PMC8454735

[B409] Yuan G. Hua B. Cai T. Xu L. Huang Y. Sun N. (2017). Clock mediates liver senescence by controlling ER stress. Aging 9 (12), 2647–2665. 10.18632/aging.101353 29283886 PMC5764397

[B410] Zeng Z.-L. Wu M. W. Sun J. Sun Y. L. Cai Y. C. Huang Y. J. (2010). Effects of the biological clock gene Bmal1 on tumour growth and anti-cancer drug activity. J. Biochem. 148 (3), 319–326. 10.1093/jb/mvq069 20576619

[B411] Zhang Y.-K. J. Yeager R. L. Klaassen C. D. (2009). Circadian expression profiles of drug-processing genes and transcription factors in mouse liver. Drug Metabolism Dispos. 37 (1), 106–115. 10.1124/dmd.108.024174 18838502 PMC2683654

[B412] Zhang L. Ptáčk L. J. Fu Y.-H. (2015). Nuclear envelope regulates the circadian clock. Nucleus 6 (2), 114–117. 10.1080/19491034.2015.1010949 25746393 PMC4615844

[B413] Zhang W. Xiong Y. Tao R. Panayi A. C. Mi B. Liu G. (2022). Emerging insight into the role of circadian clock gene BMAL1 in cellular senescence. Front. Endocrinol. 13, 915139. 10.3389/fendo.2022.915139 35733785 PMC9207346

[B414] Zhang C. Chen L. Sun L. Jin H. Ren K. Liu S. (2023). BMAL1 collaborates with CLOCK to directly promote DNA double-strand break repair and tumor chemoresistance. Oncogene 42 (13), 967–979. 10.1038/s41388-023-02603-y 36725890 PMC10038804

[B415] Zhang X. Pant S. M. Ritch C. C. Tang H. Y. Shao H. Dweep H. (2024). Cell state dependent effects of Bmal1 on melanoma immunity and tumorigenicity. Nat. Commun. 15 (1), 633. 10.1038/s41467-024-44778-2 38245503 PMC10799901

[B416] Zhao Y. Yue R. (2024). Aging adipose tissue, insulin resistance, and type 2 diabetes. Biogerontology 25 (1), 53–69. 10.1007/s10522-023-10067-6 37725294

[B417] Zhao X. Zhu X. Cheng S. Xie Y. Wang Z. Liu Y. (2014). MiR-29a/b/c regulate human circadian gene hPER1 expression by targeting its 3′UTR’. Acta Biochimica Biophysica Sinica 46 (4), 313–317. 10.1093/abbs/gmu007 24578160

[B418] Zhao M. Xing H. Chen M. Dong D. Wu B. (2020). Circadian clock-controlled drug metabolism and transport. Xenobiotica 50 (5), 495–505. 10.1080/00498254.2019.1672120 31544568

[B419] Zheng H. Wu T. Lin Z. Wang D. Zhang J. Zeng T. (2024). Targeting BMAL1 reverses drug resistance of acute myeloid leukemia cells and promotes ferroptosis through HMGB1-GPX4 signaling pathway. J. Cancer Res. Clin. Oncol. 150 (5), 231. 10.1007/s00432-024-05753-y 38703241 PMC11069489

[B420] Zhou J.-N. Liu R. Y. van Heerikhuize J. Hofman M. A. Swaab D. F. (2003). Alterations in the circadian rhythm of salivary melatonin begin during middle-age. J. Pineal Res. 34 (1), 11–16. 10.1034/j.1600-079x.2003.01897.x 12485366

[B421] Zhu Z. Li S. Yin X. Sun K. Song J. Ren W. (2024). Review: protein O-GlcNAcylation regulates DNA damage response: a novel target for cancer therapy. Int. J. Biol. Macromol. 264, 130351. 10.1016/j.ijbiomac.2024.130351 38403231

[B422] Zienolddiny S. Haugen A. Lie J. A. S. Kjuus H. Anmarkrud K. H. Kjærheim K. (2013). Analysis of polymorphisms in the circadian-related genes and breast cancer risk in Norwegian nurses working night shifts. Breast Cancer Res. 15 (4), R53. 10.1186/bcr3445 23822714 PMC3978690

[B423] Zilstorff D. B. Richter M. M. Hannibal J. Jørgensen H. L. Sennels H. P. Wewer Albrechtsen N. J. (2024). Secretion of glucagon, GLP-1 and GIP May be affected by circadian rhythm in healthy males. BMC Endocr. Disord. 24 (1), 38. 10.1186/s12902-024-01566-9 38481208 PMC10938734

[B424] Zimmet P. Alberti K. G. M. M. Stern N. Bilu C. El-Osta A. Einat H. (2019). The circadian syndrome: is the metabolic syndrome and much more. J. Intern. Med. 286 (2), 181–191. 10.1111/joim.12924 31081577 PMC6851668

[B425] Zsuga J. More C. E. Erdei T. Papp C. Harsanyi S. Gesztelyi R. (2018). Blind spot for sedentarism: redefining the diseasome of physical inactivity in view of circadian system and the Irisin/BDNF axis. Front. Neurology 9, 818. 10.3389/fneur.2018.00818 30333788 PMC6176117

